# 12th International Congress and 24th National Congress of the Spanish Society of Gerodontology (SEGER), Santiago de Compostela, Spain, 7–9 May 2026

**DOI:** 10.4317/medoral.1122335667809

**Published:** 2026-08-05

Abstracts of the scientific communications follow.

