# Supplementary material for: 12th International Congress and 24th National Congress of the Spanish Society of Gerodontology (SEGER), Santiago de Compostela, Spain, 7–9 May 2026
Source: Med Oral Patol Oral Cir Bucal. 2026 Aug 5;31(Suppl 2):S1–S81. doi: 10.4317/medoral.1122335667809 (PMC13589310; doi:10.4317/medoral.1122335667809)
Supplement: Related texts

# Abstract: 01. USE OF DEMINERALIZED DENTIN GRAFT AND PRP AS COMPLEMENTARY THERAPY IN THE EXTRACTION OF AN IMPACTED CANINE IN AN ADULT PATIENT

**Authors:** N. Sciaini Lewis, V. Sciaini Lewis, K. Rodríguez Pena, M. Quevedo Bissoni

**Affiliations:** *University of Santiago de Compostela. Email: natisciaini@hotmail.com*

The purpose of this clinical case is to evaluate the effects of demineralized dentin grafting (DDM) and PRP on alveolar bone preservation in a patient following the extraction of an impacted maxillary canine. The procedure involves cleaning and washing the tooth, which is mostly dentin, and then processing it using a specific piece of equipment called a Tooth Transformer. Dentin particles between 300 and 1200 µm are filtered through a special particle size system. These demineralized dentin particles, used as a graft after extractions, can be considered a quality standard for alveolar bone preservation, bone augmentation, and the treatment of bone defects. In addition to minimally invasive techniques and leveraging the regenerative power inherent in the patient's own tooth, we aim to shift the paradigm andoffer a reasonable alternative to techniques that pose greater risk and morbidity for the patient. Conclusion: Demineralized dentin grafting represents a new therapeutic option for clinical cases requiring a significant amount of graft material. Although medium- and long-term clinical and experimental studies are lacking, we believe that it yields a suitable graft material due to its composition, which is almost identical to that of human bone in terms of calcium and phosphorus ions organized as hydroxyapatite and TCP. We know that its organic phase contains abundant type I collagen and growth factors, and its characteristic microtubule organization promotes bone growth and osteoconduction. Furthermore, it has demonstrated superior performance compared to xeno-derivatives or other types of grafts. Based on the studies currently available, which as mentioned are limited, we can affirm that autologous dentin grafting does not provoke an immune reaction, therefore its biological and functional incorporation is rapid, effective and predictable.

# Abstract: 02. PSYCHOEMOTIONAL IMPACT, SLEEP DISTURBANCES AND PROFESSIONAL REPERCUSSION OF THE COVID-19 HEALTH CRISIS IN DENTISTS

**Authors:** M.S. Rey Martínez, N. Martínez Rodriguez, E.J. Castro Sabugueiro, D. Caamaño Martínez, C. Pedrouzo astro, M.H. Rey Martínez -

**Affiliations:** *University of Santiago de Compostela. Email: clinicadentalmares@gmail.com*

## Introduction

 The increasing number of implant procedures in the posterior maxilla has led to a higher incidence of complications involving the maxillary sinus. Between 25 and 40 percent of maxillary sinusitis cases are of odontogenic origin. Anatomical variations of the maxillary sinus, particularly septa and the course of the posterior superior alveolar artery, significantly influence surgical risk and graft prognosis. 

## Objectives

 To evaluate anatomical variations of the maxillary sinus using cone beam computed tomography and to analyze their impact on surgical planning and prevention of intraoperative complications. 

## Material and Methods

 Descriptive study based on systematic evaluation using high resolution cone beam computed tomography in patients undergoing implant surgery in the posterior maxilla. The presence and location of septa, thickness of the sinus membrane, residual bone height, course and diameter of the posterior superior alveolar artery, and root relationship to the sinus floor were analyzed. Modifications in the surgical plan derived from radiological assessment were recorded. 

## Results

 Sinus membrane perforation is the most frequently reported intraoperative complication in the literature. Preoperative identification of septa and intraosseous arterial pathways allowed modification of the lateral window design, optimization of surgical access, and reduction of hemorrhagic risk and membrane injury.

## Discussion and Conclusions

 Cone beam computed tomography should be considered an essential tool in posterior maxillary surgical planning. Individualized anatomical assessment reduces complications, improves surgical safety, and promotes coordinated management between dentistry and otorhinolaryngology.

# Abstract: 03 - IMPACT OF CONE BEAM COMPUTED TOMOGRAPHY PLANNING ON THE REDUCTION OF SINUS COMPLICATIONS IN POSTERIOR MAXILLARY IMPLANTOLOGY

**Authors:** C. Pedrouzo Castro, E.J. Castro Sabugueiro, M.H. Rey Martínez, N. Martínez Rodriguez, D. Caamaño Martínez, M.S. Rey Martínez

**Affiliations:** *University of Santiago de Compostela. Email: carla.pedrouzo@rai.usc.es*

## Introduction

 The increasing number of implant procedures in the posterior maxilla has led to a higher incidence of complications involving the maxillary sinus. Between 25 and 40 percent of maxillary sinusitis cases are of odontogenic origin. Anatomical variations of the maxillary sinus, particularly septa and the course of the posterior superior alveolar artery, significantly influence surgical risk and graft prognosis. 

## Objectives

To evaluate anatomical variations of the maxillary sinus using cone beam computed tomography and to analyze their impact on surgical planning and prevention of intraoperative complications. 

## Material and Methods

 Descriptive study based on systematic evaluation using high resolution cone beam computed tomography in patients undergoing implant surgery in the posterior maxilla. The presence and location of septa, thickness of the sinus membrane, residual bone height, course and diameter of the posterior superior alveolar artery, and root relationship to the sinus floor were analyzed. Modifications in the surgical plan derived from radiological assessment were recorded. 

## Results

 Sinus membrane perforation is the most frequently reported intraoperative complication in the literature. Preoperative identification of septa and intraosseous arterial pathways allowed modification of the lateral window design, optimization of surgical access, and reduction of hemorrhagic risk and membrane injury.

## Discussion and Conclusions

 Cone beam computed tomography should be considered an essential tool in posterior maxillary surgical planning. Individualized anatomical assessment reduces complications, improves surgical safety, and promotes coordinated management between dentistry and otorhinolaryngology.

# Abstract: 04 - SINUS MANIFESTATIONS AND OTOLARYNGOLOGICAL MANAGEMENT OF MEDICATION-RELATED OSTEONECROSIS OF THE MAXILLA IN OLDER ADULTS: A MULTIDISCIPLINARY CLINICAL EXPERIENCE

**Authors:** M.H. Rey Martínez, M.S. Rey Martínez, A. Sixto lvarellos, D. Caamaño Martínez, T. Lago Trillo, M.L. Perdomo Barrán

**Affiliations:** *Hospital Central de la Cruz Roja Madrid. Email: jelenrei@hotmail.com*

## Introduction

 Medication-related osteonecrosis of the jaw associated with antiresorptive or antiangiogenic agents represents a significant complication in older adults, particularly in patients with oncological disease or osteoporosis. Involvement of the maxilla may be associated with oroantral communication, secondary maxillary sinusitis and persistent sinonasal symptoms. An exclusively dental approach may underestimate sinus involvement. 

## Objectives

 To describe the otolaryngological manifestations associated with medication-related maxillary osteonecrosis in older adult patients and to analyze the clinical impact of multidisciplinary management. 

## Material and Methods

 Descriptive study including patients over 60 years of age diagnosed with medication-related maxillary osteonecrosis. Comprehensive dental assessment, otolaryngological examination with nasal endoscopy and paranasal sinus computed tomography when indicated were performed. Presence of secondary sinusitis, oroantral communication, need for complementary endoscopic surgery and clinical evolution after conservative or combined surgical treatment were analyzed, with a minimum six-month follow-up. 

## Results

 A relevant proportion of patients showed ipsilateral maxillary sinus involvement, with endoscopic findings of chronic inflammation. Joint evaluation enabled early identification of subclinical sinus compromise and optimization of therapeutic decision-making. Most cases demonstrated symptomatic improvement after coordinated management, with reduction of infectious complications. 

## Discussion

 Medication-related maxillary osteonecrosis in older adults should not be considered exclusively a dental condition, as it may involve sinus extension and associated otolaryngological symptoms. Interdisciplinary coordination reduces diagnostic delay and prevents unnecessary interventions. 

## Conclusions

 Multidisciplinary ENT–Dentistry management improves identification of sinus involvement in medication-related maxillary osteonecrosis and optimizes clinical outcomes in older adults, representing a reproducible care model.

# Abstract: 05 - IMPLANT FAILURE IN GERIATRIC PATIENTS UNDER SELECTIVE SEROTONIN REUPTAKE INHIBITOR THERAPY: CLINICAL IMPLICATIONS

**Authors:** K. Rodríguez Pena, V. Sciaini Lewis, M. Nuñez Otero, M. Quevedo Bisonni, J. Barbi Actis, J. Marcotegui

**Affiliations:** *University of Santiago de Compostela. Email: drakarenrodpena@gmail.com*

## Introduction

 Increased life expectancy has led to a growing number of geriatric patients undergoing implant-supported rehabilitation, often presenting with multiple comorbidities and polypharmacy. Among the most commonly prescribed medications are selective serotonin reuptake inhibitors, which may negatively influence bone metabolism and affect osseointegration and implant performance.

## Objectives

 To analyze the influence of long-term selective serotonin reuptake inhibitor use on the failure of osseointegrated implants, with particular focus on its relevance in geriatric patients.

## Material and Methods

 A retrospective cohort study was conducted in patients treated with titanium dental implants, with a minimum follow-up of 12 months. Patients under continuous selective serotonin reuptake inhibitor therapy for at least one year at the time of implant placement were included. Demographic, clinical and implant-related variables were analyzed. 

## Results

 A significant association was observed between continuous use of selective serotonin reuptake inhibitors and increased implant failure. Additional factors such as smoking, diabetes and implant length also influenced implant survival. 

## Discussion

 These findings suggest that drug-induced alterations in bone metabolism may impair bone remodeling in response to functional loading. In geriatric patients, where polypharmacy and reduced bone quality are common, this effect may have greater clinical relevance, supporting the need for more conservative implant planning strategies. 

## Conclusions

 Long-term use of selective serotonin reuptake inhibitors is associated with an increased risk of implant failure. In geriatric patients, this factor should be considered in treatment planning, particularly regarding loading protocols and long-term biomechanical management.

# Abstract: 06 - CLINICAL EVALUATION OF THE DIODE LASER VERSUS THE PUNCH TECHNIQUE IN THE SECOND SURGICAL PHASE OF OSSEOINTEGRATED IMPLANTS

**Authors:** E.J. Castro Sabugueiro, M.S. Rey Martínez, D. Caamaño Martínez, C. Pedrouzo Castro, M.H. Rey Martínez, N. Padrón González

**Affiliations:** *University of Santiago de Compostela. Email: mundo.castro@yahoo.es*

## Introduction

 The second surgical phase in oral implantology is a key procedure for the correct exposure of osseointegrated implants and the proper healing of peri-implant tissues. The use of the diode laser has been proposed as an alternative to conventional techniques, potentially providing clinical advantages in the management of soft tissues. 

## Objectives

 To compare the conventional punch technique with the use of the diode laser in the second surgical phase of osseointegrated implants. 

## Material and Methods

 A prospective observational study was carried out in 60 patients undergoing second-stage surgery. Two techniques were compared: punch and diode laser. Variables such as duration of the procedure, type of anesthesia, postoperative pain, need for analgesia, and inflammation perceived by the patient were evaluated. 

## Results

Differences were observed between both techniques in variables such as surgical time, postoperative pain, need for analgesia, and inflammation perceived by patients. In both groups, healing was favorable.

## Discussion

 The results support the use of the diode laser as an effective alternative due to its capacity for coagulation, disinfection, and lower tissue aggression. 

## Conclusions

 The diode laser is an effective technique in the second surgical phase, improving patient experience and clinical results, and can therefore be considered a valid alternative to the conventional technique.

# Abstract: 07 - ATYPICAL OROFACIAL PAIN IN OLDER ADULTS: CLINICAL DIFFERENTIAL DIAGNOSIS IN AN ENT–DENTISTRY COLLABORATIVE APPROACH

**Authors:** M.H. Rey Martínez, D. Caamaño Martínez, N. Padrón González, T. Lago Trillo, M.S. Rey Martínez, S. Pouso García

**Affiliations:** *Hospital Central de la Cruz Roja Madrid. jelenrei@hotmail.com*

## Introduction

 Chronic orofacial pain in older adults is a frequent reason for consultation and poses diagnostic challenges due to overlap among dental pathology, temporomandibular disorders, craniofacial neuralgias and otolaryngological disease. Lack of coordinated evaluation may prolong symptoms and lead to ineffective treatments. 

## Objectives

 To analyze the etiological causes of atypical orofacial pain in older adult patients and to assess the clinical impact of joint ENT–Dentistry evaluation on definitive diagnosis and symptomatic evolution. 

## Material and Methods

 Prospective study including 54 patients over 60 years of age with persistent orofacial pain without initial conclusive diagnosis. Comprehensive dental assessment, otolaryngological examination with upper airway endoscopy and targeted evaluation for neuropathic pain and temporomandibular dysfunction were performed. Final etiology, time to diagnosis and clinical response after specific treatment were recorded, with six-month follow-up. 

## Results

 Temporomandibular dysfunction was the most frequent cause, followed by craniofacial neuropathic pain and sinus pathology. Joint evaluation enabled definitive diagnosis in 91% of cases and reduced mean time to diagnosis to four weeks. Seventy-four percent of patients showed significant clinical improvement after targeted treatment.

## Discussion

 The multidisciplinary approach facilitates etiological identification of complex orofacial pain and prevents unnecessary investigations or prolonged empirical treatments. 

## Conclusions

 Coordinated ENT–Dentistry evaluation improves diagnostic accuracy and clinical outcomes in atypical orofacial pain among older adults, representing a high-impact care strategy.

# Abstract: 08 - DIRECT FIBER-REINFORCED COMPOSITE BRIDGES AS A MINIMALLY INVASIVE ALTERNATIVE IN GERIATRIC PATIENTS

**Authors:** C. Mourelle Nieto, M. Núñez Otero, G. Díaz Bastos, F.J. Gayo Morales

**Affiliations:** *University of Santiago de Compostela Email: correo@coelugo.org*

The loss of an anterior tooth creates an immediate need to restore aesthetics and function, as well as to prevent the psychological and social impact associated with such absence for the patient. Several treatment options are available for the replacement of a missing tooth, including removable partial dentures (RPD) and fixed partial dentures (conventional cemented fixed prostheses or implant-supported single crowns). The use of fiber-reinforced composite bridges may represent a more conservative approach for restoring a missing tooth. The aim of this undergraduate thesis is to present the clinical technique for the immediate replacement of an anterior tooth using a direct fiber-reinforced composite bridge, employing as a pontic a composite crown fabricated with prefabricated acetate crowns. This clinical technique is simple, cost-effective, non-invasive, and represents a suitable short- to medium-term provisional treatment option.

# Abstract: 09 - CLINICAL OUTCOMES OF A MULTIDISCIPLINARY ENT–DENTISTRY PROTOCOL IN THE MANAGEMENT OF OROANTRAL FISTULA AND ODONTOGENIC SINUSITIS IN ADULT PATIENTS

**Authors:** M.H. Rey Martínez, M.S. Rey Martínez, T. Lago Trillo, N. Martínez Rodríguez, S. Pouso García, E.J. Castro Sabugueiro

**Affiliations:** *Hospital Central de la Cruz Roja Madrid. Email: jelenrei@hotmail.com*

## Introduction

 Oroantral fistula associated with odontogenic sinusitis is a common condition requiring coordinated diagnostic and therapeutic management between dentistry and otorhinolaryngology. Non-protocolized approaches may lead to persistent infection, chronic inflammation and therapeutic failure. 

## Objectives

 To evaluate the clinical efficacy and outcome stability of a multidisciplinary protocol for the sequential diagnosis and treatment of oroantral fistula associated with odontogenic sinusitis in adult patients. 

## Material and Methods

 Prospective study including 48 patients with confirmed diagnosis based on clinical evaluation, nasal endoscopy and paranasal sinus computed tomography with dental sections. The protocol integrated initial dental assessment, targeted antibiotic therapy, surgical closure of the oroantral communication when indicated and endoscopic sinus surgery in cases of persistent involvement. Complete clinical resolution, time to resolution, reintervention, complications and recurrence during six months were analyzed. 

## Results

 Complete resolution was achieved in 92% of patients (95% confidence interval: 84–97), with a mean time to resolution of five weeks. Surgical closure was required in 69% and complementary endoscopic sinus surgery in 35%. Complications were low, with reintervention below 6% and recurrence rate of 4%. 

## Discussion

 Coordination between dentistry and otorhinolaryngology enables precise etiological diagnosis and staged treatment, reducing therapeutic failures and unnecessary procedures. 

## Conclusions

 The absence of a multidisciplinary protocol represents an avoidable risk factor for treatment failure. Its implementation demonstrates high clinical efficacy, low morbidity and stable outcomes, optimizing antibiotic use and reducing unnecessary interventions, establishing a reproducible model for comprehensive management of oroantral fistula and odontogenic sinusitis.

# Abstract: 10 - ORAL AND LARYNGEAL MANIFESTATIONS OF LARYNGOPHARYNGEAL REFLUX IN OLDER ADULTS: A CORRELATIONAL CLINICAL ENT–DENTISTRY STUDY

**Authors:** M.H. Rey Martínez, M.S. Rey Martínez, E.J. Castro Sabugueiro, C. Pouso García, D. Caamaño Martínez, C. Pedrouzo Castro

**Affiliations:** *Hospital Central de la Cruz Roja Madrid. University of Santiago de Compostela. Email: jelenrei@hotmail.com*

## Introduction

 Laryngopharyngeal reflux in older adults may present with nonspecific symptoms such as chronic nonproductive cough, throat clearing and foreign body sensation without typical heartburn or retrosternal burning. Clinical recognition is challenging and may be misdiagnosed as chronic pharyngitis.

## Objectives

 To analyze the correlation between fiberoptic laryngoscopic findings suggestive of laryngopharyngeal reflux and oral manifestations in older adult patients. 

## Material and Methods

 Prospective study including 41 patients over 60 years of age with symptoms compatible with laryngopharyngeal reflux. Fiberoptic laryngoscopy was performed to identify posterior laryngitis, interarytenoid pachydermia and interarytenoid edema, together with dental evaluation to detect acid-related dental erosions. Clinical follow-up was conducted for three months after medical treatment. 

## Results

 Posterior laryngitis with interarytenoid changes was observed in 46% of patients and 49% presented dental erosions compatible with chronic acid exposure. After medical treatment, 61% showed significant improvement in cough and throat clearing. 

## Discussion

 The correlation between posterior laryngeal findings and dental lesions supports the extraesophageal nature of reflux and highlights the importance of an interdisciplinary approach. 

## Conclusions

 Clinical recognition of laryngopharyngeal reflux through fiberoptic laryngoscopy and oral evaluation improves diagnostic accuracy and symptomatic evolution in older adults.

# Abstract: 11 - CLINICAL SUCCESS OR PERCEIVED SUCCESS? QUALITY OF LIFE AND SATISFACTION IN ELDERLY PATIENTS REHABILITATED WITH IMPLANTS

**Authors:** M. Núñez Otero, J. Barbi Actis, M. Quevedo Bisonni, K. Rodriguez Pena, V. Sciaini Lewis

**Affiliations:** *Faculty of Dentistry and Medicine of Santiago de Compostela. Email: clinicanunezotero@gmail.com*

## Introduction

 Implant rehabilitation is a predictable treatment with high success rates. In elderly patients, success includes quality of life and subjective perception. 

## Objectives

 To evaluate satisfaction and quality of life after more than two years of function and to analyze functional, aesthetic, and psychosocial factors.

## Material and Methods

 A cross-sectional study was conducted including patients rehabilitated with different types of implant-supported prostheses with at least two years of evolution. 

## Results

 High overall satisfaction and improved quality of life were observed. Aesthetic and functional aspects were the most valued. Differences were found between patient and professional perception. 

## Discussion

 In elderly patients, treatment success depends not only on clinical parameters but also on individual perception and expectations. 

## Conclusions

 Implants improve quality of life. Patient perception is key to treatment success.

# Abstract: 12 - OTOLOGIC SYMPTOMS ASSOCIATED WITH TEMPOROMANDIBULAR JOINT DISORDERS IN OLDER ADULTS: A PROSPECTIVE ENT–DENTISTRY CLINICAL STUDY

**Authors:** M.H. Rey Martínez, N. Martínez Rodríguez, C. Pedrouzo Castro, M.S. Rey Martínez, E.J. Castro Sabugueiro, A. Sixto lvarellos

**Affiliations:** *Hospital Central de la Cruz Roja Madrid. Email: jelenrei@hotmail.com*

## Introduction

 Temporomandibular joint dysfunction is a frequent cause of orofacial pain in older adults and may be associated with nonspecific otologic symptoms such as otalgia, aural fullness and tinnitus. Clinical overlap with primary otologic pathology complicates differential diagnosis and may lead to unnecessary referrals. 

## Objectives

 To determine the prevalence of otologic symptoms in patients with temporomandibular dysfunction and to evaluate the clinical impact of joint otolaryngological and dental assessment. 

## Material and Methods

 Prospective study including 62 patients over 55 years of age with clinical diagnosis of temporomandibular dysfunction. Comprehensive otolaryngological examination, functional dental evaluation and systematic symptom recording was performed. Otalgia, aural fullness, tinnitus, mandibular limitation and muscular pain were analyzed. Three-month clinical follow-up after conservative treatment was conducted. 

## Results

 Referred otalgia was present in 58% of patients, aural fullness in 46% and tinnitus in 29%, with no structural otologic findings in most cases. After conservative dental treatment, 67% showed significant improvement in otologic symptoms and reduction of orofacial pain. 

## Discussion

 The high frequency of otologic symptoms in temporomandibular dysfunction confirms the close anatomical and functional relationship between cranio-mandibular structures and the middle ear, supporting the need for joint evaluation to avoid overtreatment. 

## Conclusions

 Temporomandibular pathology should be considered a priority differential diagnosis in older adults presenting with nonspecific otologic symptoms. ENT–Dentistry collaboration improves diagnostic accuracy and optimizes clinical outcomes.

# Abstract: 13 - IMPACT OF A MULTIDISCIPLINARY OTOLARYNGOLOGY–DENTISTRY PROTOCOL ON THE TREATMENT OF SNORING IN OLDER ADULTS: PROSPECTIVE CLINICAL OUTCOMES

**Authors:** M.H. Rey Martínez, M.S. Rey Martínez, M.L. Pedormo Barrán, A. Sixto lvarellos, C. Pedrouzo Castro, E.J. Castro Sabugueiro

**Affiliations:** *Hospital Central de la Cruz Roja Madrid. Email: jelenrei@hotmail.com*

## Introduction

 Snoring in older adults is a common clinical problem with functional, social and cardiovascular implications. Its multifactorial origin requires comprehensive evaluation of the upper airway and cranio-mandibular structure. The absence of coordinated protocols between specialties may lead to incomplete or ineffective treatments. 

## Objectives

 To evaluate the clinical efficacy of a joint multidisciplinary protocol between otolaryngology and dentistry for etiological diagnosis and stepwise treatment of snoring in older adult patients. 

## Material and Methods

 Prospective descriptive study including 59 patients over 60 years of age with habitual snoring. A structured clinical pathway included sleep assessment using the Epworth Sleepiness Scale and the Pittsburgh Sleep Quality Index, otolaryngological examination with upper airway endoscopy, and occlusal and functional oral evaluation. Individualized therapeutic planning incorporated positional and hygienic measures, medical treatment and mandibular advancement devices adapted to each patient’s periodontal and prosthetic condition. Snoring intensity as perceived by the bed partner and sleep quality were assessed after three months of follow-up. 

## Results

The protocol identified predominant nasal obstruction in 32%, oropharyngeal obstruction in 41% and mixed pattern in 27% of patients. Sixty-eight percent of patients treated with mandibular advancement devices showed significant reduction in snoring according to bed partner evaluation. Overall satisfaction exceeded 80% and therapeutic adherence was high. 

## Discussion

 Joint evaluation improves diagnostic accuracy and allows selection of the most appropriate treatment according to the level of airway collapse. In gerodontology, device design must consider dental fragility and the presence of prostheses to ensure therapeutic success. 

## Conclusions

 The multidisciplinary approach represents an effective and reproducible care model for the treatment of snoring in older adults, with high acceptance and clinically relevant outcomes.

# Abstract: 14 - PREOPERATIVE DETERMINATION OF THE GRAFT VOLUME REQUIRED IN MAXILLARY SINUS LIFT SURGERY

**Authors:** D. Caamaño Martínez, N. Padrón González, M.S. Rey Martínez, M.H. Rey Martínez, E.J. Castro Sabugueiro, C. Pedrouzo Castro

**Affiliations:** *University of Santiago de Compostela Email: domingocaamano@gmail.com*

## Introduction

 One of the aspects that we must take into account when planning a maxillary sinus lift surgery is the amount of graft material that we will need to achieve an adequate increase in bone availability at this level. 

## Objectives

 The main objective of the present study is to calculate, in a presurgical way, the graft volume necessary to perform a maxillary sinus lift using the open or side window technique, and the subsequent rehabilitation using 12 mm dental implants of length. 

## Material and Methods

 We used CBCT images of 68 patients, 26 men and 42 women, aged between 40 and 76 years, with an average of 60 years. A total of 116 maxillary sinuses were studied. To determine the necessary graft volume in each case, we used ITK-SNAP® software, designed for the segmentation of structures in medical images. 

## Results

 The mean graft volume necessary to achieve a height of 13 mm was 1321.5 mm³ for the right maxillary sinus, with a range of 366.4 to 3178.0. For the left maxillary sinus, the mean volume was 1503.5 mm³, with a range of 308.3 to 5940.0 mm³. 

## Discussion and Conclusions

 We consider that the ITK-SNAP® software is a useful tool in the planning of a maxillary sinus lift surgery, since it allows us to calculate the graft volume in mm³ in a presurgical way, as well as its visualization in 3D.

# Abstract: 15 - XEROSTOMIA, ORAL BREATHING AND ASSOCIATED ORAL PATHOLOGY IN OLDER ADULTS: AN OBSERVATIONAL ENT–DENTISTRY CLINICAL STUDY

**Authors:** M.H. Rey Martínez, M.S. Rey Martínez, T. Lago Trillo, E.J. Castro Sabugueiro, N. Padrón González, D. Caamaño Martínez

**Affiliations:** *Hospital Central de la Cruz Roja Madrid. Email: jelenrei@hotmail.com*

## Introduction

 Xerostomia in older adults is associated with root caries, mucosal infections and functional impairment. Chronic oral breathing secondary to nasal obstruction may aggravate oral dryness and promote dental pathology. 

## Objectives

 To analyze the relationship between chronic oral breathing and clinical manifestations of xerostomia in older adult patients. 

## Material and Methods

 Observational study including 70 patients over 60 years of age with symptoms of oral dryness. Clinical dental evaluation, salivary flow measurement and otolaryngological examination were performed to detect structural or functional nasal obstruction. Root caries, oral candidiasis and xerostomic medication were recorded. Three-month follow-up was conducted. 

## Results

 Chronic oral breathing was present in 61% of patients and significant nasal obstruction in 48%. Moderate to severe xerostomia was more frequent in these individuals. The prevalence of root caries was 42% and oral candidiasis 18%. 

## Discussion

Persistent oral breathing acts as an aggravating factor of xerostomia and should be considered in the comprehensive clinical evaluation of older adults. 

## Conclusions

 Identification and treatment of nasal obstruction may contribute to improving oral health and reducing complications associated with xerostomia.

# Abstract: 16 - REHABILITATION OF COMPLEX BONE ATROPHIES USING SUBPERIOSTEAL IMPLANTS WITH A DIGITAL WORKFLOW: A CASE REPORT

**Authors:** K. Ivaylova Serkedzhieva, P. Benito López, S. de Córdoba Fernández, A. Flores Fernández, C. Madrigal Martínez-Pereda, J. López-Quiles

**Affiliations:** *Postgraduate Program in Oral Surgery and Implantology, Faculty of Dentistry, Complutense University of Madrid Email: katerinaivaylova@gmail.com*

## Introduction

 New-generation subperiosteal implants are indicated in patients with severe maxillary or mandibular atrophy when conventional endosseous implants are not feasible without extensive bone grafting procedures. Their main advantages include the absence of donor site morbidity, reduced treatment time, and the possibility of immediate prosthetic loading. 

## Objectives

 To describe the clinical and surgical approach for the rehabilitation of severe bone atrophy using customized subperiosteal implants within a fully digital workflow, as well as to analyze the role of digital planning and the materials used in this type of treatment. Materials and Methods: A clinical case is presented along with a narrative review of the literature on materials used in subperiosteal implants. Planning was performed using helicoidal CT, with DICOM files sent for CAD design and subsequent manufacturing via 3D printing. 

## Results

 A 71-year-old patient presented with severe mandibular bone resorption (Cawood and Howell class V). Bilateral subperiosteal frameworks were planned in the posterior mandibular regions (3.4–3.6 and 4.4–4.6). Following flap elevation, a resin prototype was used to verify fit and prepare cortical screw sites. The definitive structure was fixed using self-drilling screws (six per quadrant). Soft tissue repositioning and suturing were performed, followed by immediate loading with provisional PMMA restorations.

## Discussion and Conclusions

 Digitally planned subperiosteal implants represent a predictable and less invasive alternative for the treatment of severe bone atrophy. Currently, they are mainly manufactured using titanium alloy (Ti6Al4V) through additive technologies such as direct metal laser sintering (DMLS), allowing high precision and mechanical strength; historically, cobalt-chromium alloys and hydroxyapatite coatings were also used. Proper patient selection and rigorous digital planning are essential. Long-term studies are needed to confirm their clinical effectiveness.

# Abstract: 17 - BACKWARD PLANNING PROTOCOL FOR DEPENDENT PATIENTS WITH COMPLEX Oral REHABILITATIONS: A CLINICAL PROPOSAL FOR THERAPEUTIC PLANNING AND SIMPLIFICATION

**Authors:** F. Franzani Osorio

**Affiliations:** *Faculty of Dentistry, Universidad de Talca, Chile Email: francisco.franzani@utalca.cl*

## Introduction

 Increased life expectancy has prolonged the functional lifespan of complex oral rehabilitations. However, the onset of physical and cognitive decline may compromise oral hygiene and regular follow-up, transforming these rehabilitations into a source of mechanical and biological complications. 

## Objectives

 To propose a clinical protocol for prognostic assessment and backward planning in patients with existing or planned complex oral rehabilitations, aimed at anticipating long-term sustainability and systematizing therapeutic simplification when required by the patient’s condition. Materials and Methods: A clinical protocol was developed from a narrative review of the international gerodontology literature. Validated instruments addressing functional dependence, frailty, cognition, oral health status, nutrition, swallowing, and quality of life were integrated into a decision-making algorithm and progressive levels of therapeutic simplification. 

## Results

 A two-phase protocol was designed. The first phase consists of a multidimensional prognostic assessment to support decisions regarding the indication, maintenance, or limitation of complex rehabilitations. The second phase establishes a backward planning sequence that includes treatment simplification, prosthetic conversion, and, in advanced cases, functional deactivation of implants. 

## Discussion

 The proposed model shifts the concept of treatment success from technical complexity to clinical sustainability, incorporating the patient’s ageing trajectory and future dependency into rehabilitation planning. 

## Conclusions

 Backward planning should be understood as an ethical and proactive therapeutic strategy. A successful rehabilitation is not only one that functions adequately in the present, but one that can be adapted to future dependency without becoming a greater burden than the original condition.

# Abstract: 18 - USE OF COMBINED THERAPY IN BILATERAL MASSETER HYPERTROPHY

**Authors:** A. Vera Estefani

**Affiliations:** *University of Santiago de Compostela Email: aliciaverast@gmail.com*

## Introduction

 The etiology of this clinical condition is multifactorial and may be related to numerous processes such as bruxism, specific pathology of the temporomandibular joint, occlusion alterations, emotional disorders, chronic stress, absence of REM sleep, as well as a specific alteration in the modulation of acetylcholine and dopamine in the nociceptors and muscle neuroreceptors of one or both masseter muscles. The initial clinical manifestations are very variable, most of them are asymptomatic and are usually diagnosed after the patient perceives a variable bulge in the proximities of the mandibular angle. This anatomical modification and disfigurement can be perceived by the patient with greater or lesser intensity, depending on the variations in weight that he/she may experience during a short period of time. It should also be noted that not in all unilateral or bilateral masseter hypertrophies is indicated, and that in some cases, it can lead to the undesirable circumstance of overtreatment, with the consequent masking of other pathologies that should have been diagnosed by the professional through the prior prescription of other complementary diagnostic tests. 

## Clinical case

 A 40-year-old female patient came to the Oral Surgery Teaching Unit of the University of Santiago de Compostela, referred by her dentist, who had previously treated her for bruxism and TMJ joint dysfunction with an anterior joint repositioning splint. The patient has been reporting for two years the existence of a progressive, bilateral and sometimes painful muscular bulging in the area of the masseter muscles. Although a clear differential diagnosis with other pathologies could be established by inspection and clinical history, we indicated conventional imaging tests, magnetic resonance imaging and ultrasound of both parotid spaces and the area of both masseter muscles. 

## Conclusions

 The combined use of conservative occlusal therapy using anterior repositioning splints and injections with toxin botulinum constitutes an effective therapy for controlling the aesthetic manifestations of bilateral masseter muscles hypertrophy.

# Abstract: 19 - PIPERACILLIN/TAZOBACTAM TREATMENT IN PATIENTS WITH STAGE III-IV PERIODONTITIS

**Authors:** D. Hurtado Celotti, E. Cañaveral Cavero, L. Sánchez Labrador, T. Beca Campoy, P.L. Ruiz Sáenz, M.H. Rey Martínez

**Affiliations:** *Universidad Complutense de Madrid. Email: lhcelotti@gmail.com*

## Objectives

 To evaluate the clinical and microbiological outcomes of piperacillin/tazobactam administration as an adjunct to non-surgical treatment in patients with periodontitis. 

## Material and Methods

 26 patients were included in a randomized, split-mouth, single-blind clinical trial with 6 months of follow-up. All patients underwent scaling and root planing. The topical antibiotic was randomly applied to one side of the mouth. 

## Results

 24 patients completed the study and were included in the analysis. Significant differences were observed, with the combined treatment showing a more favorable outcome, in clinical attachment level at 3 months (p = 0.001) and 6 months (p = 0.002); and in plaque index at 15 days (p = 0.000), 3 months (p = 0.002), and 6 months (p = 0.000). in the gingival index at 6 months (p = 0.034) and in probing depth at 3 months (p = 0.024) and 6 months (p = 0.004). Regarding the microbiological results, a significant difference favoring the combined treatment was only observed for Treponema denticola at 3 months. 

## Conclusions

 Topical application of piperacillin/tazobactam as an adjunct to conventional treatment could lead to an improvement in periodontal clinical parameters in the short term. Further studies are needed to evaluate its long-term efficacy.

# Abstract: 20 - ODONTOLOGICAL–PROSTHETIC MANAGEMENT OF MAXILLARY DEFECTS WITH ORONASAL COMMUNICATION IN POST-ONCOLOGIC PATIENTS: LITERATURE REVIEW

**Authors:** J. Larrosa Henríquez, V. Gil, N. Larrosa, B. Olmo

**Affiliations:** *Universitat Internacional de Catalunya (UIC Sant Cugat), Barcelona, España. Email: lhcelotti@gmail.com*

## Introduction

 Maxillary defects produced after oncologic treatment may cause oronasal communication with significant functional impairment (speech, swallowing, and mastication). In polymedicated or medically compromised patients, rehabilitation with maxillary obturator prostheses constitutes a minimally invasive and clinically relevant alternative to resolve oronasal communication and improve quality of life.

## Objectives

 To evaluate functional outcomes and the degree of clinical success of maxillary obturator prostheses in post-oncologic patients with oronasal communication and to optimize practical criteria for odontological prosthetic management. 

## Material and Methods

 Systematic review following PRISMA 2020 reporting criteria. Searches were conducted in biomedical and dental databases (PubMed/MEDLINE, Scopus, Web of Science, and dentistry-specific sources available at UIC Sant Cugat) for the period 2000–2026, combining terms related to maxillectomy/maxillary defect, oronasal communication, and maxillary obturator. Human studies with clinical evaluation of obturators (function, quality of life, retention/stability, complications, or follow-up) were included. Studies were screened by titles and abstracts; full-text assessments, deduplication, and those lacking structured narrative synthesis were performed. Methodsological quality was assessed using tools appropriate to the study design. 

## Results

 Available evidence shows that rehabilitation with maxillary obturators is generally associated with improved oronasal sealing and functional recovery, with a favorable impact on quality of life. Clinical success depends on multiple factors: the defect (extent and available support), prosthetic design and retention system, as well as the patient’s tolerance and capacity for maintenance. The most frequently reported complications are related to loss of retention, tissue irritation, leakage, and the need for periodic adjustments.

## Discussion

 Heterogeneity of designs and outcome measures limits direct comparisons; nevertheless, the body of evidence supports the obturator as an effective and accessible rehabilitative option in contexts where a more invasive approach is not feasible. Individualized planning and structured follow-up are key determinants. 

## Conclusions

 Maxillary obturator prostheses represent an effective and minimally invasive dental alternative for post-oncologic patients with oronasal communication. A clinical roadmap is proposed to guide prosthetic selection and maintenance.

# Abstract: 21 - INFLUENCE OF OSSEODENSIFICATION ON PRIMARY IMPLANT STABILITY IN LOW-DENSITY BONE: APPLICATION IN GERONTOLOGY

**Authors:** A. García Marqués, P. Maguregui Ortiz, K. Ivaylova Serkedzhieva, A. González Martínez, G. Ochoa López, C. Madrigal Martínez-Pereda

**Affiliations:** *Complutense University of Madrid Email: alan.garcia.marques2001@gmail.com*

## Introduction

 The increasing number of geriatric patients with osteoporosis and low bone density poses a challenge in implant dentistry, where primary stability is critical. Factors such as age, sex, vitamin deficiencies (vitamin D), and the use of medications such as proton pump inhibitors have been associated with poorer implant outcomes. Osseodensification is proposed as a predictable technique to optimize the bone–implant contact surface. 

## Objectives

 To compare the primary stability of implants placed using conventional technique versus osseodensification in low-density bone, and to assess its relevance in geriatric patients. 

## Material and Methods

 Ex vivo study in low-density porcine bone (type III–IV) in which implants were placed using conventional technique and osseodensification. Insertion torque, removal torque, and implant stability quotient (ISQ) were evaluated. Descriptive and inferential statistical analyses were performed. 

## Results

 Osseodensification showed significantly higher values in terms of insertion and removal torque, as well as ISQ values (p < 0.05), demonstrating an improvement in primary stability. Positive correlations were observed among the analyzed variables. 

## Discussion

 Primary stability is a determining factor in implant success, especially in older patients with low-quality bone, where the risk of micromovements and implant failure is higher. The osseodensification technique using Densah burs allows bone compaction and increased bone–implant contact, which could be particularly relevant in patients with osteoporosis and those undergoing pharmacological treatments that affect bone metabolism. 

## Conclusions

 Osseodensification significantly improves primary stability in low-density bone, positioning itself as a predictable strategy in geriatric implant dentistry. Its application could simplify treatments by avoiding regenerative surgeries in older patients with potential limitations or systemic diseases, although further clinical studies are needed.

# Abstract: 22 - APPLICATION OF ARTIFICIAL INTELLIGENCE FOR THE DIAGNOSIS OF ORAL LESIONS IN ELDERLY PATIENTS: PRELIMINARY RESULTS FROM A DOCTORAL THESIS

**Authors:** K. Ivaylova Serkedzhieva, A. Redondo García, M. Bachiller Mayoral, M. Rincón Zamorano, J.M. López-Cuadra, R.M. López-Pintor Muñoz

**Affiliations:** *Facultad de Odontología, Universidad Complutense de Madrid Email: katerinaivaylova@gmail.com*

## Introduction

 Oral cancer in elderly patients represents a growing clinical challenge, with up to 40–50% of cases diagnosed in individuals over 65 years and poorer survival rates compared to younger populations. Early detection of oral potentially malignant disorders (OPMDs) is essential but challenging, highlighting the need for artificial intelligence (AI)-based tools. 

## Objectives

 To develop and evaluate an AI-based system for the detection and classification of oral lesions, emphasizing its applicability in elderly patients. 

## Material and Methods

 The project was structured into four phases: (1) collection and processing of more than 8,000 clinical images and associated variables; (2) training and validation of deep learning models to classify lesions into four categories (healthy, benign, potentially malignant, and malignant), using a 70/20/10 dataset split; (3) development of a Bayesian regression model and integration into a mobile application; and (4) system evaluation and planning for clinical implementation. 

## Results

 The ConvNeXt v2 Tiny model achieved the best performance, with 85.53% accuracy and 85.02% precision using 384×384 pixel images. The system is currently undergoing external validation using unseen clinical cases, evaluated by both AI and clinicians to compare diagnostic performance. 

## Discussion and Conclusions

 AI may improve screening and early diagnosis in elderly patients. Despite limitations such as dataset heterogeneity, the system shows strong potential as a clinical support tool, requiring further validation in geriatric populations.

# Abstract: 23 - EMERGING DRUGS IMPLICATED IN MEDICATION RELATED OSTEONECROSIS OF THE JAWS. A REVIEW OF CURRENT EVIDENCE

**Authors:** C.J. Guerra de la Cruz, A. Bowen Varela, M. López-Justo, C. Cobo Vázquez, J. López-Quiles, L.M. Sáez-Alcaide

**Affiliations:** *Facultad de Odontología, Universidad Complutense de Madrid Email: caguer03@ucm.es*

## Introduction and Objectives

 Medication related osteonecrosis of the jaws has traditionally been associated with the use of antiresorptive drugs, such as bisphosphonates and denosumab. However, in recent years, cases linked to other non-antiresorptive pharmacological groups have been reported, particularly antiangiogenic and immunomodulatory agents. The aim of this study is to review the available evidence regarding the potential association between these drugs and the development of osteonecrosis of the jaws, as well as to analyze the underlying pathophysiological mechanisms. 

## Material and Methods

 A literature review was conducted through searches in the Medline, Scopus, and Embase databases. Articles published in English and Spanish over the last 10 years were included, using terms related to “osteonecrosis of the jaw,” “MRONJ,” “antiangiogenic agents,” “monoclonal antibodies,” and “immunomodulators.”

## Results

 The available evidence suggests that antiangiogenic drugs, such as bevacizumab and tyrosine kinase inhibitors, are the non-antiresorptive agents most consistently associated with medication related osteonecrosis of the jaws, likely due to their ability to inhibit angiogenesis and impair bone healing. Furthermore, immunomodulatory drugs, including tumor necrosis factor inhibitors (infliximab, etanercept) and other monoclonal antibodies, such as rituximab and tocilizumab, have also been associated with this condition, particularly in the context of combination therapy or in the presence of concomitant risk factors.

## Conclusions

 Although medication-related osteonecrosis of the jaws remains primarily associated with antiresorptive drugs, there is growing evidence implicating other non-antiresorptive agents, especially antiangiogenic drugs. Knowledge of these medications is essential for the prevention and clinical management of patients with polypharmacy patients, particularly in the field of gerodontology.

# Abstract: 24 - SURGICAL COMPLICATIONS IN GERIATRIC PATIENTS: FACTORS ASSOCIATED WITH THEIR OCCURRENCE

**Authors:** D. Sahli, J. Jimenez Aracil, A. Martínez Romero, A. García Marqués, E. Ramos Rodríguez, C. Cobo Vázquez, J. López-Quiles

**Affiliations:** *Department of Oral Surgery and Implantology, Faculty of Dentistry, Complutense University of Madrid Email: dosahli@ucm.es*

## Introduction

 The growing geriatric population poses new challenges in oral surgery, where the occurrence of complications is not solely dependent on chronological age, but also on associated clinical factors that are often underestimated. 

## Objectives

 To review the most relevant surgical complications in geriatric patients and to analyze the factors associated with their occurrence. 

## Material and Methods

 A literature review was conducted in PubMed using terms related to elderly patients, oral surgery, and postoperative complications, focusing on the last 10 years. A total of 58 results were obtained, of which 15 articles were selected after applying criteria of clinical relevance and alignment with the study objectives. 

## Results

 The most frequent complications identified were postoperative bleeding, infection, delayed healing, and medication-related osteonecrosis. The main associated factors included polypharmacy, particularly anticoagulants and antiresorptive drugs, systemic comorbidities, the patient’s functional status, and the type of procedure performed. 

## Discussion

 The findings highlight that age alone is not sufficient to estimate surgical risk. In clinical practice, patient variability is considerable, requiring the integration of factors such as medication use, overall health status, and recovery capacity. These elements influence both the indication and planning of treatment. 

## Conclusions

 Recognizing the factors that contribute to complications allows for better-informed decision-making in geriatric patients. An individualized assessment supports a more cautious and clinically appropriate management approach for each patient.

# Abstract: 25 - SUBLINGUAL AUTOVACCINE FOR RECURRENT ORAL CANDIDIASIS: A PILOT

**Authors:** A. Pérez Jardón, E.M. Otero Rey, A. Blanco Carrión, P. Gándara Vila, A.V. Blanco Besteiro, M. Pérez Sayáns

**Affiliations:** *Oral Medicine, Oral Surgery and Implantology Unit, Med Oral Res Group, University of Santiago de Compostela, Santiago de Compostela, Spain. Email: perlopjm@gmail.com*

## Introduction

 Oral candidiasis is an opportunistic infection caused primarily by Candida albicans, whose recurrent nature poses a significant clinical challenge. Conventional antifungal therapies are limited by high recurrence rates and systemic toxicity. Autovaccines represent a promising complementary approach. 

## Objectives

 To assess the clinical and microbiological efficacy of a patient-derived sublingual autovaccine (Vacucis Candida®) in recurrent, treatment-refractory oral candidiasis. Methods: A pilot study conducted in accordance with Consolidated Standards of Reporting Trials recommendations. Nine adult patients with clinically and microbiologically confirmed recurrent oral candidiasis were enrolled. The autovaccine was administered sublingually nightly for seven consecutive weeks. Outcome measures included quantitative fungal cultures expressed as colony-forming units per millilitre at 24, 48 and 72 hours, the Visual Analogue Scale, the Oral Health Impact Profile, the Xerostomia Inventory, and unstimulated and stimulated salivary flow. 

## Results

 All patients completed treatment. A progressive reduction in fungal burden was observed, reaching statistical significance from week six onward (p < 0.05). Pain and quality of life scores showed sustained improvement. Xerostomia perception decreased and salivary flow showed an upward trend. No adverse effects were recorded. Women exhibited a delayed reduction in culture positivity. 

## Discussion

 These findings are consistent with prior evidence in vulvovaginal candidiasis, supporting the potential of autovaccines to induce a specific immune response and reduce fungal burden. 

## Conclusions

 The sublingual autovaccine proved well tolerated and was associated with meaningful clinical improvement and reduced culture positivity. However, the small sample size, clinical heterogeneity and absence of a control group limit generalisation. Larger randomised controlled trials are warranted to confirm these preliminary results.

# Abstract: 26 - COMBINED REHABILITATION IN THE ELDERLY EDENTULOUS PATIENT: MAXILLARY COMPLETE DENTURE AND MANDIBULAR HYBRID PROSTHESIS AS A PREDICTABLE OPTION: A CASE REPORT

**Authors:** C. Pérez Arnau, L. Minguito Alaminos, C. Tobar, J. Pelaìez Rico, M.J. Suarez Garciìa

**Affiliations:** *Master’s Degree in Oral Prosthodontics and Occlusion at the Complutense University of Madrid. Email: carolp12@ucm.es*

## Introduction and Objectives

 Total edentulism in older adults significantly compromises function, aesthetics, and quality of life. Literature shows that implant-supported prostheses provide greater stability and satisfaction than removable dentures, especially in the lower arch. The objective of this clinical case was to rehabilitate a patient by combining a removable prosthesis with a fixed prosthesis, describing the clinical protocol and results. Methodology An 80-year-old patient attended the clinic of the Master’s Degree in Oral Prosthodontics and Occlusion at the Complutense University of Madrid to rehabilitate both edentulous jaws. After clinical, radiographic, and photographic evaluation, a maxillary complete removable denture and an implant-supported mandibular prosthesis were planned to balance stability, hygiene, and patient expectations. Maxillomandibular relationship was recorded, vertical dimension of occlusion and centric relation were determined and verified, and aesthetic and functional try-ins were conducted before final prosthesis fabrication. 

## Results

 After placement of the maxillary complete denture and the mandibular fixed hybrid prosthesis, the patient showed significant improvement in stability, masticatory function, speech, and aesthetics, without biological or mechanical complications, requiring only minor occlusal adjustments during initial follow-up. 

## Discussion

 The combination of a well-designed removable maxillary prosthesis with a fixed implant-supported mandibular restoration has been described as a predictable and effective solution to improve oral function in edentulous patients with severe resorption and previous failures of lower dentures. Therefore, the choice between fixed and removable rehabilitations should be individualized according to anatomy, hygiene capacity, and expectations. 

## Conclusions

 The combination of a maxillary complete removable denture and a mandibular fixed hybrid prosthesis is a reliable rehabilitation option for edentulous elderly patients, improving function, aesthetics, and quality of life when the prosthetic type is appropriately selected.

# Abstract: 27 - SERUM VITAMIN D DEFICIENCY AND INSUFFICIENCY AND EARLY FAILURE IN DENTAL IMPLANTOLOGY

**Authors:** X. Hu Liu, S. Prin, M. Albánchez, M.J. Suárez García, J. Peláez Rico

**Affiliations:** *Máster Prótesis Bucofacial y Oclusión – Universidad Complutense de Madrid. Email: xinlliu22@gmail.com*

## Introduction

 vitamin d deficiency may be a risk factor affecting the success of implant therapy due to its role in bone regeneration and remodeling during the osseointegration process. 

## Objectives

 to evaluate the current state of the literature regarding the relationship between low serum vitamin d levels and early failure of dental implants. 

## Material and Methods

 a literature search was con-ducted in the PubMed and Scopus databases using the keywords “vitamin d deficiency,” “early implant failure,” and “osseointegration,” limiting the search results to the period between 2016 and 2026. Studies assessing serum vitamin d levels and reporting implant failures within the first months were included. systematic reviews, studies not reporting implant-related data, and animal studies were excluded. 

## Results

 a total of 6 studies were included. half of them reported a higher failure rate in patients with low vitamin d levels, especially in cases of severe deficiency (<10 ng/ml). However, the other half did not find a statistically significant association. 

## Discussion

 the possible association in cases of severe deficiency could be explained by the role of vitamin d in regulating the inflammatory response and osseointegration. variability in the threshold defining vitamin d deficiency and in the definition of early failure makes comparisons difficult. 

## Conclusions

 at present, vitamin d deficiency cannot be considered a direct factor in early implant failure. More well-designed clinical studies with standardized criteria are required. 

# Abstract: 28 - XEROSTOMIA IN PERI- AND POSTMENOPAUSAL WOMEN: A MULTIMODAL THERAPEUTIC STRATEGY AND CLINICAL ASSESSMENT TOOL (XERO-MENO INDEX)

**Authors:** I.S. Gómez Pérez, J.M. Seoane Romero

**Affiliations:** *Private Dental Practice, Lugo, Spain, Email: igomeper@gmail.com *

## Introduction

 Xerostomia is a prevalent condition in peri- and postmenopausal women, associated with hormonal changes, aging, and polypharmacy. Many commonly prescribed medications in this population have xerostomic side effects, which further aggravate salivary hypofunction. In addi-tion, patients with a history of cancer, particularly those undergoing radiotherapy or certain sys-temic treatments, are at increased risk of salivary gland dysfunction. Reduced salivary flow dis-rupts oral homeostasis, increasing the risk of dental caries, periodontal disease, opportunistic infections, and functional impairments, with a significant impact on quality of life. 

## Objectives

 To evaluate the effectiveness of a multimodal therapeutic protocol and to assess the utility of the XERO-MENO index as a clinical evaluation tool in peri- and postmenopausal women with xeros-tomia. 

## Material and Methods

 A pilot study in progress was conducted in patients presenting sym-ptoms consistent with xerostomia. As an initial inclusion criterion and diagnostic baseline, the Saliva-Check Buffer kit (GC) was used, allowing the assessment of both unstimulated and stimulated saliva, as well as buffering capacity. Salivary hypofunction was defined as a stimulated Salivary flow rate <3.5 mL.The therapeutic protocol included the regular use of salivary substitute gels, xylitol-containing lozenges several times per day, and mechanical stimulation through controlled use of olive pits. For clinical evaluation, the XERO-MENO index (Xerostomia in Menopausal Women) was developed as a specific tool integrating patient-reported subjective variables and clinician-assessed objective parameters. The structure of the index and the variables assessed are presen-ted in Table 1, enabling standardized and reproducible pre- and post-treatment evaluation.

## Results

 Patients showed clinically relevant improvement in the perception of oral dryness, with increased comfort and reduction of functional symptoms such as difficulty swallowing and speaking.

# Abstract: 29 - INTEGRATION OF FACIAL SCANNING IN IMMEDIATE COMPLETE DENTURE REHABILITATION IN GERIATRIC PATIENTS: A CASE REPORT

**Authors:** E. Fernández Lagares, C. Pérez Arnau, J. Peláez Rico, M.J. Suárez Garcia

**Affiliations:** *Máster de Protesis bucofacial y oclusión, Facultad de Odontología, Universidad Complutense de Madrid. Email: elsafe01@ucm.es*

## Introduction

 Digitalization in dentistry has optimized clinical workflows through technologies such as intraoral scanners. More recently, facial scanning has emerged as a useful tool for integrating facial parameters into prosthetic design. In geriatric patients, where aesthetics and function are closely related to facial anatomy, three-dimensional records can improve clinical outcomes. 

## Objectives

 to describe a clinical case evaluating the utility of extraoral facial scanning as a complementary tool to intraoral scanning and to determine its advantages in geriatric patients. 

## Material and Methods

 A 70-year-old patient presented with residual roots. Following clinical, photographic, and radiographic evaluation, rehabilitation with an immediate maxillary complete denture was planned. Intraoral and extraoral facial scans were performed. Both datasets were integrated for prosthetic planning, considering midline, interpupillary line, and lip support. Subsequently, the indicated extractions were carried out and the immediate denture was placed. 

## Results

 This case demonstrates that the integration of intraoral and facial records facilitates proper occlusal plane orientation and accurate tooth positioning, improving lip support and facial harmony. A reduction on clinical adjustments was observed, along with satisfactory functional and aesthetic adaptation of the immediate denture. 

## Discussion

 The combined use of intraoral and facial scanning allows for more comprehensive treatment planning. Improvements in aesthetic planning, greater accuracy in tooth positioning, and a reduction in clinical adjustments are highlighted. In geriatric patients, facial scanning provides relevant information on lip support and facial dynamics, representing an added value compared to the exclusive use of intraoral scanning and conventional photography. Limitations include cost and the learning curve. 

## Conclusions

 Facial scanning is a useful tool in complete denture rehabilitation in geriatric patients. Its combination with intraoral scanning enables more precise, aesthetic, and personalized treatment, potentially improving clinical outcomes.

# Abstract: 30 - NARROW IMPLANTS AS AN ALTERNATIVE IN THE REHABILITATION OF ALVEOLAR RIDGES WITH HORIZONTAL BONE COMPROMISE IN ELDERLY PATIENTS

**Authors:** M.L. Castro Rodríguez, P. Gándara Vila, J.M. Somoza Martín, A. García García, A. Blanco Carrión, E.M. Otero Rey

**Affiliations:** *Master's in Oral Medicine, Oral Surgery, and Implantology at the University of Santiago de Compostela. Email: elsafe01@ucm.es*

## Objectives

 To evaluate the effectiveness of using narrow-diameter implants to rehabilitate dental arches with horizontal bone loss in elderly patients using implant-supported removable prostheses. 

## Study Design

 We reviewed the clinical records of 25 patients (17 women and 8 men) with an average age of 66.4 years who were treated by the same operator between February 2021 and March 2026 in our master's program for the placement of narrow implants. Various variables were analyzed, such as the O'Leary index, implant position in the arch, OHIPs (Oral Health Impact Profile), and complications of the performed treatment. 

## Results

 A total of 122 Straumann® Tissue Level Mini Implants with a diameter of 2.4 mm and lengths of 10 mm and 12 mm were placed. In the upper arch, 25% were placed at position 14, 15% at positions 11, 12, and 21, and 5% at positions 23 and 22, while in the lower arch, 24.2% were placed at position 33, 18% and 15% at positions 31 and 41, respectively. Regarding the OHIP, we compared the values of the questionnaire prior to surgery with the values once rehabilitated and observed that there is improvement in all patients. We had one complication in a patient who was on multiple medications with systemic diseases, where a granuloma developed, and four mini-implant losses; three spontaneous losses and one explantation. 

## Conclusions

 Narrow-diameter implants seem to be a good solution in patients who have a jawbone ridge with compromised bone level. The simple surgical protocol is minimally invasive, and the treatment significantly improves the quality of life of these patients.

# Abstract: 31 - NASOPALATINE CYST: A RETROSPECTIVE STUDY OF 63 CASES AND A REVIEW OF THE CLINICAL AND HISTOLOGICAL ASPECTS

**Authors:** R. Cáceres Pinto, A. Pérez Jardón, S. Baña, P. Cea, A. García García

**Affiliations:** *Universidad Santiago de Compostela Email: Rodrigo.caceres@mayor.cl*

## Introduction

 The nasopalatine duct cyst is the most common non-odontogenic cyst of the jaws, originating from the proliferation of epithelial remnants of the nasopalatine duct. It can be associated with factors such as trauma, infection, or irritation, although in some cases it appears spontaneously. Despite presenting relatively typical clinical and radiographic characteristics, its diagnosis is complex due to the lack of clinical symptoms, which can lead to diagnostic errors and unnecessary treatments. 

## Goals

 To describe the clinicopathological characteristics of 63 cases of nasopalatine cysts, as previously described in the literature. 

## Material and Methods

 Demographic and clinical data were collected, including age, sex, race, lesion location, symptoms, radiographic and histopathological characteristics, and treatment. The diagnosis was based on clinical evaluation, radiographic studies (periapical radiographs, panoramic radiographs, and CBCT), and histopathological confirmation. A literature review was also conducted using databases such as PubMed, Embase, and Scopus. 

## Results

 A total of 63 cases of nasopalatine cysts were evaluated, with a mean age of 47 years and no sex predilection. Twenty-one cases were asymptomatic, while 34 presented with symptoms such as pain and swelling. Radiographically, it appears as a well-defined radiolucent lesion located between the maxillary central incisors, within the nasopalatine canal. The clinicopathological and radiographic findings in the literature review were consistent with this study. 

## Conclusions

 The nasopalatine duct cyst is a common lesion that can easily be misdiagnosed without proper evaluation. It is essential to include it in the differential diagnosis of lesions in the anterior maxillary region. Accurate diagnosis requires a combination of clinical examination, radiographic studies including CBCT, and histopathological confirmation.

# Abstract: 32 - IMPACT OF INTERDISCIPLINARY INTERVENTIONS ON ORAL HYGIENE ACTIVITIES IN HOSPITALIZED OLDER ADULTS

**Authors:** J. Espinoza González, T. Medina Reyes

**Affiliations:** *National Institute of Geriatrics Chile Email: javiera.espinoza@utalca.cl*

## Introduction

 Functional decline among hospitalized older adults represents a critical challenge for healthcare systems, particularly regarding the preservation of basic activities of daily living such as oral hygiene. In geriatric inpatient settings, functional deterioration is frequently associated with acute illness superimposed on pre-existing multimorbidity, leading to reduced autonomy and increased care dependency. Within this framework, interdisciplinary care has been widely recognized as an essential approach to mitigate functional decline through coordinated, patient-centered interventions. 

## Objectives

 To describe the impact of interdisciplinary interventions on oral hygiene activities in hospitalized older adults. 

## Material and Methods

 Two inpatient cases were purposively selected based on their potential for functional variation in oral hygiene performance following intervention. Clinical characteristics were briefly described, and individualized interdisciplinary strategies were implemented, including assistive device adaptation and task-specific functional training. Immediate functional outcomes were assessed using case-tailored evaluation instruments. 

## Results

 Interventions included modifications for denture hygiene in a patient with neurological sequelae, and adaptive adjustments to oral hygiene tools for a patient with osteoarticular deformities, complemented by targeted training to enhance task execution. Both cases demonstrated improvements in oral hygiene outcomes and increased patient engagement in self-care activities. 

## Discussion

 Interdisciplinary approaches enable the development of individualized, function-oriented strategies that enhance autonomy in oral hygiene practices. Furthermore, they facilitate the identification of functional barriers and the implementation of adaptive solutions aligned with patients’ capacities. 

## Conclusions

 Interdisciplinary interventions incorporating functional adaptations appear to positively influence the efficiency and autonomy of oral hygiene practices in selected hospitalized older adults.

# Abstract: 33 - FACTORS RELATED TO DENTAL IMPLANTS’ FAILURE. STUDY ON 1308 IMPLANTS

**Authors:** M.N. Gómez Rivas, Á.O. Salgado Peralvo, P. Boñar Álvarez, M.S. Baña Souto, F. Seijas Naya, M. Pérez-Sayáns

**Affiliations:** *Unidad de Medicina Oral, Cirugía Oral e Implantología – Facultad de Odontología – Universidad de Santiago de Compostela. Email: marianatalia.gomez@rai.usc.es*

## Introduction

 Implants’ failure can be defined as its loss; this phenomenon’s disadvantages include its economical and biological cost, as well as the loss of faith from our patients. The objective of this study is to estimate the cumulative failure of 1308 implants and to identify related factors, either patient or surgery-dependant. 

## Material and Methods

 for this multicentric retrospective study, we extracted data from 6 private clinics between June 2006 and December 2022 and evaluated factors related to the patients, surgery and the implants themselves. For identifying failure-predictive factors, we used multivariate mixed Cox proportional hazards models and Lasso regularization. 

## Results and discussion

 1308 implants were inserted in 510 patients, obtaining a failure of 2,4% during the follow-up period of 38,31+-32,54 months. The used Cox models showed us 3 variables independently associated to implant failure: post-surgical infection, previous implant failure and heavy smoking habit (understood as consuming more than 20 cigarettes per day). 

## Conclusions

 with our study’s limitations, cumulative failure was low. However, it shows that it’s essential to strictly control our patients’ smoking habit, as well as the risk of infection, as implant replacement is shown as an independent risk factor for subsequent failure.

# Abstract: 34 - REGENERATION OF PERIODONTAL INTRABONY DEFECTS USING PLATELET‑RICH FIBRIN (PRF): A SYSTEMATIC REVIEW AND NETWORK META‑ANALYSIS

**Authors:** N. Matala Álvarez, F. França Vieira e Silva, C. Menendez, C. López, M. Cerra, J.M. Somoza

**Affiliations:** *Máster de Medicina Oral, Cirugía Oral e Implantologia USC Email: nasser_ab98@hotmail.com*

One of the most promising approaches to correct periodontal bone defects and achieve periodontal regeneration is platelet- rich fibrin (PRF). This systematic review and meta-analysis aimed to evaluate the regeneration of periodontal bone defects using PRF compared to other regenerative treatments. The data search and retrieval process followed the PRISMA guidelines. An electronic search of MEDLINE, Cochrane, and PubMed databases was performed, selecting exclusively randomized clinical trials where the following were measured: probing depth reduction (PD), clinical attachment level gain (CAL), and radiographic bone fill (RBF). Out of 284 selected articles, 32 were chosen based on inclusion criteria. The use of platelet- rich fibrin (PRF) + open flap debridement (OFD), PRF + metformin, PRF + platelet-rich plasma (PRP), and PRF + OFD/bone graft (BG) significantly reduced PD and improved CAL and RBF. However, the combination of PRF + BG, PRF + metformin, and PRF + STATINS reduced CAL. The intervention of PRF combined with different treatments such as metformin, OFD, PRP, BG, and STATINS has a significant impact on improving PD and CAL. The use of PRF significantly improved the regeneration of periodontal bone defects compared to other treatments.

# Abstract: 35 - USE OF TOPICAL VITAMIN E IN ORAL MUCOSITIS IN PATIENTS UNDERGOING ONCOLOGY TREATMENT. SCOPING-REVIEW

**Authors:** D. Rey Franco, P. Arestín Cea, I.B. Padro-Pena, C. Rodriguez Lopez, C. Menendez Beatriz, V. Sanmartin Barragáns

**Affiliations:** *Universidad de Santiago de Compostela. Email: dierf1611@gmail.com*

## Introduction

 Oral mucositis (OM) refers to lesions characterized by erythema and ulceration of the oral mucosa, frequently seen in cancer patients undergoing chemotherapy (CT) and/or radiotherapy (RT). Currently, there is no specific therapy supported by strong scientific evidence for the treatment of OM-related lesions. However, the literature suggests that antioxidants, such as vitamin E, could help prevent oxidative cell damage and limit tissue injury caused by free radicals, potentially reducing the severity of OM during cancer treatment. The aim of this review is to analyze the existing literature on the use of topical vitamin E and its effects on cancer therapy-induced OM lesions. 

## Material and Methods

 This review was conducted according to the PRISMA-ScR guidelines. A comprehensive search was performed in the Web of Science (WOS), PubMed, and Scopus databases. Keywords: "vitamin E, "oral mucositis," "chemotherapy," and "radiotherapy." Studies published between 1990 and 2025 were considered for inclusion. 

## Results

 167 articles were identified. Seven clinical trials were included in the review: four with pediatric populations and three with adults. Six of the studies reported favorable results with the use of vitamin E, including improved healing, reduced symptom severity, and shorter duration of bone marrow lesions. 

## Conclusions

 Topical vitamin E appears to offer beneficial effects in mitigating the severity and duration of bone marrow lesions in patients undergoing cancer treatment. However, there is currently no standardized protocol regarding dosage, application Methods, or treatment duration. Further clinical trials are warranted to establish clear guidelines for the use of topical vitamin E in this context.

# Abstract: 36 - RETINOIC ACID PROFILES IN PROLIFERATIVE VERRUCOUS VERSUS HOMOGENEOUS LEUKOPLAKIA: A PRELIMINARY NESTED CASE–CONTROL STUDYLajarín Gázquez J, Boñar Álvarez P, Seijas Naya F, Cerra González M, Barrero López C, Chamorro-Petronacci C

**Authors:** J. Lajarín Gázquez, P. Boñar Álvarez, F. Seijas Naya, M. Cerra González, C. Barrero López, C. Chamorro-Petronacci

**Affiliations:** *Faculty of Dentistry, University of Santiago de Compostela, and Vithas Vigo Hospital. Email: joselajaringazq@gmail.com*

## Introduction

 Oral leukoplakia and proliferative verrucous leukoplakia remain challenging entities due to the lack of reliable prognostic biomarkers. All-trans retinoic acid (atRA), a key modulator of epithelial differentiation and mucosal integrity, has been proposed as a potential biomarker. 

## Objectives

 The aim of this study was to quantify plasma retinoic acid levels in patients with oral leukoplakia and proliferative verrucous leukoplakia compared with healthy controls. 

## Material and Methods

 A cohort of 40 participants was recruited, including 10 patients with leukoplakia, 10 with proliferative verrucous leukoplakia, and 20 healthy controls. This nested case–control study was derived from previously characterized institutional databases of oral potentially malignant disorders. Statistical analyses were performed to assess differences between groups and associations with clinical outcomes. 

## Results

 Patients with oral leukoplakia exhibited significantly lower atRA levels compared to both proliferative verrucous leukoplakia patients and healthy controls. No significant differences were observed between proliferative verrucous leukoplakia and controls. No association was found between atRA levels and clinical variables or malignant transformation. 

## Discussion

 The reduction of atRA in oral leukoplakia suggests an early disruption in retinoid signaling, potentially involved in oral carcinogenesis. The absence of changes in proliferative verrucous leukoplakia supports etiopathogenic differences between both entities. The limited diagnostic performance of atRA indicates that its utility may be restricted to multimarker panels. 

## Conclusions

 Decreased atRA levels may represent a prognostic biomarker in oral leukoplakia, whereas its role in proliferative verrucous leukoplakia appears limited. Effect estimates were imprecise, and further studies are warranted.

# Abstract: 37 - MINIMALLY INVASIVE MANAGEMENT OF NON-CARIOUS CERVICAL LESIONS USING A BOPT APPROACH

**Authors:** V. Kornuta Krichfalovshiy, A. Pérez Barrionuevo, V. Rodríguez, C. Tobar Arribas, J. Peláez Rico, M.J. Suárez García

**Affiliations:** *Complutense University of Madrid. Email: veronicakornuta@gmail.com*

## Introduction

 Non-carious cervical lesions (NCCLs) are characterized by the loss of tooth structure in the cervical region without bacterial involvement. Their etiology is multifactorial, and they can lead to dentin hypersensitivity, aesthetic compromise, and periodontal changes. In recent years, the search for minimally invasive treatments has driven the development of conservative restorative approaches that allow for the preservation of tooth structure and the optimization of periodontal tissue stability. The BOPT (Biologically Oriented Preparation Technique), originally described in fixed prosthodontics to modulate the emergence profile and promote soft tissue adaptation, has begun to be applied to direct restorations using composite resins. 

## Material and Methods

 A 73-year-old patient presented to the Prosthodontics Master’s Program with multiple LCNCs, requesting a conservative therapeutic solution that would avoid the need for fixed indirect restorations. Following clinical evaluation and periodontal analysis, treatment was planned using direct restorations based on BOPT principles. The restorations were designed to create a convex cervical emergence profile. Follow-up clinical examinations were performed to evaluate the periodontal response. 

## Results

 The restorations demonstrated adequate aesthetic and functional integration. Clinically, proper adaptation of the gingival tissue to the new cervical profile, periodontal stability, and reduced dentin sensitivity were observed. 

## Discussion

 Several studies indicate that applying BOPT principles to teeth with non-carious cervical lesions restored with composite resin can induce favorable changes in gingival tissue volume and adaptation by modifying the emergence profile. Furthermore, longitudinal studies have demonstrated long-term periodontal stability, supporting its biological potential and utility as a conservative restorative approach. 

## Conclusions

 The application of BOPT principles in direct composite resin restorations may constitute a conservative therapeutic alternative for the management of non-carious cervical lesions, allowing for the restoration of cervical morphology and promoting a favorable periodontal response.

# Abstract: 38 - IMPORTANCE OF BLOOD PRESSURE MEASUREMENT IN THE DENTAL CLINIC. AGE AS A DECISIVE FACTOR

**Authors:** B. González Navarro, A. Matinyan, A. Oliveras-Serrano, A. El Kanfoud Ezzarraa, M. Mesquida, J. López-López

**Affiliations:** *Master of Medicine, Oral Surgery and Implantology, Faculty of Medicine and Health Sciences (Dentistry), University of Barcelona. Email: veronicakornuta@gmail.com*

## Introduction

 Hypertension is a major global public health problem and one of the leading modifiable risk factors for cardiovascular morbidity and mortality. Approximately 13% of people with hypertension are unaware of their condition. The aim of this study is to screen for hypertension in the dental office and analyze its relationship with oral pathology. 

## Material and Methods

 A systematic screening for oral health and hypertension is performed in patients attending the University of Barcelona Dental Hospital (HOUB). Hypertension was confirmed in patients with known hypertension, or when it was suspected based on blood pressure (BP) measurement in the dental office according to the European Society of Hypertension (ESH) guidelines. The presence of lingual varices (YES/NO) was also analyzed.

## Results and Discussion

 Results from the first 1,000 patients studied are presented. The mean age of the analyzed population was 45.4 ± 19 years, with a prevalence of women representing 54% (n = 540) compared to 460 men. Three hundred and one hypertensive patients (30.1%) were found, with a mean age of 53.61 ± 17 years. The mean age was higher in hypertensive women (56.7%) than in men (51.8%). Of the 301 patients, 175 were unaware of their condition (17.5% of the total and 58.1% of those with hypertension). A parameter of interest was the presence of lingual varices. Three hundred and one hundred nineteen of the 1,000 patients had lingual varices, and their presence was significantly higher in diagnosed hypertensive patients, undiagnosed hypertensive patients, and those over 65 years of age. 

## Conclusions

 The dentist plays an important role in the early diagnosis of hypertension. Sublingual varicose veins are associated with cardiovascular disease, and dentists can play a role in the prevention and early diagnosis of cardiovascular risk, especially in people over 65.

# Abstract: 39 - PLASMA CELL GINGIVITIS: SYSTEMATIC REVIEW AND CASE SERIES WITH EXPLORATORY PATTERN ANALYSIS

**Authors:** A. Medeiros Monzón, A. Blanco Carrión, P. Gándara Vila, A. Pérez Jardón, J.M. Suárez Peñaranda, M. Pérez Sayáns

**Affiliations:** *Department of Oral Medicine, Oral Surgery and Implantology (Med Oral Res), Faculty of Medicine and Dentistry, University of Santiago de Compostela, Spain. Email: alejandro.medeiros@rai.usc.es; alejandromedeirosmonzon@gmail.com*

## Introduction

 Plasma cell gingivitis (PCG) is a rare benign inflammatory entity of the oral mucosa, histopathologically characterized by a dense and diffuse infiltrate of polyclonal plasma cells in the subepithelial connective tissue. Its low prevalence, lack of standardized diagnostic criteria, and limited high-quality evidence make clinical management challenging. 

## Objectives

 To synthesize the available evidence on PCG through a systematic review and to describe the clinical, histopathological, and therapeutic profiles of a retrospective institutional case series. 

## Material and Methods

 A systematic review was conducted following PRISMA 2020 guidelines, searching PubMed, Scopus, and Web of Science without date restrictions. Additionally, 12 patients diagnosed with PCG were included, confirmed by histopathological and immunohistochemical analysis (CD3, CD20, κ/λ chains) at the Oral Medicine, Oral Surgery and Implantology Unit of USC. Statistical analysis included descriptive statistics and Kaplan–Meier survival curves (SPSS v26.0.1). 

## Results

 The systematic review included 11 studies. The institutional series had a mean age of 74.2 ± 16.6 years (8M/4F), with poor oral hygiene in 67% of cases. The most frequent treatment was 0.5% triamcinolone acetonide. The complete clinical resolution rate was 75% (9/12), with a median time to resolution of 30.1 weeks (IQR: 21.3–69.7). Multisite involvement was associated with a longer clinical course. Histopathologically, 100% of cases showed epithelial hyperplasia and spongiosis, with CD3 predominance in 83.3%. 

## Conclusions

 A definitive diagnosis of PCG requires histopathological and immunohistochemical confirmation. Topical corticosteroids are the most effective first-line treatment, achieving a 75% resolution rate. Multisite involvement may represent a prognostic factor for prolonged disease course. Multicenter prospective studies are needed to establish standardized management guidelines.

# Abstract: 40 - GENDER REPRESENTATION IN DENTAL SCIENTIFIC SOCIETIES: A COMPARATIVE ANALYSIS OF SEGER

**Authors:** E. Ibáñez Prieto, N. Martínez Rodriguez, J. Santos Marino, C.M. Madrigal Martínez Pereda, C. Barona Dorado

**Affiliations:** *Universidad Complutense de Madrid Email: eibane02@ucm.es*

## Introduction

 The feminisation of the Spanish dental profession is now well established. However, the representation of this female majority on the governing bodies of scientific societies affiliated to the Spanish Dental Council is progressing at an uneven pace, a phenomenon known as the ‘glass ceiling’.

## Objectives

 To analyse the representation of women on the executive committees of societies affiliated to the Spanish Dental Council, specifically comparing SEGER with the group of societies analysed up to December 2025. 

## Material and Methods

 A descriptive cross-sectional study involving a literature review of 15 scientific societies (51.7% of those affiliated). A total of 1,496 positions were analysed, classified by gender, type of position and term of office. 

## Results

 Overall, the historical proportion of women stands at 19.18%. However, there are differences between societies. Whilst the current average proportion of women in leadership positions stands at 38.46% for 2025, SEGER shows a notable trend: with 39.73% of women in its historical total and reaching 54.55% on the current board of directors. These figures contrast with other societies where both historical and current figures remain below average. 

## Discussion

 SEGER, alongside societies such as SESPO (51.9%), leads the way in gender parity. In contrast, the overall results show that, although middle management positions (committee members) are becoming more balanced, the presidency remains predominantly male (90.64%), a gap that SEGER has begun to narrow more rapidly than the average. 

## Conclusions

 SEGER stands out as a benchmark for female representation among the societies affiliated to the Council of Dentists, exceeding the overall average of the societies analysed. Despite the positive trend towards equality by 2025, there remains a need to promote women’s access to positions of highest responsibility to reflect the socio-demographic reality of the profession.

# Abstract: 41 - AVAILABLE TREATMENTS FOR XEROSTOMIA: AN EVIDENCE-BASED THERAPEUTIC APPROACH

**Authors:** V. Schiavo Di Flaviano, C. Martín Carreras-Presas, J. López López, E. Jane Salas

**Affiliations:** *Master’s Degree in Oral Medicine, Surgery and Implant Dentistry. University of Barcelona Email: veroverosd@gmail.com*

## Introduction

 Dry mouth is a common clinical problem with significant functional and biological impact. The absence of a curative treatment requires a palliative and stepwise approach based on an understanding of the mechanisms of action of the available therapies. The aim of this study is to describe and classify the treatments available for xerostomia according to their mechanisms of action and level of scientific evidence. 

## Material and Methods

 A structured literature search was conducted in the PubMed, Scopus, and Web of Science databases, focusing on the different therapeutic alternatives studied for the treatment of xerostomia. Studies published within the last 10 years were included. 

## Results

 Pharmacological treatments, particularly cholinergic agonists such as pilocarpine, demonstrated the greatest clinical efficacy, mainly in patients with residual glandular function. Non-pharmacological topical treatments, including saliva substitutes and mechanical stimulants, were shown to improve subjective symptoms without increasing salivary flow. Alternative therapies, such as electrostimulation and acupuncture, showed favorable outcomes in selected cases, although the supporting evidence remains limited. Experimental treatments, such as mesenchymal stem cell therapy, are still in early stages of development. 

## Discussion

 The treatment of xerostomia requires a personalized approach, conditioned by etiology and residual glandular function. Pharmacological therapies are more effective in early stages, whereas topical treatments mainly provide symptomatic relief. 

## Conclusions

 The available evidence on xerostomia treatments is variable and presents a high degree of Methodsological heterogeneity. Further controlled clinical studies with homogeneous diagnostic and evaluation criteria are needed to allow reliable comparisons and strengthen current therapeutic recommendations.

# Abstract: 42 - AESTHETIC ANALYSIS OF PERI-IMPLANT SOFT TISSUES IN OLDER ADULT PATIENTS AFTER POST-EXTRACTION IMPLANTS AND IMMEDIATE ESTHETICS

**Authors:** M. Andrés Veiga, D. Hurtado Celotti, E. Cañaveral Cavero, L. Sánchez Labrador, T. Beca Campoy, P.L. Ruíz Sáenz, M.H. Rey Martínez, N. Martínez Rodríguez

**Affiliations:** *Alfonso X University. Email: meris2@msn.com*

## Objectives

 To evaluate the cognitive-functional profile and oral health status of older adults (65-79 years) and the elderly (≥80 years) in the Community of Madrid, analyzing significant differences between sexes. 

## Material and Methods

 A cross-sectional multicenter epidemiological study (n=615) was conducted across three dental centers. Functional capacity was assessed using the Katz and Lawton & Brody Indices. A clinical examination was performed to evaluate oral pathology, hygiene, and treatment needs. Statistical analysis included the Mann-Whitney, Kruskal Wallis, and Chi-square tests (p<0.05). 

## Results

 The sample was divided into the "third age" (79%) and "fourth age" (21%). It was observed that dependency increases with age, correlating with greater medical pathology and a higher daily intake of tablets. When comparing by sex, women show greater longevity but poorer general health conditions, manifesting more chronic disorders and a higher daily consumption of drugs. Regarding functionality, men showed greater independence in basic activities, while women were more independent in instrumental activities. In terms of oral health, men present a significantly worse status, with a higher presence of caries, periodontal disease, root remains, unfavorable oral hygiene, and a greater need for dental assistance compared to women. 

## Conclusions

 Marked gender differences exist in aging. While women report poorer general health and greater dependency in basic activities, men present more deteriorated oral health. The degree of functional dependency, especially in instrumental activities, acts as a critical determinant of oral health status and dental treatment needs in both sexes.

# Abstract: 43 - ORAL HEALTH AND COGNITIVE-FUNCTIONAL PROFILE IN AGING: A MULTICENTER STUDY WITH A GENDER PERSPECTIVE

**Authors:** E. Cañaveral Cavero, L. Sánchez Labrador, T. Beca Campoy, P.L. Ruiz Sáenz, M.H. Rey Martínez, N. Martínez Rodríguez

**Affiliations:** *Complutense University of Madrid. Email: esthercanaveral@hotmail.com*

## Objectives

 To evaluate the cognitive-functional profile and oral health status of older adults (65-79 years) and the elderly (≥80 years) in the Community of Madrid, analyzing significant differences between sexes. Methods: A cross-sectional multicenter epidemiological study (n=615) was conducted across three dental centers. Functional capacity was assessed using the Katz and Lawton & Brody Indices. A clinical examination was performed to evaluate oral pathology, hygiene, and treatment needs. Statistical analysis included the Mann-Whitney, Kruskal Wallis, and Chi-square tests (p<0.05). 

## Results

 The sample was divided into the "third age" (79%) and "fourth age" (21%). It was observed that dependency increases with age, correlating with greater medical pathology and a higher daily intake of tablets. When comparing by sex, women show greater longevity but poorer general health conditions, manifesting more chronic disorders and a higher daily consumption of drugs. Regarding functionality, men showed greater independence in basic activities, while women were more independent in instrumental activities. In terms of oral health, men present a significantly worse status, with a higher presence of caries, periodontal disease, root remains, unfavorable oral hygiene, and a greater need for dental assistance compared to women. 

## Conclusions

 Marked gender differences exist in aging. While women report poorer general health and greater dependency in basic activities, men present more deteriorated oral health. The degree of functional dependency, especially in instrumental activities, acts as a critical determinant of oral health status and dental treatment needs in both sexes.

# Abstract: 44 - ROOT RESECTION: AVOIDING DENTAL IMPLANT TREATMENT IN THE AGING ADULT PATIENT. A CASE REPORT WITH FIVE YEAR FOLLOW-UP AND LITERATURE REVIEW

**Authors:** L. Sánchez-Labrador, T. Beca Campoy, P.L. Ruiz Sáenz, M.H. Rey Martínez, N. Martínez Rodríguez, M. Andrés Veiga

**Affiliations:** *Faculty of Dentistry. Complutense University of Madrid. Email: luissanc@ucm.es*

## Introduction

 Root amputation is a surgical technique performed in multi-rooted teeth, sectioning and deleting one roots, maintaining intact the coronal portion. 

## Objectives

 to analyze available scientific literature, and presenting a clinical report where this technique is evaluated with five years of follow-up. 

## Clinical case description

 a sixty-year-old male patient is presented, who received a root amputation of mesio-buccal root of the upper left first molar, due to a vertical fracture, with promising clinical and radiological evolution, 5 years after the procedure. 

## Results

 root amputation is a technique with survival rates higher than 80% in the recent literature, with better results in maxilla than in mandible. This surgical technique allows maintaining a good masticatory function, with adequate periodontal and prosthodontic controls. 

## Discussion

 although dental implants are actually the gold standard for restoring absent teeth, there is a serious problem about peri-implant diseases they can be affected by, so alternative treatments such as root amputation are nowadays popular because it is a less invasive treatment, with lower economical costs for patients and with a good acceptation rate. In this scenario, it should be considered as an option, before performing an extraction and placing a dental implant. 

## Conclusions

 survival rate when root amputation is performed is lower in comparison to dental implants survival rate, but with a proper selection of the patient, it has good results and it is possible dental implant placement if it fails in a near future.

# Abstract: 45 - LONG-TERM-INCIDENCE-AND-DETERMINANTS-OF-SECONDARY-ORAL-CANDIDIASIS-IN-PATIENTS-WITH-ORAL-LICHEN-PLANUS-A-RETROSPECTIVE-COHORT-STUDY

**Authors:** A. Díaz Rodríguez, A.I. Lorenzo Pouso, P. Gándara Vila, J.M. Somoza Martín, A. Blanco Carrión, E.M. Otero Rey

**Affiliations:** *Facultad de Odontología. Universidad de Santiago de Compostela Email: albadiaz019@gmail.com*

## Introduction

 Oral lichen planus (OLP) often requires prolonged topical corticosteroid therapy, and secondary oral candidiasis is a recognised complication. Long-term incidence and independent predictors, however, are not fully defined. 

## Objectives

 This study aims to estimate the prevalence, timing, and determinants of microbiologically confirmed oral candidiasis in patients with OLP receiving extended topical corticosteroid treatment. 

## Material and Methods

 A retrospective cohort of 390 adults diagnosed with OLP (1997–2025) was reviewed. Demographic, behavioural, clinical, and therapeutic variables were collected. 

## Results

 Oral candidiasis developed in 85 patients (21.8%), with 68.2% of cases occurring after initiating topical corticosteroids (median time to onset: 4 months). Patients with candidiasis had more frequently erosive OLP (18.8% vs. 8.5%, p=0.009), poorer oral hygiene (23.5% vs. 11.5% poor, p=0.006), and removable partial dentures (35.3% vs. 19.7%, p=0.002). Independent predictors included topical corticosteroid use (aOR 4.2; 95% CI 1.9–9.6), poor oral hygiene (aOR 2.4; 95% CI 1.2–5.1), and presence of removable partial dentures (aOR 2.0; 95% CI 1.1–3.5). Alcohol consumption showed a marginal association (aOR 1.6; 95% CI 0.9–2.7). Age, sex, smoking, and other comorbidities showed no significant association. 

## Conclusions

 Secondary oral candidiasis is a frequent and early complication in OLP patients treated with topical corticosteroids, particularly those with erosive or severe disease, poor oral hygiene, or removable partial dentures. The early onset (median 4 months) and clear risk gradient support the implementation of risk-stratified follow-up during the initial months of therapy. Routine antifungal prophylaxis is not justified based on these findings.

# Abstract: 46 - MAXILLARY SINUS AUGMENTATION USING AUTOLOGOUS TOOTH-DERIVED GRAFT IN AN ELDERLY PATIENT: A CLINICAL CASE WITH 1-YEAR FOLLOW-UP AND LITERATURE REVIEW

**Authors:** T. Beca Campoy, P.L. Ruiz Sáenz, M.H. Rey Martínez, N. Martinez Rodriguez, M. Andrés Veiga, D. Hurtado Celotti

**Affiliations:** *Faculty of Dentistry. Complutense University of Madrid. Email: doctorbeca@gmail.com*

## Introduction

 Maxillary sinus floor elevation is a technique that enables rehabilitation of the atrophic posterior maxilla. Autologous dental graft (IAD from the Spanish injerto autólogo dental) has emerged as an alternative autologous biomaterial with favorable clinical, radiographic, histological, and histomorphometric outcomes. 

## Objectives

 to describe the use of IAD in maxillary sinus floor elevation in an older adult patient through a clinical case, and to review the available literature. 

## Material and Methods

 67-year-old male with a pacemaker, tooth-supported bridge from 2.4 to 2.8, fistula at 2.4–2.5, and absence of 2.6–2.7. Teeth 2.4, 2.5, and 2.8 were extracted, and sinus floor elevation was performed using a lateral window approach with Sinus Master Kit III (1200 rpm, external irrigation, bur with a 2 mm active part). IAD from 2.4, 2.5, and 2.8 was processed with Tooth Transformer® and combined with L-PRF. L-PRF membranes were placed over the Schneiderian membrane. The lateral access was covered with a Lyoplant® collagen membrane fixed with 2 Master Pin® pins and Supramid® 4/0 sutures. Re-entry at 5 months: 3 Naturactis® implants and trephine biopsies 3×7 mm at 2.6 and 2.7 for histomorphometric analysis. 

## Results

 initial height was 1.4 mm (2.6) and 3.2 mm (2.7). At 5 months: 9.4 mm and 11.3 mm, with approximate gains of +8.0 and +8.1 mm, allowing placement of 3 implants. Histomorphometry: vital bone 22.45–26.61%; residual material 6.11–7.91%; connective tissue 67.28–69.64%. Favorable clinical and radiographic follow-up at 12 months. 

## Discussion

 bone gain and re-entry at 5 months are consistent with the published evidence on IAD in maxillary sinus floor elevation. Histomorphometry showing vital bone and low residual material is consistent with biopsies describing remodeling. 

## Conclusions

 IAD provided sufficient regeneration for delayed implant placement and demonstrated vital bone with low residual material at 5 months, supporting its use as an autologous alternative. Limitations related to heterogeneity and the lack of controlled trials indicate that further evidence is needed.

# Abstract: 47 - THE PRESENCE OF BIOETHICS IN GERODONTOLOGY STUDIES IN SPANISH DENTISTRY. WHAT MIGHT HAPPEN WITH THE ARRIVAL OF AI? A LITERATURE REVIEW

**Authors:** M. Moradas Estrada, B. Alvarez Lopez

**Affiliations:** *Clinica Dental Tapia Drs Álvarez & Moradas. Email: moradasmarcos@uniovi.es*

Often, a significant portion of studies, publications, and research focus on dental materials, equipment, and techniques, perhaps dedicating insufficient time to exploring ethics in dental practice. The arrival of AI in teaching and dental practice may represent either a problem or an advantage in this often unknown, and sometimes neglected, field of our profession. Therefore, we have undertaken this literature review with the following objectives: To determine the number of ECTS credits dedicated to content and/or competencies related to ethics or bioethics in undergraduate Dentistry studies at Spanish public universities, specifically in gerodon-tology/geriatrics courses. To ascertain the existence of specific courses on gerodontology and ethics/bioethics. To determine the potential role of AI in patient management, care, and assistance from a teaching perspective. With the inherent limitations of this study, the following was observed: Only between 5.2% and 13% of the total credits in any of the observed competencies were dedicated to aspects of ethics or bioethics; The existence of specific courses was minimal, many without instruction or having disappeared, at most (in 3 cases) offered as an elective in the third year; There is a lack of real implementation of AI in curricula and it remains largely unknown in discussions about AI.

# Abstract: 48 - EVALUATION OF MELATONIN AS A POTENTIAL PREOPERATIVE ANXIOLYTIC IN ELDERLY PATIENTS UNDERGOING IMPLANT SURGERY: A PILOT STUDY

**Authors:** N. Martínez Rodríguez, M. Andrés Veiga, D. Hurtado Celotti, E. Cañaveral Cavero, L. Sánchez Labrador, T. Beca Campoy

**Affiliations:** *Universidad Complutense de Madrid. Facultad de Odontología. Email: nataliamartinez@ucm.es*

## Introduction

 Preoperative dental anxiety is an adaptive emotional response to scheduled surgical procedures that typically peaks on the day of surgery. 

## Objectives

 The aims were to evaluate the efficacy of orally administered melatonin in reducing anxiety levels and postoperative pain in elderly adult patients undergoing implant surgery. 

## Material and Methods

 A prospective, single-blind, multicenter, split-mouth longitudinal clinical study was conducted on patients who were candidates for implant-based rehabilitation and who had preoperative anxiety scores greater than 9 on the Modified Dental Anxiety Scale. A total of 20 patients were selected and received a total of 40 implants. Anxiety levels were assessed using biological parameters such as blood pressure and heart rate, as well as the Visual Analog Scale, both preoperatively and postoperatively. 

## Results

 The mean age of the patients was 75.4 years. No surgical complications or undesirable side effects were reported. Statistically significant differences were found in favor of melatonin compared to placebo. 

## Discussion

 Comorbidities and medication use are common among older adults, so situations such as surgeries that induce anxiety could lead to a disruption of various biological parameters. Moreover, melatonin has a safer side-effect profile compared to more potent anxiolytics such as midazolam, which is associated with drowsiness and impaired cognitive function. 

## Conclusions

 Melatonin may be an alternative for managing preoperative anxiety in the surgical setting.

# Abstract: 49 - IMPLANT RETREATMENT USING GUIDED PLANNING

**Authors:** S. Ruiz Garcia, I. Filali Baba Louartiti, P. ontevedra Gómez, J. Peláez Rico, M.J. Suárez García

**Affiliations:** *Máster de Prótesis de la Universidad Complutense de Madrid. Email: silrui06@ucm.es*

## Introduction and Objectives

 Inadequate prosthetic planning may compromise the success of the treatment. Therefore, proper implant distribution, together with guided planning, increases predictability and contributes to achieving satisfactory outcomes. 

## Material and Methods

 This report presents the case of a 78-year-old female patient, classified as ASA II according to the ASA Physical Status Classification System, who attended the clinic of the Master’s Program in Maxillofacial Prosthodontics and Occlusion at the Complutense University of Madrid for rehabilitation of the mandibular arch using implants. Regarding her dental history, the patient reported previously wearing a hybrid prosthesis supported by six implants. After ten years of function, all implants were removed due to failure secondary to peri-implantitis. Vertical bone regeneration procedures were subsequently performed to allow placement of the current implants. Clinical examination revealed a skeletal Class II relationship. In the maxillary arch, the patient had been wearing, for more than ten years, a tooth-supported fixed prosthesis (PF1) made of metal-ceramic. This prosthesis was in good condition, hygienic, and although its esthetics could be improved, it was considered clinically acceptable. The treatment plan consisted of a fixed implant-supported rehabilitation following the All-on-Four concept, using a titanium framework and composite teeth. Guided surgery was planned for the placement of three additional implants, along with the fabrication of a complete provisional prosthesis to be subsequently converted into an immediate prosthesis. 

## Results

 The implant-supported fixed rehabilitation allowed restoration of the three fundamental objectives of oral rehabilitation: health, function, and esthetics. Furthermore, the retreatment was designed following hygienic criteria, with the aim of minimizing the risk of future peri-implant diseases. 

## Conclusions

 The establishment of an individualized treatment plan is essential for therapeutic success. Accurate diagnosis, proper planning, and guided execution of treatment facilitate appropriate reevaluation and potential modification of the clinical approach.

# Abstract: 50 - DENTAL CARE FOR GERIATRIC PATIENTS WITH COGNITIVE IMPAIRMENT IN A HOSPITAL CARE UNIT

**Authors:** P.L. Ruiz Saenz, M.H. Rey Martínez, N. Martínez Rodríguez, M. Andrés Veiga, D. Hurtado Celotti, E. Cañaveral Cavero

**Affiliations:** *Central Red Cross Hospital of Madrid. Email: drruizsaenz@gmail.com*

## Introduction

 The Royal Order of March 21, 1901, marks the beginning of university studies in Dentistry in Spain, just seventeen years before the inauguration of the Red Cross Hospital. At this point, we can say that modern dentistry began, and it continues to evolve in the way we care for the most vulnerable patients. 

## Objectives

 To describe the evolution of the patient profile served by a geriatric dentistry unit providing home and residential care, as well as its clinical activity from 2023 to 2025. 

## Material and Methods

 Data from the activity were recorded in aggregate form, and sociodemographic, clinical, functional, and mental variables (using various scales) were collected. Waiting time to the first visit, duration of the intervention, patient origin, and reason for consultation were also recorded. A descriptive analysis was performed using SPSS® version 23. 

## Results and Discussion

 Three thousand six hundred and seventy-nine patients were seen in our unit (January 1, 2023, to December 31, 2025). A progressive increase in age and geriatric syndromes was observed. Patients presented greater functional and mental decline, with an increase in patients living alone and requiring private assistance. The duration of the intervention decreased, and oral health status was assessed. 

## Conclusions

 Over the last two decades, geriatric patients have become increasingly vulnerable, exhibiting more geriatric syndromes, greater functional and mental dependence, significant social fragility, and numerous oral health disorders, particularly among institutionalized patients. Further research is needed to address the role of these support units in managing complex care within the community, as well as their coordination with other healthcare professionals.

# Abstract: 51 - AESTHETICS ALSO MATTER IN THE ELDERLY PATIENT

**Authors:** M. González Álvarez, A.M. Seyed, V. Rodríguez, M.J. Suárez

**Affiliations:** *Universidad Complutense de Madrid. Email: marina52@ucm.es*

## Introduction and Objectives

The increase in life expectancy and improvements in quality of life have changed patient expectations, including those of older individuals, who are increasingly demanding dental treatments focused not only on function but also on aesthetics. Tooth discoloration secondary to endodontic treatments is a common finding in clinical practice and can significantly compromise the appearance of the anterior region. Masking these darkened substrates represents a clinical challenge in which factors such as substrate color, cementation material, and the optical properties of the restorative material play a decisive role in the final outcome. The aim of this study is to describe the clinical protocol for rehabilitation using crowns in the maxillary anterior sector, focused on masking discoloration in a patient with high aesthetic demands. 

## Material and Methods

 A patient with lithium disilicate crowns in the maxillary anterior sector presented to the clinic requesting an aesthetic improvement due to the darkening of two upper incisors. Retreatment of teeth 1.1 and 1.2 was performed, including stump re-preparation following the BOPT technique. Provisional crowns were placed, and after one month, digital impressions were taken by scanning the preparations as well as the provisionals both intra- and extraorally. Definitive monolithic zirconia crowns were then fabricated. 

## Results

 The selected material, monolithic zirconia with feldspathic cut-back, allowed masking of the dark color of the underlying stumps, achieving an aesthetic result well integrated with the adjacent teeth. 

## Conclusions

 Masking the color of endodontically treated teeth is an increasingly common challenge due to high patient aesthetic demands. The underlying tooth structure, the cementation agent, and especially the restorative material have a significant impact on the final appearance of the restoration.

# Abstract: 52 - FULL-MOUTH REHABILITATION OF SEVERE TOOTH WEAR AND CLASS III MALOCCLUSION USING ZIRCONIA RESTORATIONS: A CLINICAL CASE-BASED APPROACH

**Authors:** J. Tatari Rajjoub, M.F. Andrino, M.P. Estefan, C. Tobar Arribas, J. Peláez Rico, M.J. Suarez García

**Affiliations:** *Máster de Prótesis Bucofacial, Universidad Complutense de Madrid Email: marina52@ucm.es*

## Introduction and Objectives

Severe tooth wear and Class III malocclusion compromise masticatory function and facial esthetics, representing a significant clinical challenge. The aim of this work is to present a clinical protocol for full-mouth rehabilitation that combines controlled increase of vertical dimension, occlusion correction, the BOPT technique, and the use of zirconia restorations, evaluating patient satisfaction and clinical outcomes. 

## Material and Methods

 A 73-year-old female patient attended the Master's program in Prosthodontics at UCM for the rehabilitation of six implants. Intraoral examination revealed Class III malocclusion, severe tooth wear, and altered vertical dimension. A mock-up was performed to validate the vertical dimension and occlusal scheme. The BOPT technique was applied in the maxillary arch to optimize the emergence profile and restore soft tissue. The implants were rehabilitated with screw-retained crowns on teeth 14, 16, 26, 35, 36 and 46. Zirconia was used as a restorative material. 

## Results

 Case management included determination of the vertical dimension and a mock-up to evaluate the increase and esthetics before definitive preparation. The BOPT technique optimized the emergence profile. Prosthetic rehabilitation stabilized the occlusion, restored masticatory function and esthetics, achieving patient satisfaction. 

## Discussion

 Metal-ceramic restorations have traditionally been the gold standard, but today zirconia is increasingly used. In this case, zirconia was selected for its mechanical strength, biocompatibility, lower plaque accumulation, better periodontal integration, and superior esthetics, which is especially important when rehabilitating anterior teeth, allowing for predictable restorations. 

## Conclusions

 Accurate diagnosis and adherence to an organized clinical protocol are essential for predictable full-mouth rehabilitation. Integration of techniques such as BOPT and zirconia restorations allows restoration of function and esthetics, ensuring satisfactory outcomes.

# Abstract: 53 - FROM FUNCTIONAL LOSS TO OCCLUSAL BALANCE: PROSTHODONTIC APPROACH OF A CASE WITH REDUCED VERTICAL DIMENSION

**Authors:** L. Minguito Alaminos, M. Burgoa Urreta, C. Tobar Arribas, J. Peláez Rico, M.J. Suárez García

**Affiliations:** *Master’s Degree in Oral Prosthodontics and Occlusion, Complutense University of Madrid. Email: luchi.minguito@gmail.com*

## Introduction

 Oral rehabilitation of geriatric patients with advanced dental deterioration and loss of vertical dimension represents a prosthodontic challenge, requiring precise diagnosis and occlusal planning to restore health, function, and esthetics. 

## Objectives

 To describe the prosthodontic approach in a geriatric patient with reduced vertical dimension through a full fixed upper and lower rehabilitation. 

## Material and Methods

 A 66-year-old male, ASA II, attended the Master’s Degree Clinic in Oral Prosthodontics and Occlusion for complete upper and lower oral rehabilitation after prolonged neglect of dental care. Examination revealed root remnants, endodontically treated teeth with root caries, and generalized wear associated with reduced vertical dimension. After comprehensive diagnosis and diagnostic wax-up, a total oral rehabilitation was planned using combined tooth-supported and implant-supported fixed prostheses in both arches. Treatment was staged: elimination of infectious foci, extractions, implant placement, provisionalization, and final definitive rehabilitation. 

## Results

 Vertical dimension and stable occlusion were restored, with proper anterior guidance and load distribution, recovering masticatory function and esthetics. The patient adapted well to the new occlusal situation without associated symptoms. 

## Discussion

 In cases of severe wear, accurate determination of vertical dimension and a provisionalization phase are essential to restore occlusion. Combined tooth-supported and implant-supported fixed prostheses offer a predictable solution in complex rehabilitations. 

## Conclusions

 Accurate diagnosis and occlusal planning are fundamental in cases of reduced vertical dimension to restore function, health, and esthetics.

# Abstract: 54 - GERODONTOLOGY IN THE SPANISH DENTAL CURRICULUM: AN ANALYSIS OF UNIVERSITY TEACHING GUIDES

**Authors:** H. Benjilali, M. Mouhib, A. García Sánchez, N. Martínez Rodríguez, C. Gutiérrez Bonet, C. Barona Dorado

**Affiliations:** *Complutense University of Madrid Email: hindbenj@ucm.es*

## Introduction

 Population aging and the increase in life expectancy have modified the profile of dental patients, leading to a greater need for specific training in the management of elderly patients. In this context, gerodontology has gained increasing relevance within university dental education. 

## Objectives

 To analyze the presence and characteristics of gerodontology teaching in the curricula of the Degree in Dentistry across Spanish universities. 

## Material and Methods

 A descriptive observational study was conducted based on the analysis of the teaching guides for the Degree in Dentistry from Spanish universities with accessible information on their institutional websites. Both public and private universities were included. Subjects with content related to gerodontology, geriatric dentistry, or dental care for the elderly were identified. For each subject, variables such as name, academic year, number of ECTS credits, mandatory or elective status, and teaching approach (theoretical, clinical, or mixed) were recorded. A descriptive analysis was performed using frequencies and percentages. 

## Results

 Thirty-two dental schools were analyzed, and a specific gerodontology subject was identified in 10 universities. In most faculties, it is taught in the advanced years of the degree and features a predominantly mixed teaching approach. Variability was observed between public and private universities regarding teaching load and its status as a mandatory or elective course. 

## Discussion

 Training in gerodontology is present in numerous Spanish dental schools, although there is significant heterogeneity in its curricular integration.

## Conclusions

 It is necessary to continue developing university training in dental care for the elderly patient to meet the evolving needs of the population.

# Abstract: 55 - PROSTHETIC MANAGEMENT IN GERIATRIC PATIENTS WITH SKELETAL CLASS III, A CASE REPORT

**Authors:** M. Burgoa Urreta, L. Minguito Alaminos, C. Tobar Arribas, J. Peláez Rico, M.J. Suárez García

**Affiliations:** *Master’s Program in Maxillofacial Prosthetics and Occlusion, Complutense University of Madrid (UCM) Email: mariaburgoa00@gmail.com*

## Introduction

 In young patients with skeletal Class III, treatments such as orthopedics, orthodontics, or maxillofacial surgery are commonly used to correct skeletal discrepancies. However, these alternatives are not considered in geriatric patients, in whom systemic, bone, and functional conditions require tailored prosthetic solutions. 

## Objectives

 To restore function and comfort in a geriatric patient with a highly unfavorable intermaxillary discrepancy due to skeletal Class III, aggravated by resorption associated with edentulism. 

## Material and Methods

 A 73-year-old patient attended the Master’s Program in Maxillofacial Prosthetics and Occlusion at the Complutense University of Madrid with a maxillary overdenture supported by four locators, experiencing significant discomfort due to the loss of one implant. In the mandibular arch, the anterior sector was preserved, and the patient wore a removable partial denture. After case evaluation, the treatment plan included removal of the maxillary implants, extraction of the mandibular teeth, and fabrication of provisional complete dentures, followed by a full fixed implant-supported rehabilitation. 

## Results

 After performing prosthetically guided surgery for implant placement, an implant-supported overdenture on a milled bar with three locator attachments was fabricated for the maxillary arch. For the mandibular arch, an All-on-Four approach was initially planned; however, due to surgical complications, an implant-retained overdenture supported by three locators was performed instead. 

## Discussion

 In patients with severe bone resorption, prosthetic height greater than 15 mm throughout the arch, unfavorable maxillomandibular relationship, and compromised hygiene, implant-supported overdentures are the indicated therapeutic option. Additionally, they allow restoration of lip support and avoid excessive palatal coverage or overly long teeth in patients with high smile lines.

## Conclusions

 Implant-supported overdentures are an effective option in complex geriatric patients, enabling restoration of function and aesthetics with high levels of satisfaction and adaptation.

# Abstract: 01 - REVIEW OF THE MANAGEMENT OF THE OSTEOPOROTIC PATIENT: A CASE STUDY OF AN ELDERLY ADULT

**Authors:** E. Ibáñez Prieto, A. Flores, P. Ferrera Castellano, A. Bowen Varela, S. Bazal Bonelli, C.M. Madrigal Martínez Pereda

**Affiliations:** *Postgrado de especialización en Cirugía Bucal e Implantología UCM. Email: eibane02@ucm.es*

## Introduction

 Medication-related osteonecrosis of the jaw (MRONJ) is a rare complication associated with the treatment of osteoporosis. In patients receiving oral bisphosphonates (BF), the baseline risk is 0.1%, rising to 0.5% following surgical procedures, according to some authors. The presence of comorbidities such as diabetes mellitus exacerbates this risk by compromising microvascularisation and the immune response. 

## Objectives

 To update clinical guidelines for patients on long-term oral BF therapy and to evaluate the efficacy of preventive protocols in oral rehabilitation. 

## Material and Methods

 Protocols from leading bodies in Spain (SESPO, General Council of Dentists) were reviewed, as well as scientific literature from the last 10 years. Risk variables such as duration of exposure, drug potency and other systemic factors were analysed. 

## Results

 Scientific evidence indicates an increase in cumulative risk after 3–4 years of treatment. In such cases, control of the bacterial load should be prioritised and, as suggested by some authors, temporary discontinuation of the drug (drug holiday) should be considered. 

## Discussion

 This report presents the case of a 69-year-old female patient who had been treated with oral ibandronic acid for four years, and who was diabetic and on anticoagulant therapy. In line with scientific evidence, a safety protocol was established, comprising a multidisciplinary consultation followed by a ‘drug holiday’ (a four-month suspension) to optimise bone turnover prior to surgery. Systemic monitoring was carried out with antibiotic prophylaxis (aimed at preventing endocarditis due to her valvular disease and protecting the surgical site), as well as monitoring of the INR. The surgical phase consisted of multiple tooth extractions, pre-prosthetic mandibular surgery and Z-plasty of the upper labial frenulum. Success is attributed to an atraumatic technique and primary closure. 

## Conclusions

 Treatment with oral BF is not a contraindication; however, clinical success depends on individualised risk assessment, strict management of comorbidities and respect for bone biology.

# Abstract: 02 - SERUM VITAMIN D DEFICIENCY AND INSUFFICIENCY AND EARLY FAILURE IN DENTAL IMPLANTOLOGY

**Authors:** X. Hu Liu, S. Prin, M. Albánchez, M.J. Suárez García, J. Peláez Rico

**Affiliations:** *Máster Prótesis Bucofacial y Oclusión – Universidad Complutense de Madrid. Email: xinlliu22@gmail.com*

## Introduction

 Vitamin D deficiency may be a risk factor affecting the success of implant therapy due to its role in bone regeneration and remodeling during the osseointegration process. 

## Objectives

 to evaluate the current state of the literature regarding the relationship between low serum vitamin d levels and early failure of dental implants. 

## Material and Methods

 a literature search was conducted in the pubmed and scopus databases using the keywords “vitamin d deficiency,” “early implant failure,” and “osseointegration,” limiting the search results to the period between 2016 and 2026. Studies assessing serum vitamin d levels and reporting implant failures within the first months were included. systematic reviews, studies not reporting implant-related data, and animal studies were excluded.

## Results

 a total of 6 studies were included. half of them reported a higher failure rate in patients with low vitamin d levels, especially in cases of severe deficiency (<10 ng/ml). however, the other half did not find a statistically significant association. 

## Discussion

 the possible association in cases of severe deficiency could be explained by the role of vitamin d in regulating the inflammatory response and osseointegration. variability in the threshold defining vitamin d deficiency and in the definition of early failure makes comparisons difficult. 

## Conclusions

 at present, vitamin d deficiency cannot be considered a direct factor in early implant failure. more well-designed clinical studies with standardized criteria are required.

# Abstract: 03 - INTRAOPERATIVE DISPLACEMENT OF A DENTAL IMPLANT INTO THE MAXILLARY SINUS: NARRATIVE REVIEW AND PRESENTATION OF A CLINICAL CASE TREATED WITH A CALDWELL-LUC APPROACH

**Authors:** P. Maguregui Ortiz, A. García Marqués, P. Benito López, S. De Córdoba, S. De La Vega, I. Leco Berrocal

**Affiliations:** *Posgrado de Especialización en Cirugía Bucal e Implantología. Facultad de Odontología Universidad Complutense de Madrid. Email: pabmagur@ucm.es *

## Introduction and Objectives

 Displacement of dental implants into the maxillary sinus is an uncommon but potentially serious complication that may lead to sinus pathology. The aim of this study is to review the main causes, diagnostic approaches, and treatment options for this condition, as well as to present a clinical case. 

## Material and Methods

 A narrative review of the relevant scientific literature on implant displacement into the maxillary sinus was performed. We report the case of a 65-year-old patient with a history of maxillary sinus lift, in whom intraoperative displacement of a dental implant occurred during placement. Diagnosis was established by computed tomography, and surgical removal was carried out using a Caldwell-Luc approach. 

## Results and discussion

 The literature identifies residual bone deficiency, previous sinus lift procedures, and lack of primary stability as the main risk factors. The Caldwell-Luc approach allows direct visualization and controlled removal of the implant. In the present case, no signs of sinusitis were observed, and the postoperative course was uneventful, with no complications. 

## Conclusions

 Intraoperative displacement of dental implants into the maxillary sinus requires prompt management. The Caldwell-Luc approach remains a safe and effective option, particularly in patients with a history of sinus surgery. Proper preoperative planning and achieving adequate primary stability are essential to prevent this complication.

# Abstract: 04 - THE INFLUENCE OF OCCLUSAL SUPPORT ON COGNITIVE FUNCTION IN OLDER ADULTS: A NARRATIVE REVIEW OF THE LITERATURE

**Authors:** N. Pérez Rostoll, L. García Gonzalez, E. Bueno, J. Pelaez Rico, M.J. Suárez García

**Affiliations:** *Master’s Program in Oral and Maxillofacial Prosthetics and Occlusion, Complutense University of Madrid. Email: nataliarostoll99@gmail.com*

## Introduction

 Population aging has increased the prevalence of cognitive decline and dementia. Tooth loss and reduced occlusal support are common in older adults and may influence brain function through reduced masticatory stimulation, nutritional alterations, and systemic inflammation. 

## Objectives

 To critically evaluate the scientific evidence regarding the relationship between functional occlusal support and cognitive decline in adults over 65 years of age, as well as to analyze its clinical implications for geriatric prosthetic rehabilitation. 

## Material and Methods

 Systematic review following the PRISMA guidelines. A search was conducted in PubMed, Scopus, Web of Science, and the Cochrane Library. Studies in adults aged ≥65 years that assessed the number of teeth or occlusal support and cognitive function (MMSE, MoCA, or clinical diagnosis) were included. Six studies were selected: 2 longitudinal, 3 cross-sectional, and 1 meta-analysis. 

## Results

 A reduction in functional occlusal support was associated with an increased risk of cognitive decline. A lower number of teeth was linked to greater cognitive decline, whereas retaining ≥20 teeth or wearing functional dentures was associated with better cognitive outcomes. The meta-analysis showed an increased risk of cognitive decline (≈20%) and dementia (≈15%) in individuals with tooth loss. 

## Discussion

 The results show a consistent association, particularly in longitudinal studies. Possible mechanisms include decreased trigeminal stimulation, reduced cerebral perfusion, malnutrition, and systemic inflammation. However, Methodological Heterogeneity and observational design limit the establishment of causality. 

## Conclusions

 Tooth loss and reduced occlusal support are associated with cognitive decline. Their preservation and rehabilitation could contribute to healthy aging, although longitudinal studies and clinical trials are required to confirm this relationship.

# Abstract: 05 - EFFECTIVENESS OF PAPAIN IN THE DETECTION OF ACTIVE CARIES (BRIX 3000)

**Authors:** E. Báez Cruz, G. de Jaureguizar, N. Larrosa, V. Gil

**Affiliations:** *International University of Catalonia Email: bmd208579@uic.es*

## Introduction

 Contemporary dentistry is based on minimally invasive techniques aimed at presser-ving as much healthy dental tissue as possible. Brix 3000 is an enzymatic gel based on papain, de-signed to atraumatically remove carious tissue through chemical-mechanical caries removal, allo-wing for selective elimination of infected dentin. 

## Objectives

 To evaluate the efficacy of Brix 3000 in the chemical-mechanical removal of dentinal caries compared to conventional rotary Methods, and to assess its usefulness as a minimally invasive and conservative alternative that promotes preservation of affected tissue. 

## Material and Methods

 A literature review was conducted on the use of Brix 3000 in chemical-mechanical caries removal. The PubMed database was consulted, selec-ting clinical studies that analyzed efficacy in removing infected tissue, operative time, and pain perception. 

## Results

 Brix 3000 similar efficacy to rotary Methods in caries removal, with grea-ter preservation of healthy dentin and lower pain perception. However, it is associated with longer operative time, highlighting its role as a minimally invasive alternative in restorative dentistry. 

## Discussion

 The findings support the use of Brix 3000 within minimally invasive dentistry, emphasi-zing a more conservative approach and improved patient tolerance, especially in patients with spe-cial needs. 

## Conclusions

 Brix 3000 is an effective agent for chemical-mechanical caries removal, promoting selective elimination of infected dentin and preservation of affected dentin. It represents a conservative option within minimally invasive dentistry, particularly useful in special needs patients.

# Abstract: 06 - LICHEN PLANUS IN A PATIENT WITH GOOD’S SYNDROME: A CLINICAL CASE

**Authors:** A. Al-katan, D. Marín Fernández, D.P. Vallejos Rojas, P. Riutord Sbert, J. López López, B. González Navarro

**Affiliations:** *Faculty of Medicine and Health Sciences (Dentistry), University of Barcelona Email: ahmed.alkatan@yahoo.com*

## Introduction

 Lichen planus (LP) is a chronic inflammatory disease that can affect the skin, mucous membranes, nails, and hair. Good's syndrome is an acquired humoral immunodeficiency in adults associated with the presence of a thymoma. 

## Clinical Case

 A 64-year-old male patient with a history of thymoma, excised seven years before, was referred from the Catalan Health Institute for oral lesions consistent with oral lichen planus (OLP) bilaterally on both buccal mucosa and the left lateral border of the tongue. The patient reported burning, stinging, and generalized discomfort. A presumptive diagnosis of OLP was made, and a compounded 0.2% triamcinolone acetonide rinse was prescribed for two weeks. Recent blood tests were ordered, photographs of the oral and cutaneous lesions were taken, and a biopsy was planned. 

## Results and Discussion

 The biopsy showed (OLP). Many patients with Good's syndrome present with oral lichen planus, and some authors even consider it one of the clinical signs of the syndrome. However, there do not appear to be specific clinical forms of oral lichen planus associated with Good's syndrome. Treatment of the lichenoid disease in these patients is usually straightforward, and with treatment of the underlying problem, success rates of approximately 70.6% are typically achieved. Some authors recommend that in patients over 50 years of age with acute erosive OLP refractory to treatment, the presence of Good's syndrome should be investigated. 

## Conclusions

 The histopathological examination indicated that the lesions were compatible with oral lichenoid disease, requiring differential diagnosis from lupus, especially due to the discoid appearance of the lingual lesion.

# Abstract: 07 - IMPLANT REHABILITATION IN THE SEVERELY ATROPHIC MAXILLA: LATERAL SINUS LIFT VERSUS ZYGOMATIC IMPLANTS. A CASE REPORT

**Authors:** M. Arguimbau Coll, L. Maftei Rusu, L. Dumanova, D. Sánchez Cousiño, C. Arranz Obispo, J. López López

**Affiliations:** *Master’s Degree in Medicine, Oral Surgery and Implantology – University of Barcelona Email: m.arguimbaucoll@gmail.com*

## Introduction

 The rehabilitation of the severely atrophic upper jaw with vertical defects of less than 3 mm represents one of the main clinical challenges. Various techniques have been described for its treatment. In this regard, the following question arises: Which therapeutic option represents a greater benefit for treatment success and the patient: sinus lift with delayed implant placement or the use of zygomatic implants?. 

## Objectives

 To compare the therapeutic approaches described for the rehabilitation of the severely atrophic maxilla. 

## Case Description

 A 65-year-old female with a history of controlled type 2 diabetes and hiatal hernia. She presented with total edentulism for more than 15 years. The radiological study revealed severe posterior bone atrophy in the upper arch, with a residual height of 1-2 mm associated with significant sinus pneumatization. Different therapeutic options were compared, highlighting maxillary sinus lift and the use of zygomatic Implants. 

## Results

 An approach using bilateral sinus lifts was chosen. The technique was perfor-med via a lateral window, without intraoperative or postoperative complications. At 6 months, fa-vorable bone regeneration was achieved for implant placement. 

## Discussion

 Zygomatic implants allow for immediate loading and reduce the number of interventions, shortening treatment times; however, they are complex procedures, dependent on operator experience and associated with more major complications. Sinus lifting requires more time and surgical phases, but it favors better prosthetic planning and presents complications that are generally less severe and easier to resolve in the dental office. 

## Conclusions

 In severely atrophic maxillae, sinus lifting constitutes a valid and predictable therapeutic alternative, even in cases of minimal residual bone height. The choice of treatment must be based on the evaluation of patient-related factors and the clinician's experience.

# Abstract: 08 - IMPLANTOLOGY IN PATIENTS TREATED WITH DENOSUMAB: CASE REPORT AND CLINICAL CONSIDERATIONS

**Authors:** E. Carrasco Núñez, J. Parra Moreno, A. Estrugo Devesa, P. Egido Moreno, E. Jané Salas, J. López López

**Affiliations:** *Master’s Degree in Medicine, Oral Surgery and Implantology – University of Barcelona Email: Crakedwin@gmail.com*

## Introduction

 The use of monoclonal antibodies has emerged as a therapeutic strategy in patients with osteoporosis and a high risk of pathological fracture. However, their use has gained particular relevance in oral surgery due to their association with medication-related osteonecrosis of the jaws (MRONJ). Although the risk in osteoporotic patients is low (0.04–0.3%), it is slightly higher than that associated with bisphosphonates (0.01–0.1%). 

## Objectives

 To analyze the current evidence re-garding the management of patients undergoing denosumab therapy who require oral surgery, and to present a clinical case. 

## Case Description and Results

 An 82-year-old woman with a medical his-tory of Alzheimer’s disease, epilepsy, hypertension, glaucoma, hypercholesterolemia, chronic venous insufficiency, and osteoporosis—diagnosed in 2003 and initially treated with oral agents (2007–2013), followed by denosumab therapy since 2025—presented for placement of a single dental implant in position 24. Following medical consultation, it was decided to perform the pro-cedure one month prior to the next scheduled subcutaneous dose. The implant was placed using a conventional technique. Clinical and radiographic follow-up at 1, 2, 4, and 8 weeks demonstrated appropriate healing, with no signs of infection or complications. 

## Discussion

 Denosumab inhibits RANKL, thereby blocking osteoclastic activity and reducing bone remodeling. Unlike bisphospho-nates, it does not accumulate in bone, allowing for a reversible effect dependent on the dosing interval. Although no universal protocol exists, surgical intervention during the fifth month is consi-dered the safest timing, due to the progressive recovery of bone remodeling toward the end of the treatment cycle, which may favor healing and reduce complications. 

## Conclusions

 Dental implant placement in patients undergoing denosumab therapy can be safe if properly planned. Surgical timing, particularly during the final phase of the dosing interval—and individualized management are key factors in minimizing the risk of MRONJ.

# Abstract: 09 - One referral, two diagnoses: A case report

**Authors:** A. Pellicer Sánchez, L. Chen, A. Graterol, M. Apoita, S. Mishra, J. López

**Affiliations:** *Master's in Medicine, Surgery and Oral Implantology, University of Barcelona Email: anapellicer23@gmail.com*

## Introduction

 Accurate clinical assessment is essential in the diagnosis of complex oral Pathologies. Following an initial referral, a thorough anamnesis and complementary tests allowed us to simul-taneously identify primary Sjögren’s syndrome and oral lichen planus, highlighting the importance of a comprehensive patient-centered approach. 

## Case_Report

 We present the case of a 65-year-old female patient referred from primary care due to the presence of a bone spicule. Her relevant medical history included fibromyalgia, dyslipidemia, migraines, and anxiety-depressive disorders, along with chronic pharmacological treatment with potential xerostomic effects. Intraoral examination revealed multiple bilateral lesions on the buccal mucosa and retromolar area, with a whitish-reddish appearance, striated pattern, and associated symptoms, clinically compatible with oral lichen planus. Additional findings included desquamative gingivitis. Given the clinical con-text, a salivary function study was performed, demonstrating hyposalivation both at rest and under stimulation. Together with previous complementary tests (minor salivary gland biopsy and salivary gland scintigraphy), the ACR/EULAR 2016 classification criteria for primary Sjögren’s syndrome were fulfilled. To confirm the nature of the oral lesions, an incisional biopsy was performed, establishing the diagnosis of oral lichen planus. A multidisciplinary management approach was implemented, focused on symptom control, monitoring of potentially malignant lesions, and management of xerostomia. The patient was enrolled in a periodic follow-up program, emphasizing the importance of monitoring the progression of oral lichen planus. 

## Conclusions

 This case highlights the importance of a detailed anamnesis and a holistic evaluation of the patient, as two diagnoses—Sjögren’s syndrome and oral lichen planus—were established from a single referral.

# Abstract: 10 - ONE-PIECE IMPLANTS IN REDUCED SPACES: A MINIMALLY INVASIVE SOLUTION IN THE POSTERIOR MANDIBULAR SECTOR

**Authors:** M. Costa Tort, C. Polis Yanes, X. Xuan Qin, M. Allegue Allegue, B. González Navarro, J. López López

**Affiliations:** *Master’s Degree in Medicine, Surgery and Oral Implantology – Universitat de Barcelona – Faculty of Medicine and Health Sciences (Dentistry). Email: marccelta@gmail.com*

## Introduction

 rehabilitation of the posterior mandibular sector may be limited by reduced bone availability and limited mesiodistal space. in such cases, one-piece implants represent a therapeutic alternative that integrates the implant and abutment into a single structure, eliminating the microgap and simplifying treatment. their design allows use in narrow ridges and facilitates more conservative protocols, although precise planning is required due to the lack of prosthetic correction after pla-cement. 

## Objectives

 to describe the use of one-piece implants in the posterior mandible and to evaluate their feasibility in reduced spaces. Clinical case: A 65-year-old male patient attended our department for the rehabilitation of teeth 4.3 and 4.4 using microdent oneness® implants, along with immediate provisionalization, in a reduced mesiodistal space due to mesial migration of tooth 4.5. 

## Results and Discussion

 one-piece implants offer advantages such as elimination of the microgap and simplification of the surgical-prosthetic workflow. their indication is particularly useful in areas with space limitations or narrow ridges, potentially avoiding additional regenerative procedures. however, they require accurate three-dimensional planning and precise placement, as they do not allow for prosthetic corrections after insertion. evidence shows comparable outcomes to two-piece implants in well-selected cases. 

## Conclusions

 one-piece implants are an effective and predictable option in selected cases of the posterior mandible. they allow treatment simplification and adaptation to anatomical limitations, provided that proper planning and clinical execution are ensured.

# Abstract: 11 - INVESTIGATION OF A WHITE PLAQUE ON THE BUCCAL MUCOSA OF A PATIENT WITH A LONG-STANDING HISTORY OF VULVAR LICHEN SCLEROSUS: A CASE REPORT

**Authors:** D. Karunaikumar, V. Schiavo, S. Egido Moreno, B. González Navarro, E. Jané Salas, J. López López

**Affiliations:** *University of Barcelona. Master of Medicine, Surgery, and Oral Implantology Email: darian_k@hotmail.com*

## Introduction

 White plaques are frequent findings on the oral mucosa of geriatric patients, often poorly understood due to the wide range of causes, many of which are unknown. This is especially true in the case of oral lichen planus and lichenoid reactions. Their management is important due to their potential for malignant transformation. Treatment is complex, and improvement is often difficult to achieve, or the lesions tend to recur. Treatment depends on the diagnosis, but the similarities in the appearance of these lesions complicate matters. Furthermore, there is evidence of an association between oral and genital mucosal lesions. 

## Objectives

 We highlight the presentation of a white lesion on the oral mucosa found in a patient and its possible relationship to lichenoid disorders in other parts of the body. 

## Case description

 A 67-year-old patient attended the department with an asymptomatic whitish reticular lesion on the right buccal mucosa, of unknown origin, with a history of lichen sclerosus on the genital mucosa for 10 years. The preoperative analysis, histopathological study, and clinical follow-up were documented. 

## Results

 The biopsy confirmed the presence of inter-face stomatitis without dysplasia, consistent with a lichenoid reaction without criteria suggestive of malignancy. Follow-up of the lesion continues. 

## Discussion

 The treatment of lichenoid reactions requires continuous follow-up due to the possibility of malignant transformation over time, symptomatic management during periods of exacerbation, as well as the identification of causative factors to reduce or eliminate their presence, if possible. 

## Conclusions

 The findings support the relationship between oral and genital lichenoid disorders.

# Abstract: 12 - LESION OF THE FLOOR OF THE MOUTH: A CASE REPORT

**Authors:** D. Gil i Carandell, A. Borda Carrasco, B. Tejedor Coll, S. Egido Moreno, B. Gonzaìez Navarro, J. Lopez Lopez

**Affiliations:** *University of Barcelona. Master of Medicine, Surgery, and Oral Implantology Email: dolsgilc@gmail.com*

## Introduction

 Lesions of the floor of the mouth may arise from a variety of etiologies, including inflammatory, traumatic, and neoplastic processes. Accurate diagnosis requires clinical assessment and appropriate complementary investigations, considering the wide spectrum of pathologies that may occur in this anatomical region. 

## Case Report

 A 69-year-old woman presented with the acute onset of a painful lesion in the floor of the mouth, associated with xerostomia, after a recent dental prophylaxis with anesthesia. Clinical examination revealed a solitary, pedunculated nodular lesion, measuring 15 × 10 mm, located at the lingual frenulum. The lesion exhibited a reddish-white coloration, a smooth surface, and well-defined margins, with associated pain and a 4-day history. A differential diagnosis was established, including salivary gland pathology (sublingual sialadenitis), reactive lesions (pyogenic granuloma), subcutaneous emphysema, and neoplastic processes. Complementary investigations, including blood tests, intraoral radiographs, and cervical computed tomography, did not reveal any structural abnormalities or associated masses. Conservative management was initiated with antibiotic therapy (spiramycin and metronidazole), analgesia, and local measures. The clinical course was favorable, with a reduction of the lesion size and improvement in symptoms at follow-up. 

## Discussion

 The clinical presentation, with the recent history of local trauma, suggests a reactive inflammatory process. Sublingual sialadenitis typically presents as painful swelling associated with impaired salivary flow, whereas pyogenic granuloma represents a reactive vascular proliferation secondary to local irritation. Furthermore, differential diagnosis with conditions such as subcutaneous emphysema and neoplasms is essential. 

## Conclusions

 Acute lesions of the floor of the mouth require a systematic diagnostic approach to exclude neoplastic pathology. In cases of inflammatory or reactive origin, conservative treatment may be effective, leading to progressive clinical resolution. A meticulous follow-up is essential to confirm favorable outcomes and avoid misdiagnosis.

# Abstract: 13 - ORAL LEUKOPLAKIA WITHOUT DYSPLASIA WITH CLINICAL PROGRESSION: IMPORTANCE OF FOLLOW-UP AND SURGICAL MANAGEMENT

**Authors:** I. Jiménez, E. Jané Salas, C. Omaña Cepeda, V. Schiavo, B. González Navarro, J. López‑López

**Affiliations:** *University of Barcelona. Master of Medicine, Surgery, and Oral Implantology Email: Irina.jimenez.odo@gmail.com*

## Introduction

 Oral leukoplakia is a white lesion of the oral mucosa that cannot be clinically or histopathologically characterized as any other entity. It is the most common potentially malignant disorder of the oral cavity, with an estimated malignant transformation rate of approximately 9.8%. The main risk factors include tobacco use, alcohol consumption, and chronic trauma, with tobacco being the most significant. Lesions located on the lateral border of the tongue show a higher risk of malignant transformation, even in the absence of epithelial dysplasia. Clinical management remains challenging, particularly in cases where repeated biopsies fail to demonstrate dysplasia despite clinical progression. 

## Objectives

 To describe the clinical course and management of an oral leukoplakia, highlighting the importance of clinical surveillance and decision-making in the presence of lesion progression. 

## Case Report

 A 71-year-old female, smoking 20 cigarettes per day, presented with a white lesion on the right lateral border of the tongue. She was wearing a complete upper denture and a removable lower prosthesis with traumatic contact on teeth 4.7–4.8. The initial biopsy showed no evidence of dysplasia. Selective cusp adjustment was performed, and the lower prosthesis was removed for three months. The lesion persisted with clinical changes, and a second biopsy was also negative. After three months, the lesion developed a verrucous appearance, lea-ding to referral to maxillofacial surgery for complete excision. 

## Discussion

 This case highlights the discrepancy between clinical and histopathological findings in oral leukoplakia. The absence of dysplasia does not eliminate the risk of malignant transformation. Persistence after removal of local irritative factors suggests a multifactorial etiology, with tobacco playing a key role. Clinical progression should be considered a warning sign and must guide therapeutic decision-making, including surgical intervention. 

## Conclusions

 Oral leukoplakia requires close follow-up regardless of histopathological findings. Elimination of irritative factors is essential but not always sufficient. Clinical progression must be regarded as a red flag. In persistent lesions or those showing clinical evolution, surgical excision represents the most appropriate therapeutic approach.

# Abstract: 14 - COEXISTENCE OF ORAL LESIONS IN A SMOKING PATIENT: A CASE REPORT

**Authors:** C. Delgado Justo, A. Borda Carrasco, R. Prat Riera, J. Parra Morenos, B. González Navarro, J. López López

**Affiliations:** *University of Barcelona. Master of Medicine, Surgery, and Oral Implantology Email: claudiadelgado2000@hotmail.com*

## Introduction

 Tobacco use is one of the main risk factors in the development of oral lesions, which may present simultaneously with heterogeneous clinical features that complicate diagnosis. This situation requires clinicians to establish a broad differential diagnosis in order to rule out potentially malignant disorders. 

## Objectives

 The aim of this report is to describe the clinical and diagnostic approach to multiple oral lesions in a smoking patient. 

## Case Report

 We present the case of a 64-year-old male, a pipe smoker for 28 years, who attended the Dental Hospital of the University of Barcelona for implant evaluation. Intraoral examination revealed three lesions: a whitish plaque with multiple erythematous papules on the hard palate, clinically consistent with nicotinic stomatitis; a homogeneous leukoplakic plaque on the bilateral buccal mucosa; and a pedunculated nodular lesion on the left buccal mucosa suggestive of focal fibrous hyperplasia. Due to persistence of the palatal lesion after smoking cessation, an incisional biopsy was performed. Histopathological analysis revealed papillary hyperplasia and hyperkeratosis without atypia, confirming its benign nature. 

## Discussion and Conclusions

 Nicotinic stomatitis may mimic potentially malignant lesions; therefore, clinical follow-up and biopsy are essential to establish a definitive diagnosis and ensure appropriate management.

# Abstract: 15 - DIAGNOSIS OF A WHITE LESION ON THE LATERAL BORDER OF THE TONGUE: A CASE REPORT

**Authors:** A. Matinyan Hakobyan, J. Luengo Torrent, B. González Navarro, E. Jané Salas, S. Egido Moreno, J. López López

**Affiliations:** *Master’s Program in Oral Medicine, Surgery and Implantology. University of Barcelona Email: amatinma8@alumnes.ub.edu*

## Introduction

 Oral white lesions represent a diagnostic challenge due to their broad differential diagnosis, encompassing benign, reactive, and potentially malignant disorders. Early and accurate identification is essential for appropriate management and prognosis. 

## Objectives

 To describe the diagnostic approach to a white lesion of the tongue, highlighting key aspects of differential diagnosis and clinical criteria for suspicion. 

## Case Report

 A 62-year-old male patient presented with a whitish lesion on the right lateral border of the tongue. Clinical examination revealed a solitary plaque measuring approximately 12 × 9 mm, homogeneous, with a rough surface, well-defined margins, and non-scrapable. 

## Results

 The differential diagnosis included frictional hyperkeratosis and oral leukoplakia. The presence of a non-removable white lesion necessitates exclusion of traumatic causes. After identifying potential irritative factors, their elimination and subsequent reassessment are recommended. Persistence of the lesion after 2–4 weeks is suggestive of leukoplakia and requires histopathological confirmation by biopsy. Leukoplakia is defined as a white plaque that cannot be characterized as any other entity and carries a risk of malignant transformation, particularly in high-risk sites such as the lateral tongue. 

## Discussion

 The diagnosis of oral white lesions is primarily clinical and based on exclusion. Distinguishing between reactive lesions (frictional hyperkeratosis) and potentially malignant disorders (leukoplakia) relies on persistence, absence of an identifiable traumatic cause, and clinical characteristics. Biopsy remains the gold standard for definitive diagnosis. 

## Conclusions

 All non-removable oral white lesions require a systematic diagnostic approach and close follow-up. Persistence after elimination of local irritants should be considered an indication for biopsy to exclude leukoplakia and prevent malignant progression.

# Abstract: 16 - MAXILLARY VESTIBULOPLASTY WITH APICAL REPOSITIONING OF THE FLAP AND IMMEDIATE COMPLETE PROSTHESIS IN THE ATROPHIC EDENTULOUS MAXILLA: A CASE REPORT

**Authors:** M. Mesquida Alcover, L. Díaz Garrido, V. Schiavo Di Flaviano, M. Blázquez Hinarejos, B. Coll Tejedor, J. López López

**Affiliations:** *Master’s Degree in Oral Medicine, Surgery and Implantology, Faculty of Medicine and Health Sciences, School of Dentistry, University of Barcelona Email: mariamesquidaalcover@gmail.com*

## Introduction

 Prosthetic rehabilitation of the edentulous patient with an atrophic maxilla is complex when there is reduced vestibular depth, high muscle insertions and absence of functional keratinized tissue, which compromises prosthetic stability and retention. Vestibuloplasty represents a preprosthetic surgical alternative aimed at improving the anatomy of the recipient site and facilitating a more predictable conventional rehabilitation. 

## Objectives

 To describe the surgical-prosthetic approach in a completely edentulous patient with maxillary atrophy and severe vestibular deficiency, and to assess the clinical usefulness of vestibuloplasty. 

## Case description

 A 65-year-old female patient, with codeine intolerance, hypothyroidism, anxiety and arterial hypertension, under treatment with Eutirox, Lorazepam and Losartan, attended the Bellvitge University Dental Hospital for rehabilitation with complete bimaxillary prostheses. Clinical examination showed resorbed maxillae, shallow vestibules, high muscle and frenulum insertions, as well as absence of functional keratinized gingiva on the vestibular aspect. Maxillary vestibuloplasty with apical repositioning of the flap and release of muscle insertions was performed, followed by immediate placement of a maxillary complete denture acting as a conforming stent. 

## Discussion

: This technique increases vestibular depth through apical displacement of soft tissues and healing by secondary intention, without tissue grafting. It initially improves peripheral seal and prosthetic stability; however, it does not generate keratinized mucosa and presents a risk of relapse due to scar contraction and muscle reinsertion. 

## Conclusions

 Maxillary vestibuloplasty associated with immediate prosthesis may be an effective preprosthetic surgical option in selected cases of atrophic edentulous maxilla, improving the anatomical conditions for conventional rehabilitation.

# Abstract: 17 - FROM SEVERE ATTRITION AND OCCLUSAL COLLAPSE TO FUNCTIONAL SUCCESS: AN INTERDISCIPLINARY APPROACH TO FULL-ARCH REHABILITATION IN OLDER ADULTS: A CASE REPORT

**Authors:** N. El Ouaghmiri, J. Duque Ramírez, J.E. Aguilar Salgado, R. Ayuso Montero, M. Blázquez Hinarejos, J. López López

**Affiliations:** *Master’s Degree in Oral Medicine, Surgery and Implantology – University of Barcelona. Email: elouaghmirinissrine@gmail.com*

## Introduction

 Severe tooth wear of parafunctional origin, frequently associated with erosive tooth wear, may lead to occlusal collapse, loss of vertical dimension of occlusion (VDO), and functional impairment of the stomatognathic system. Bruxism has been identified as a risk factor for complications in implant-supported rehabilitations, with failure rates up to 2.2 times higher than in non-bruxist patients, highlighting the need for a carefully planned interdisciplinary approach. 

## Objectives

 To present a comprehensive clinical protocol for functional, aesthetic, and biological rehabilitation in a patient with severe tooth wear, loss of VDO, and posterior edentulism, based on current scientific evidence. 

## Clinical Case

A 60-year-old male patient with bruxism, presenting combined tooth wear (attrition and erosive wear) and a smoking habit, with a medical history of benign prostatic hyperplasia and low-grade tubular adenoma, attended for full-mouth rehabilitation. The treatment approach included: (1) functional analysis and digital 3D planning using CBCT and an articulator; (2) non-surgical periodontal therapy with management of furcation involvement; (3) occlusal reprogramming using provisional restorations; (4) prosthetically driven placement of posterior mandibular implants; and (5) tooth- and implant-supported rehabilitation with a mutually protected occlusal scheme. 

## Results

 The rehabilitation restored the VDO, re-established occlusal relationships, and improved masticatory efficiency and phonation. Periodontal stability and successful implant osseointegration were achieved. The provisional phase allowed validation of aesthetic and functional parameters, enhancing the predictability of the definitive outcome. 

## Discussion

 Preservation and treatment of the remaining dentition support maintenance of periodontal proprioception, physiological occlusal control, and gradual neuromuscular adaptation. This staged and reversible approach enhances biological preservation and reduces the risks associated with more aggressive treatment strategies. Recent evidence reports tooth survival rates of 82–100% at 5 years with conservative approaches, comparable to implant-supported rehabilitations. 

## Conclusions

 Rehabilitation of older adults with severe tooth wear and occlusal collapse requires a multidisciplinary approach integrating periodontal therapy and prosthetically driven implantology, representing a predictable and biologically sound strategy in gerodontology and advanced prosthodontics.

# Abstract: 18 - DESQUAMATIVE GINGIVITIS AS A MANIFESTATION OF PLASMA CELL GINGIVITIS: A CASE REPORT

**Authors:** E. Ortiz, M. Mesquida, V. Schiavo Di Flaviano, S. Egido Moreno, B. González Navarro, J. López López

**Affiliations:** *Master’s Degree in Oral Medicine, Surgery and Implantology. University of Barcelona Email: erikanathalyortizangueta@gmail.com*

## Introduction

 This report presents a patient with a blistering lesion affecting the oral mucosa, aiming to highlight the importance of differential diagnosis in cases of desquamative gingivitis. Plasma cell gingivitis is a rare, benign inflammatory condition often associated with hypersensitivity reactions. Clinically, it appears as erythematous and friable gingiva, which may mimic other disorders. Treatment typically involves topical corticosteroids and the removal of potential irritants. 

## Case presentation

 A 65-year-old woman attended the clinic reporting, “I’ve developed redness on my gums,” which she had first noticed about one month earlier. She reported no known allergies, was not taking any relevant medication, and denied any harmful habits. Her family history included colon cancer in her mother and breast cancer in her sister. Clinical examination revealed gingival erythema with multiple asymptomatic vesicles and a positive Nikolsky sign, with no evidence of lymphadenopathy. Blood tests showed no significant abnormalities. Histopathological analysis revealed mucosa lined by non-atypical squamous epithelium, with mild, nonspecific chronic inflammatory infiltrate and no signs of interface damage. Direct immunofluorescence was negative for IgA, IgM, and C3, allowing autoimmune blistering diseases to be ruled out. 

## Results and Discussion

 Desquamative gingivitis is a nonspecific clinical sign that may be associated with a range of conditions, most commonly autoimmune blistering diseases and inflammatory disorders. In this case, the initial clinical features suggested a possible blistering disease; however, histopathological findings and direct immunofluorescence ruled this out. The patient’s favorable response to topical treatment supported the diagnosis of nonspecific gingivitis, most likely plasma cell gingivitis, a benign condition diagnosed by exclusion. 

## Conclusions

 Careful evaluation of gingival lesions, supported by complementary tests such as biopsy and immunofluorescence, allows for an early and accurate diagnosis, facilitating timely and appropriate treatment.

# Abstract: 19 - PERIAPICAL SURGERY IN ELDERLY PATIENTS, AN UNDERUSED ALTERNATIVE TO IMPLANT REHABILITATION: A CASE REPORT

**Authors:** R. Prat Riera, P. Suarez Pérez, C. Polis Yanes, E. Jané Salas, B. González Navarro, J. López López

**Affiliations:** *Faculty of Dentistry, University of Barcelona, Bellvitge Campus. Email: remeiprat6@gmail.com*

## Introduction

 Periapical surgery is a last-resort option for tooth preservation after the failure of conventional endodontic treatment. In elderly patients, preserving the natural tooth becomes especially relevant compared to implant-based alternatives. Additionally, L-PRF has shown potential in bone regeneration and healing. 

## Objectives

 To evaluate periapical surgery as an alternative for tooth preservation in elderly patients and to assess the role of L-PRF as an adjunct in the treatment of extensive periapical lesions. 

## Case description

 We present the case of a 69-year-old patient with a periapical lesion in tooth 2.1, showing a cystic radiolucent lesion of approximately 10×10 mm involving both the buccal and palatal cortical plates. After failure of previous endodontic retreatment, periapical surgery was indicated. A mucoperiosteal flap was performed, followed by ostectomy, curettage, and enucleation of the lesion, apicoectomy at 3 mm from the apex, and retrograde filling with MTA. Subsequently, guided bone regeneration was carried out using a membrane and autologous L-PRF obtained by blood centrifugation. 

## Results

 Favorable postoperative course, without complications, with adequate soft tissue healing. Follow-up showed good clinical evolution and signs of progressive bone regeneration. 

## Discussion and Conclusions

 Periapical surgery allows tooth extraction to be avoided in selected cases, being especially relevant in elderly patients. Its combination with L-PRF may enhance healing and regeneration, representing an effective, biocompatible, and minimally invasive alternative to implant rehabilitation.

# Abstract: 20 - TONGUE LESION: DIAGNOSIS AND MANAGEMENT OF ORAL SQUAMOUS CELL CARCINOMA

**Authors:** S. Rivas Villalba, M. Blazquez, V. Schiavo Di Flaviano, S. Egido Moreno, E. Jané Salas, J. López López

**Affiliations:** *University of Barcelona. Master's Degree in Medicine, Surgery and Oral Implantology Email: susanabelenrv15@gmail.com*

## Introduction

 Potentially malignant oral lesions require early diagnosis to improve patient prognosis. Oral squamous cell carcinoma is the most common malignant neoplasm of the oral cavity, and its early detection is key to patient survival. 

## Objectives

 To describe the diagnostic, therapeutic and follow-up processes of a lingual lesion suspected of malignancy. 

## Case description

 A 70-year-old male patient, a smoker and regular alcohol consumer, presented with a painful lesion on the right ventral surface of the tongue that had been present for six weeks. Examination revealed a single, raised, reddish-white, ulcerated lesion with a broad base, firm consistency, and symptoms. A differential diagnosis was established, including both benign and malignant entities. An incisional biopsy was performed under local anesthesia, and histopathological analysis was conducted. 

## Results

 The histological study confirmed oral squamous cell carcinoma. The patient was referred for ultidisciplinary management. Staging and surgical planning were carried out using imaging techniques. A partial glossectomy and sentinel lymph node biopsy were performed. The patient presented with thrombocytopenia secondary to liver disease, requiring prior hematological management. The procedure was successful, and close follow-up was established. 

## Discussion

 Risk factors such as tobacco and alcohol use play a key role in the development of oral carcinoma. An Incisional biopsy is essential for definitive diagnosis. Sentinel lymph node biopsy is a useful tool in the early stages for assessing reginal spread. 

## Conclusions

 Early diagnosis and a multidisciplinary approach are essential for the management of oral squamous cell carcinoma. Accurate clinical identification of suspicious lesions enables early treatment and improves the patient prognosis

# Abstract: 21 - LEUKOPLAKIA ON THE LATERAL BORDER OF THE TONGUE: A CASE REPORT

**Authors:** L. Ruiz ópez, M. Mesquida Alcover, S. Egido Moreno, J. Parra, V. Schiavo Di Flaviano, J. López López

**Affiliations:** *Master's Program in Medicine, Surgery, and Oral Implantology, University of Barcelona Email: lruizlop11@gmail.com*

## Introduction

 In the differential diagnosis of white lesions of the oral mucosa, oral leukoplakia holds a predominant place, being the most common potentially malignant lesion of the oral cavity. 

## Case Description

 A 68-year-old female patient presents for mandibular rehabilitation using implants. The patient is a former smoker (10 cigarettes/day for 45 years) and has a history of hypothyroidism and depression, medicated with Eutirox® and Trepizol®. Clinically, a single white plaque is incidentally observed on the right lateral border and ventral surface of the tongue. The lesion has a homogeneous appearance with well-defined borders. It does not rub off upon scraping and is asymptomatic. Given the clinical suspicion of leukoplakia, an incisional biopsy is performed. The histopathological report confirmed the diagnosis of "homogeneous leukoplakia." Following the intervention, a post-operative check-up was conducted after 10 days, and a schedule of periodic reviews was established to monitor the stability of the lesion. 

## Discussion

 The differential diagnosis of white plaques is broad and complex. In this case, oral lichen planus was ruled out due to the absence of bilaterality and Wickham striae. Candidiasis was excluded as the lesion did not rub off upon scraping. 

## Conclusions

 Homogeneous leukoplakia is an entity that presents a lower risk of malignant transformation than non-homogeneous forms. Clinical management should focus on the elimination of risk factors such as tobacco and, in cases of low suspicion of malignancy like this one, on strict and prolonged clinical follow-up over time to early detect any changes in morphology or the appearance of atypia.

# Abstract: 22 - REVIEW OF THE MANAGEMENT OF THE OSTEOPOROTIC PATIENT: A CASE STUDY OF AN ELDERLY ADULT

**Authors:** E. Ibáñez Prieto, A. Flores, P. Ferrera Castellano, A. Bowen Varela, S. Bazal Bonelli, C.M. Madrigal Martínez Pereda

**Affiliations:** *Posgrado de especialización en Cirugía Bucal e Implantología UCM. Email: eibane02@ucm.es*

## Introduction

 Medication-related osteonecrosis of the jaw (MRONJ) is a rare complication associated with the treatment of osteoporosis. In patients receiving oral bisphosphonates (BF), the baseline risk is 0.1%, rising to 0.5% following surgical procedures, according to some authors. The presence of comorbidities such as diabetes mellitus exacerbates this risk by compromising microvascularisation and the immune response. 

## Objectives

 To update clinical guidelines for patients on long-term oral BF therapy and to evaluate the efficacy of preventive protocols in oral rehabilitation. 

## Material and Methods

 Protocols from leading bodies in Spain (SESPO, General Council of Dentists) were reviewed, as well as scientific literature from the last 10 years. Risk variables such as duration of exposure, drug potency and other systemic factors were analysed. 

## Results

 Scientific evidence indicates an increase in cumulative risk after 3–4 years of treatment. In such cases, control of the bacterial load should be prioritised and, as suggested by some authors, temporary discontinuation of the drug (drug holiday) should be considered. 

## Discussion

 This report presents the case of a 69-year-old female patient who had been treated with oral ibandronic acid for four years, and who was diabetic and on anticoagulant therapy. In line with scientific evidence, a safety protocol was established, comprising a multidisciplinary consultation followed by a ‘drug holiday’ (a four-month suspension) to optimise bone turnover prior to surgery. Systemic monitoring was carried out with antibiotic prophylaxis (aimed at preventing endocarditis due to her valvular disease and protecting the surgical site), as well as monitoring of the INR. The surgical phase consisted of multiple tooth extractions, pre-prosthetic mandibular surgery and Z-plasty of the upper labial frenulum. Success is attributed to an atraumatic technique and primary closure. 

## Conclusions

 Treatment with oral BF is not a contraindication; however, clinical success depends on individualised risk assessment, strict management of comorbidities and respect for bone biology.

# Abstract: 23 - ENDOCRINE DISRUPTORS IN DENTISTRY: PRESENCE, RELEASE, BIOLOGIC EFFECTS AND BIO BASED ALTERNATIVES IN DENTAL MATERIALS

**Authors:** A. García Sánchez, H. Benjilali, M. Mouhib, C. García López, C. Gutiérrez Bonet, J.A. Santos Marino

**Affiliations:** *Universidad de Salamanca y Universidad Complutense de Madrid Email: dentaladelagarcia17@gmail.com*

## Introduction

 Restorative dentists commonly use resin-based composites for their procedures. Nevertheless, there is an increasing concern about the possible release of endocrine activity particles, bisphenol A derivatives in particular (BPA). 

## Objectives

 Summerize the evidence available about the presence, release and biologic effects of this compounds in dental materials as well as identify the bio-based emerging alternatives. 

## Material and Methods

 An “scoping review” was done following the Joanna Briggs Institute and the PRISMA-ScR guide. The search was carried out in PubMed, Embase, Scopus and Web of Science with no time restriction. Experimental and clinical studies and reviews that evaluated the release of BPA and other estrogenic activity monomers were included, even their cytotoxicity and biocompatibility and the performance of other alternative biomonomers. 

## Results

 clinic evidence available shows the BPA exposure is generally low and temporary and the levels are mostly detected in salive and eventually urine. Vegetable sources derived biomonomers or biobased present a minor estrogenic activity, better biocompatibility and less potentially toxic monomers release, keeping similar or better mechanic properties than the traditional resins. 

## Discussion

 Though the dental material’s BPA exposure seems to be limited, there is still a knowledge gap between the standard Methods, the material composition transparency and the review of the clinical long-term behavior of the alternative biomonomers. 

## Conclusions

 Vegetable sources derived biomonomers or biobased seems to have less estrogenic activity, better biocompatibility and less potentially toxic monomers release, keeping similar or better mechanic properties than the traditional resins.

# Abstract: 24 - IMPACT OF POLYPHARMACY ON THE RISK OF ADVERSE EVENTS DURING DENTAL SEDATION IN OLDER ADULTS

**Authors:** J. Figueroa Romero, N. Larrosa, V. Gil, G. De Jaureguizar

**Affiliations:** *Universidad Internacional de Catalunya Email: jhosslinfigueroar@gmail.com*

## Introduction

 Population aging and the increasing prevalence of polypharmacy pose a significant challenge in dental practice, particularly in procedures that require sedation. Age-related pharmacokinetic and pharmacodynamic changes increase susceptibility to drug interactions and adverse events. However, the evidence integrating the impact of polypharmacy on the safety of dental sedation in medically compromised older adults is limited and dispersed. 

## Objectives

 To synthesize the scientific evidence on the relationship between polypharmacy and risk in dental sedation of medically compromised elderly adults. 

## Material and Methods

 Narrative review of the literature in biomedical databases, aimed at identifying drug interactions, physiological modifications associated with aging, and adverse events linked to sedation in geriatric dentistry. 

## Results

 Polypharmacy is associated with a significant increase in the risk of interactions between chronic drugs (especially cardiovascular and psychotropic) and sedative agents such as benzodiazepines and opioids. Age-related alterations, including decreased hepatic metabolism, renal clearance and increased permeability of the blood-brain barrier, promote drug accumulation and may cause adverse effects such as respiratory depression, hemodynamic instability and postoperative delirium. 

## Discussion

 The findings underscore the need for a thorough pharmacological evaluation prior to sedation, with an emphasis on identifying interactions, individualized dose adjustment and selection of the safest sedative agent based on the clinical profile of the patient.

# Abstract: 25 - ORAL MUCOSA AND SALIVA ALTERATIONS ASSOCIATED WITH VAPE USE

**Authors:** A. Alhek, B.F. Carmo Carvalho, L. Pedrosa Torres, B. Rivas Mundiña, C. Barrero López, A. Blanco Carrión

**Affiliations:** *University of Santiago de Compostela Email: alirakan1999@hotmail.com*

## Background

 Electronic nicotine delivery systems (ENDS), including e-cigarettes and vape devices, have become increasingly popular among adolescents and young adults. Despite their widespread use, the potential effects of vaping on oral tissues and salivary composition remain insufficiently understood. 

## Objectives

 To describe oral mucosal alterations and salivary changes associated with vape use through the clinical evaluation of a patient presenting with an oral lesion. 

## Material and Methods

 A 26-year-old male vape user presented with a rough white plaque located on the posterior region of the hard palate. The patient underwent a comprehensive clinical examination, sialometry, salivary pH evaluation, and excisional biopsy of the lesion. Saliva samples and vape liquid were analyzed using Fourier Transform Infrared Spectroscopy (FTIR) to investigate molecular composition and possible chemical alterations. 

## Results

 Histopathological analysis revealed hyperparakeratosis without epithelial dysplasia. Clinical examination also identified lesions compatible with nicotinic stomatitis. Salivary analysis showed normal flow rate and pH, although saliva viscosity was increased. FTIR spectroscopy detected the presence of compounds such as formaldehyde, ketones, and aromatic hydrocarbons in the vape liquid, substances classified as carcinogenic by the International Agency for Research on Cancer (IARC). Delayed healing was observed following biopsy, potentially associated with continued vape use and exposure to heat and flavoring compounds. 

## Conclusions

 Vape use may be associated with the development of hyperkeratotic oral lesions and alterations in salivary characteristics. Additionally, the presence of carcinogenic and cytotoxic compounds in vape liquids highlights potential risks to oral health. Further clinical studies are required to better understand the long-term impact of vaping on oral tissues.

# Abstract: 26 - WHEN AGING COMES EARLY: ORAL REHABILITATION IN WERNER SYNDROME AND ITS IMPLICATIONS FOR GERIATRIC DENTISTRY

**Authors:** C.A. Belo Faria, R. Stegun, N. Duarte, J. Duarte, I. Tómas, M. Gallottini

**Affiliations:** *Center for Special Care Dentistry (CAPE), School of Dentistry, University of São Paulo, Brazil. Email: carolbelo.cb@gmail.com*

## Introduction

 Werner syndrome is a rare autosomal recessive disorder characterized by premature aging and early onset of age-related comorbidities. Approximately 1,300 cases have been reported worldwide, with a higher prevalence in Japan. Its clinical overlap with geriatric conditions makes it a relevant model for understanding the challenges of dental management in medically complex patients. 

## Objectives

 To describe the dental management and oral rehabilitation of a patient with Werner syndrome, highlighting its implications for geriatric dentistry. Case Description: A 41-year-old female patient diagnosed with Werner syndrome, with a medical history of type 2 diabetes mellitus, osteoporosis, dyslipidemia, and renal impairment. Clinical findings included microstomia, xerostomia, tooth loss, and temporomandibular dysfunction, with severe impairment of masticatory function and esthetics. 

## Results

 Treatment required a multidisciplinary and individualized approach, including careful systemic evaluation, adaptation of clinical techniques due to limited mouth opening, and the use of thermoplastic prostheses to facilitate insertion and removal. Management of xerostomia and functional rehabilitation improved mastication, swallowing, and esthetics, contributing to the patient’s well-being. 

## Discussion

 This case demonstrates that Werner syndrome reproduces common features of geriatric patients, such as multimorbidity, polypharmacy, and functional limitations. The application of geriatric dentistry strategies was essential for safe and effective treatment. 

## Conclusions

 Werner syndrome anticipates challenges typically seen in geriatric dentistry, highlighting the importance of adapted strategies for patients with multimorbidity and functional limitations. Individualized oral rehabilitation was essential to improve masticatory function, swallowing, esthetics, and quality of life.

# Abstract: 27 - TELEDENTISTRY FOR AGEING POPULATIONS: A NARRATIVE REVIEW ON THE EMERGING ROLE OF ARTIFICIAL INTELLIGENCE AS AN INTELLIGENT INTERMEDIARY IN HOME-BASED ORAL CARE

**Authors:** I. Rodríguez, T. Martins, S.A. Mosaddad, J. Peláez, M.J. Suárez

**Affiliations:** *Master's degree in prosthetics at the Complutense University of Madrid Email: ikerrodr@ucm.es*

The rapid ageing of the global population is transforming oral healthcare delivery, with a growing proportion of older adults requiring care in home-based or dependent settings where access to conventional dental services remains limited. This narrative review aimed to evaluate the current applications and limitations of teledentistry in elderly populations and to explore the emerging role of artificial intelligence (AI) as a facilitator of remote oral healthcare. A comprehensive literature search was conducted across electronic databases, including PubMed, Scopus, and Web of Science, focusing on studies related to teledentistry, ageing populations, home-based oral care, and AI integration in healthcare. Relevant articles published in English were screened and selected based on their relevance to remote dental assessment, patient usability, and AI-assisted healthcare models. The reviewed evidence indicates that while teledentistry offers a promising approach to improving access to care, its effectiveness is currently limited by passive communication frameworks, inconsistent diagnostic reliability, and usability challenges among elderly patients. Importantly, findings suggest that the success of remote oral assessments is influenced more by the quality of data acquisition, patient guidance, and interaction dynamics than by the communication modality itself. Concurrently, AI has demonstrated expanding roles in geriatric healthcare as a supervisory, communicative, and decision-support tool; however, its application in dentistry remains predominantly diagnostic. The integration of AI as an intelligent intermediary within teledentistry frameworks may enhance communication, guide image acquisition, and support clinical triage, thereby improving the reliability and accessibility of home-based oral care. Despite existing challenges related to implementation, ethics, and digitalM literacy, AI-mediated teledentistry holds potential to shift oral healthcare delivery toward a more preventive, patient-centered model for ageing populations.

# Abstract: 28 - DO 3D-PRINTED AND MILLED COMPLETE DENTURES DIFFER IN MICROBIAL BEHAVIOR, BONDING PERFORMANCE, AND CLINICAL OUTCOMES? A SYSTEMATIC REVIEW AND META-ANALYSIS

**Authors:** A. Almojahed, S.A. Mosaddad

**Affiliations:** *Máster de Prótesis en la Universidad Complutense Madrid. Email: anas.almojahed@gmail.com*

## Objectives

 To comprehensively evaluate differences between additively manufactured (3D-printed) and subtractively manufactured (milled) complete denture bases across three key domains: microbial activity and adhesion, bonding performance, and clinical outcomes. 

## Material and Methods

 A systematic search of PubMed, Scopus, Web of Science, Embase, the Cochrane Library, and Google Scholar was conducted up to December 2024. Eligible studies included in vitro and clinical investigations directly comparing 3D-printed and milled complete denture bases. Outcomes of interest were microbial metabolic activity, microorganism adhesion, bond strength to denture teeth and reline materials, denture retention, and patient satisfaction. Study selection and data extraction were performed independently by two reviewers according to predefined criteria. Meta-analyses were conducted using random-effects models. Standardized mean differences (SMDs) were calculated for microbial, adhesion, reline bonding, retention, and satisfaction outcomes, while mean differences (MDs) were used for tooth-bond strength. Heterogeneity was assessed using Cochran’s Q, I², and τ² statistics. Publication bias and sensitivity analyses were performed where applicable. Risk of bias was assessed using QUIN for in vitro studies and RoB 2/ROBINS-I tools for clinical studies. 

## Results

 A total of 36 studies met the inclusion criteria. Microbial outcomes demonstrated significantly higher metabolic activity in 3D-printed denture bases compared to milled counterparts (SMD=1.285; 95% CI: 0.810–1.760; P<0.001), along with increased microbial adhesion (SMD=1.050; 95% CI: 0.124–1.976; P=0.026). Surface roughness was not identified as a significant predictor of microbial activity. Regarding bonding performance, milled denture bases exhibited significantly higher bond strength to denture teeth following sensitivity analysis (MD=−2.43 MPa; 95% CI: −3.90 to −0.96; P=0.001), and consistently superior bonding to reline materials (SMD=−2.62; 95% CI: −3.22 to −2.03; P=0.001). For clinical outcomes, no statistically significant differences were observed between manufacturing Methods in denture retention (SMD=0.165; 95% CI: −0.176 to 0.506; P=.343) or patient satisfaction (SMD=−0.595; 95% CI: −1.579 to 0.389; P=.236), although substantial heterogeneity was noted. 

## Conclusions

 Milled complete denture bases demonstrate more favorable laboratory performance, particularly in terms of microbial behavior and bonding reliability, compared to currently available 3D-printed materials. However, these differences do not appear to translate into significant clinical advantages in retention or patient satisfaction based on current evidence. Further high-quality clinical studies are required to determine the long-term clinical implications of these findings.

# Abstract: 29 - INTERDISCIPLINARY TREATMENT IN GERODONTOLOGY: A CLINICAL CASE REPORT

**Authors:** T. Martins, I. Rodríguez, F. Laciar, P. Angulo, J. Peláez, M.J. Suárez

**Affiliations:** *Postgraduate Specialization in Prosthodontics and Occlusion, Faculty of Dentistry, Complutense University of Madrid Email: tompires@ucm.es*

## Introduction

 An interdisciplinary approach is essential in gerodontology to rehabilitate patients with advanced periodontal disease and occlusal alterations, allowing for conservative and functional treatment options. 

## Objectives

 To describe the interdisciplinary management of a geriatric patient with advanced periodontitis, occlusal collapse, and functional and aesthetic impairment. 

## Case description

 A 65-year-old woman with type 2 diabetes presented seeking comprehensive oral rehabilitation. Clinical findings included missing teeth, hopeless prognosis of tooth 11, stage III grade B periodontitis, incisor proclination, deviation of the maxillary midline, severe recessions with generalized black triangles, posterior collapse, and anterior flaring with lack of overbite. Atypical swallowing with tongue interposition was diagnosed, and the patient was referred to speech therapy. Treatment included initial and non-surgical periodontal therapy (steps 1 and 2) and surgical therapy (step 3) in the first and third quadrants, extraction of tooth 11 with alveolar preservation, subsequent soft tissue augmentation and implant placement, removal of implant 37, and implant placement at site 35. Implants at 36, 46, and 47 were maintained. Orthodontic treatment involved intrusion and retrusion to restore occlusion. Prosthetic rehabilitation included provisional crowns for orthodontic anchorage, a provisional Maryland prosthesis, and definitive implant-supported zirconia restorations. 

## Results

 Improved occlusal stability, reduced tooth mobility, and correction of the midline were achieved. Atypical swallowing was significantly reduced. Aesthetic outcomes in periodontal tissues were limited due to reduced periodontium. No complications were observed during one-year follow-up. 

## Discussion

 and conclusions: Interdisciplinary treatment allows the management of complex geriatric cases through conservative approaches, achieving stable long-term outcomes.

# Abstract: 30 - RELATIONSHIP BETWEEN PROSTHETIC REHABILITATION AND NUTRITION AND HEALTH IN GERIATRIC PATIENTS

**Authors:** L. García González, N. Rostoll Pérez, J. Peláez Rico, M.J. Suárez García, E. Marquina Bueno

**Affiliations:** *Máster de Prótesis de la Universidad Complutense de Madrid. Email: luciagarciag22@gmail.com*

## Introduction

 The condition of a person’s dentition directly influences chewing function and nutrient intake, and is thus linked to nutritional status. The geriatric population suffers from tooth loss, making it necessary to use dental prostheses that do not provide the same masticatory efficiency as natural teeth. This limits food choices, leading to a preference for soft foods, fewer fruits, raw vegetables, or salads, and favoring diets high in fat and cholesterol and low in fiber, which impacts and compromises the patient’s quality of life. 

## Objectives

 To analyze the association between prosthetic rehabilitation and the nutritional habits and parameters of geriatric patients. 

## Material and Methods

 The scientific literature available in biomedical databases such as PubMed was analyzed, selecting articles that evaluated the relationship between dental and nutritional status in geriatric patients who retain their natural dentition and in those rehabilitated with various types of dental prostheses. 

## Results

 Prosthetic rehabilitation improves masticatory function and dietary diversity, with an increase in the intake of nutrients, proteins, carbohydrates, calories, and calcium. Anthropometric parameters such as body mass index showed heterogeneous results. The combination of prosthetic rehabilitation with dietary counseling demonstrates significant improvements in nutritional intake and health biomarkers. 

## Discussion

 Tooth loss is associated with reduced masticatory function and a less balanced diet, which can lead to potential nutritional deficiencies. Improving masticatory function through prosthetic rehabilitation positively contributes to better nutrition, although the impact depends on multiple factors. 

## Conclusions

 Prosthetic rehabilitation improves masticatory function and positively influences nutrition. However, the impact on nutritional status is variable. The combination of treatment and dietary counseling is more effective, and a multidisciplinary approach is essential for optimizing oral health and quality of life in geriatric patients.

# Abstract: 31 - COMPLICATIONS IN MAXILLARY SINUS ELEVATION: A LITERATURE REVIEW

**Authors:** P. Revuelta Cortés, A. Martínez Romero, A. García Marqués, P. Benito López, S. Saiz Carrasco, M. Hernández de Oliveira

**Affiliations:** *Postgraduate Program in Oral Surgery and Implantology, Complutense University of Madrid Email: pabrev01@ucm.es*

## Introduction

 In older adult patients, limited bone availability in the posterior maxilla is commonly encountered, making implant-prosthetic rehabilitation challenging. Several regenerative techniques have been developed to address this limitation, including maxillary sinus elevation. Despite its high success rates, complications may arise that can affect both the surgical procedure and the final treatment outcome. 

## Objectives

 The objectives of this review were: To analyze the prevalence of complications associated with maxillary sinus elevation; to identify risk factors related to these complications and to describe the management strategies used to resolve them. 

## Material and Methods

 A literature review was conducted based on recent articles addressing complications in sinus elevation procedures, including clinical studies and systematic reviews. 

## Results

 The most frequent intraoperative complication is Schneiderian membrane perforation, with a variable prevalence depending on the technique used. In minimally invasive approaches, rates are lower (1.9–3%), whereas significantly higher rates are reported with lateral window techniques. Among postoperative complications, maxillary sinusitis and infections are the most relevant, with reported prevalence ranging from 10% to 26%. Other complications described in the literature include peri-implant mucositis, epistaxis, vertigo, and graft loss.

## Discussion

 Complications are closely related to anatomical factors, surgical technique, and operator experience. Membrane perforation does not necessarily lead to treatment failure; however, it increases the risk of infection, compromises simultaneous implant placement, and may affect graft stability. The most common management strategy for membrane perforations is the placement of a collagen membrane to seal the defect. 

## Conclusions

 Maxillary sinus elevation is a safe and predictable procedure when proper planning based on radiological assessment is performed. Prevention, awareness of potential complications, and appropriate management are essential to maintain high implant success rates.

# Abstract: 32 - IMAGING DIAGNOSIS OF COMPLICATIONS ASSOCIATED WITH THIRD MOLAR EXTRACTION: A CASE REPORT

**Authors:** S. García Rodríguez, A. Bowen Varela, S. Dorsaf, P. Revuelta, I. Leco, C. Meniz

**Affiliations:** *Postgraduate Program in Oral Surgery and Implantology. Complutense University of Madrid. Email: soniagarcia9914@gmail.com*

## Introduction

 The extraction of impacted third molars is a common procedure in oral surgery, in which imaging diagnosis plays a key role in the prevention and detection of complications. Accidental displacement of third molars or root fragments into adjacent anatomical spaces, although infrequent (<1%), represents one of the most relevant complications. Proper radiological interpretation is essential to avoid diagnostic errors. 

## Objectives

 To evaluate, through a clinical case, the role of imaging diagnosis in the identification of a radiodense lesion in the mandibular angle. 

## Case Report

 A 63-year-old patient was referred due to transient discomfort, with a history of complicated extraction of tooth 4.8. Clinical examination revealed a hard, fixed, and well-defined lesion in the right mandibular angle. The initial radiological study suggested an osteochondroma as a presumptive diagnosis. Due to the poor image quality and atypical location, a second tomographic study was performed. Surgical removal via a cervical approach was planned, highlighting the correlation between clinical and radiological findings. 

## Results

 The tomographic study identified displaced root fragments of tooth 4.8 in the mandibular angle, ruling out the initial diagnosis and linking the finding to the previous surgical history. 

## Discussion

 The complication rate ranges from 2.6% to 30.9%, with displacement occurring most frequently into the submandibular space. This case highlights the importance of image quality, clinicoradiological correlation, and thorough anamnesis. 

## Conclusions

 Imaging diagnosis, together with clinical information, is essential for identifying complications and planning the appropriate surgical approach.

# Abstract: 33 - APPROACH USING A MODIFIED CALDWELL–LUC TECHNIQUE FOR THE RETRIEVAL OF A DENTAL ROOT FROM THE MAXILLARY SINUS: A CASE REPORT

**Authors:** M. Ouazzani Touhami, P. Ferrera Castellano, A. Bowen Varela, D. Sahli, L.M. Saez Alcaide, J. López Quiles

**Affiliations:** *Postgraduate Program in Oral Surgery and Implantology. Complutense University of Madrid. Email: m.ouazzani333@gmail.com*

## Introduction

 Root displacement into the maxillary sinus is a complication of extracting maxillary posterior teeth that may lead to sinusitis that is difficult to manage. In such cases, the modified Caldwell–Luc technique allows a direct and e active approach for removal of the foreign body. 

## Objectives

 To describe the surgical approach using the modified Caldwell–Luc technique for the retrieval of a root fragment accidentally displaced into the maxillary sinus. 

## Case Report

 A 60-year-old male presented with a root fragment in the maxillary sinus, localized by CBCT. Under local anesthesia, a Partsch incision (teeth 13–15) was performed, followed by elevation of a full-thickness flap. After creating an antrostomy in the anterior wall, the root fragment was removed, the inflamed sinus membrane was curetted, and primary closure of the tissues was achieved. Adjunctive medical therapy was prescribed to prevent postoperative infectious complications. 

## Results

 The extraction was successful, with a favorable postoperative course and moderate swelling that resolved within 7 days. At the 1-month follow-up, the patient showed no signs of oroantral communication or sinusitis, confirming complete tissue healing. 

## Discussion

 A precise surgical approach helps prevent chronic infectious complications. Despite the rise of endoscopic techniques, the modified Caldwell Luc approach remains e ective in oral surgery. Mucosal curettage, combined with a systemic pharmacological protocol, was key to restoring sinus function and preventing postoperative sinusitis. 

## Conclusions

 The modified Caldwell–Luc technique is a safe and predictable procedure for the retrieval of antral foreign bodies. Imaging-based diagnosis, careful soft tissue management, and appropriate pharmacological support are essential for therapeutic success and the absence of sequelae.

# Abstract: 34 - PRE-, PERI-, AND POSTOPERATIVE COMPLICATIONS IN DENTAL EXTRACCIONS IN OLDER ADULT PATIENTS

**Authors:** S. De Córdoba Fernández, A. González García, E. Ibáñez Prieto, M. Ouazzani Touhami, M. Sanmartí Oliver, J. Sanz Alonso

**Affiliations:** *Postgraduate Program in Oral Surgery and Implantology. Complutense University of Madrid. Email: serdecor@ucm.es*

## Introduction

 Tooth extraction is one of the most common procedures in dental practice. In older adult patients, its performance involves a higher risk due to the presence of comorbidities, drug interactions, and physiological changes associated with aging, which increase the likelihood of complications at different stages of the surgical procedure. 

## Objectives

 To analyze, through a literature review, the main complications associated with tooth extraction in older adults, classifying them into preoperative, intraoperative, and postoperative complications. 

## Material and Methods

 A literature review was conducted using scientific articles from databases such as PubMed, Scielo, and Elsevier. Relevant studies in Spanish and English related to complications of tooth extraction were selected, applying inclusion criteria based on older adult patients. 

## Results and Discussion

 A total of seventeen articles were reviewed. Preoperative complications are mainly associated with systemic diseases such as diabetes and hypertension, as well as the use of anticoagulants and antiplatelet drugs, which increase the risk of bleeding and require prior medical assessment. During the surgical procedure, complications such as bleeding, root fractures, nerve injuries, or damage to adjacent structures may occur. In the postoperative period, the most frequent complications are bleeding (up to 25%), alveolitis with an incidence between 3.7% and 5%, postoperative pain (up to 28%), infections, trismus, edema, and wound dehiscence, and in severe cases, hypovolemic shock or thromboembolic events. Additionally, in older adult patients, delayed healing is observed, as approximately 70% present systemic diseases, which increases susceptibility to complications. 

## Conclusions

 Older adult patients present a higher risk of complications, estimated to be up to 50% compared to younger patients. Adequate preoperative assessment, control of systemic and pharmacological factors, as well as careful surgical technique and proper postoperative follow-up, are essential to reduce associated morbidity and mortality.

# Abstract: 35 - CLINICAL COMPLICATIONS OF CAD/CAM-FABRICATED SUBPERIOSTEAL IMPLANTS IN ADULT PATIENTS IN ORAL REHABILITATION: A SYSTEMATIC REVIEW

**Authors:** A. González García, S. de Córdoba Fernández, A. Flores Fernández, P. Ferrera Castellano, C. Meniz García, C. Cobo Vázquez

**Affiliations:** *Postgraduate Program in Oral Surgery and Implantology. Complutense University of Madrid. Email: anagon44@ucm.es*

## Introduction

 Subperiosteal implants represent a therapeutic alternative in the oral rehabilitation of patients with severe bone atrophy, where conventional implants are not feasible. The incorporation of CAD/CAM technologies has enabled the design of customized structures that precisely adapt to the bone, improving biomechanical performance and reducing surgical time. However, it is necessary to evaluate the clinical complications associated with these implants. 

## Objectives

 The aim of this study is to systematically evaluate the available scientific evidence on the clinical complications of CAD/CAM-fabricated subperiosteal implants, as well as to analyze their impact on design, fit, performance, and the incidence of complications in oral rehabilitation. 

## Material and Methods

 A systematic review of the literature from the last five years was conducted, including studies in Spanish and English (clinical trials, systematic reviews, and case reports). The search included terms related to subperiosteal implants, oral surgery, alveolar bone loss, and complications. From an initial total of 43 articles, 8 were selected for final analysis. 

## Results and Discussion

 CAD/CAM subperiosteal implants show a high functional survival rate (95–98% at 12- 24 months). However, frequent complications were identified, especially in soft tissues, such as framework exposure (20–30%), dehiscence (5–10%), and localized infections. These complications are associated with factors such as inadequate design, thin gingival phenotype, flap tension, or poor oral hygiene. Additionally, mechanical problems such as poor fit, micromovements, or prosthetic fractures (4–6%) may compromise treatment success. Precision in fit and customized design are key to minimizing risks. 

## Conclusions

 CAD/CAM-fabricated subperiosteal implants constitute an effective option in cases of severe atrophy, with high success rates. However, clinical complications remain relevant and largely depend on implant design, surgical technique, and patient maintenance. A multidisciplinary approach and proper planning are essential to optimize clinical outcomes.

# Abstract: 36 - DENTAL IMPLANTS IN PATIENTS AGED ≥75 YEARS: SURVIVAL, COMPLICATIONS, AND GERIATRIC VARIABLES; A SYSTEMATIC REVIEW OF THE LAST 10 YEARS

**Authors:** P. Benito López, M. Ouzzani Touhami, S. García Rodriguez, J. Jimenez, A.E. Cáceres Madroño, L. Hernando Calzado

**Affiliations:** *Postgraduate Program in Oral Surgery and Implantology. Complutense University of Madrid. Email: pbenit03@ucm.es*

## Introduction

 Population aging has increased the demand for dental implant therapy in older adults. However, evidence in patients aged ≥75 years must be interpreted considering frailty, comorbidities, polypharmacy, and self-care capacity. 

## Material and Methods

 A systematic review was conducted following PRISMA 2020 guidelines to identify studies published between 2016 and 2026 in PubMed/MEDLINE, Web of Science, and the Cochrane Library. The PICO framework included patients ≥75 years (P) undergoing implant rehabilitation (I), compared with younger adults (C), to assess survival, success, and complications (O). Study selection was based on title/abstract and full-text screening. Data extraction was standardized, and risk of bias was assessed using RoB 2 and the Newcastle–Ottawa Scale. 

## Results

 Twenty studies met the inclusion criteria. Evidence indicates high implant survival in older adults, suggesting that chronological age alone is not a contraindication. In patients ≥75 years, medium-term survival rates (1–5 years) ranged from 87.3% to 98%. Poor oral hygiene, limited follow-up, and certain comorbidities were associated with increased risk of peri-implant diseases and early implant loss. Immediate loading protocols (All-on-4/6) showed comparable survival to conventional loading, with potential differences in prosthetic complications. In atrophic jaws, short implants emerged as a less invasive alternative with reduced morbidity and similar medium-term outcomes. Economic evidence remains scarce. 

## Conclusions

 Implant therapy in patients ≥75 years is predictable. Clinical decisions should prioritize biological and functional status over age alone. Further research incorporating frailty metrics and cost analyses is warranted.

# Abstract: 37 - GUIDED IMPLANT SURGERY IN AN ELDERLY PATIENT: A CASE REPORT AND NARRATIVE REVIEW

**Authors:** P. Ferrera Castellano, K. Ivaylova Serkedzhieva, A. González García, A. Bauer González, G. Ochoa López

**Affiliations:** *Postgraduate Specialization in Oral Surgery and Implantology from the Complutense University of Madrid. Email: pferrera@ucm.es*

## Introduction

 Population ageing poses new challenges in surgical planning, particularly in implant dentistry, where precision and complication reduction are essential. Guided surgery has emerged as a tool that may improve safety, especially in elderly patients. 

## Objectives

 To describe the applicability of guided implant surgery in an elderly patient and to analyse, through a narrative review, the potential benefits of these techniques and their relevance in the geriatric population. 

## Material and Methods

 A clinical case of guided implant surgery in an elderly patient is presented, along with a narrative review of recent literature on guided surgery and its possible application in older adults. Variables such as surgical accuracy, operative time, complications and postoperative recovery are evaluated. 

## Results

 Evidence shows that guided surgery allows for more precise planning and controlled execution, reducing complications in the general population. These benefits may be particularly relevant in elderly patients due to the frequent presence of comorbidities and reduced recovery capacity. 

## Discussion

 Although specific evidence in the geriatric population is limited, the advantages of guided surgery suggest potential benefits in this group. However, limitations such as cost, need for specific training and variability in outcomes must be considered, and indications should be individualised. 

## Conclusions

 Guided surgery is a promising tool in implant dentistry for elderly patients. Further studies focused on the geriatric population are needed to better define its indications and benefits.

# Abstract: 38 - LESS IS MORE: SHORT IMPLANTS AS AN ALTERNATIVE TO REGENERATION IN THE POSTERIOR REGION

**Authors:** M. Andrino, J. Tatari, M. Estefan, P. Diaz, J. Peláez, M. Suarez

**Affiliations:** *Faculty of Dentistry, Master’s Program in Maxillofacial Prosthetics and Occlusion, Complutense University of Madrid. Email: marandri@ucm.es*

## Introduction

 Rehabilitation of the posterior region with limited bone height remains a challenge in implantology. the use of short (< 8.5 mm) and extra-short (< 6.5 mm) implants has emerged as a less invasive alternative for prosthetic rehabilitation of edentulous areas where bone regeneration is not an option. 

## Objectives

 to describe the rehabilitation of the posterior mandibular region using short and extra-short implants as an alternative to bone augmentation procedures. 

## Material and Methods

 a 70-year-old female patient attended the master’s program in maxillofacial prosthetics and occlusion, referred from the implantology program, for rehabilitation of the left posterior region where pre-existing short and extra-short implants (34, 36, and 37) were present. this treatment had previously been carried out due to a history of vertical bone regeneration and connective tissue grafting with therapeutic failure. therefore, prosthetic treatment was performed. implant positions were captured using an intraoral scanner (3shape) to request a rigid impression splint (ris) from the laboratory and obtain a high-precision impression with passive fit, there by avoiding possible distortions, given the patient’s lack of keratinized gingiva. Subsequently, passivity and metal framework try-ins were performed. Once a proper fit was confirmed, porcelain application was requested, concluding with the final placement of the prosthesis. 

## Results

 the rehabilitation restored the patient’s masticatory function and aesthetics, with proper prosthetic adaptation. 

## Discussion

 currently, the use of short and extra-short implants in the rehabilitation of edentulous areas with significant vertical bone loss is becoming an increasingly viable therapeutic option. recent studies show favorable outcomes, with less bone loss and good prosthetic performance, while avoiding more invasive surgical procedures. however, medium- and long-term evidence is still limited. 

## Conclusions

 The use and rehabilitation of short and extra-short implants constitute an effective and less invasive alternative to bone regeneration techniques, offering predictable functional and aesthetic outcomes.

# Abstract: 39 - DENTAL IMPLANTS IN PATIENTS AT RISK OF OSTEONECROSIS

**Authors:** J. Jimenez Aracil, D. Sahli, P. Revuelta Cortes, P. Maguregui Ortiz, J. Cortes, R. Martin

**Affiliations:** *Faculty of Dentistry, Complutense University of Madrid. Email: jorgegji@ucm.es*

## Background

 Antiresorptive drugs impair bone remodeling and may compromise the healing of dental implants, increasing the risk of medication-related osteonecrosis of the jaw (MRONJ). 

## Objectives

 To evaluate the incidence of osteonecrosis in patients receiving dental implants while undergoing antiresorptive therapy and to analyze available therapeutic approaches according to disease severity. 

## Material and Methods

 A literature review was conducted using the PubMed database with the keywords “osteonecrosis of the jaw,” “dental implant,” and “management.” Inclusion criteria comprised original studies published within the last ten years and available in open access. 

## Results

 Nine studies met the inclusion criteria. Overall, a high success rate of dental implant placement was reported in patients receiving antiresorptive therapy. However, complications such as delayed wound healing and impaired osseointegration were observed. These findings emphasize the need for comprehensive patient assessment and meticulous treatment planning. Despite these risks, implant-supported oral rehabilitation provides significant functional and psychosocial benefits. 

## Discussion

 Clinical management is primarily guided by the recommendations of the American Association of Oral and Maxillofacial Surgeons. Early-stage disease (stages I–II) is generally managed conservatively, whereas advanced cases (stage III) require surgical intervention, including debridement and resection of necrotic bone. Adjunctive therapies, such as platelet concentrates and laser therapy, have been proposed; however, their efficacy remains inconclusive. 

## Conclusions

 Dental implant rehabilitation in patients receiving antiresorptive therapy is a feasible treatment option when careful patient selection and interdisciplinary management are implemented. Treatment of associated complications should be tailored to disease severity and individual patient characteristics.

# Abstract: 40 - IMMEDIATE IMPLANTS AND INMEDIATE LOADING IN GERIATRIC PATIENTS: A LITERATURE REVIEW

**Authors:** A. Flores Fernández, E. Ibáñez Prieto, M. Ouazzani Touhami, S. García Rodriguez, L. Hernando Calzado, A. Bauer González

**Affiliations:** *Postgraduate Program in Oral Surgery and Implantology. Complutense University of Madrid. Email: aff.adrian2002@gmail.com*

## Introduction

 Population aging has increased the demand for implant therapy in geriatric patients. Traditionally, advanced age was considered a limiting factor; however, current evidence suggests that implant therapy can be predictable even in older patients. 

## Material and Methods

 A narrative literature review was conducted in PubMed, including prospective and retrospective clinical studies as well as systematic reviews evaluating immediate implants or immediate loading in geriatric patients. Variables such as implant survival, marginal bone loss, biological and prosthetic complications, and associated systemic factors were considered. Studies involving non-geriatric populations or lacking clinical follow-up were excluded. 

## Results

 The analyzed studies show high success rates for immediate implant and immediate loading protocols in geriatric patients. A clinical study with immediate fixed rehabilitations in geriatric patients reported an implant survival rate of 98.5%. Studies using guided surgery and immediate loading in older patients showed success rates close to 100%, with marginal bone loss around 1.37 mm. Systematic reviews indicate that implant survival in geriatric patients exceeds 96% at five years, with no clinically relevant differences compared to younger patients. 

## Discussion

 Chronological age does not constitute a contraindication for immediate implant placement. The favorable outcomes observed may be attributed to multiple factors, such as improvements in implant design and advances in surgical techniques. However, geriatric patients present specific characteristics such as multimorbidity, polypharmacy, and reduced oral hygiene capacity, which may increase the risk of peri-implant biological complications. Patient selection, primary stability, and maintenance are critical factors for treatment success. 

## Conclusions

 Immediate implants and immediate loading represent a predictable therapeutic alternative in selected geriatric patients. Individualized treatment planning, adequate primary stability, and maintenance protocols are key to achieving successful outcomes.

# Abstract: 41 - CHATGPT IN ORAL PATHOLOGY

**Authors:** M. Suárez Ajuria, A. Suárez, Y. Freire, V. Díaz-Flores García, J. Algar, C. Martín Carreras-Presas

**Affiliations:** *Universidad Europea de Madrid Email: mariasuaj@gmail.com*

## Introduction

 The growing academic interest within the biomedical sciences regarding the diagnostic capabilities of multimodal language models, such as ChatGPT-4o, is clear. However, their ability to interpret oral clinical images remains insufficiently explored. This exploratory pilot study aimed to provide preliminary observations about the diagnostic validity of ChatGPT-4o in identifying oral squamous cell carcinoma (OSCC), oral leukoplakia (OL), and oral lichen planus (OLP) using only clinical photographs, without the inclusion of additional clinical data. 

## Material and Methods

 Two general dentists selected 23 images of oral lesions suspected to be OSCC, OL, or OLP. ChatGPT-4o was asked to provide a probable diagnosis for each image on 30 occasions, generating a total of 690 responses. The responses were then evaluated against the reference diagnosis set up by an expert to calculate sensitivity, specificity, predictive values, and the area under the ROC curve. 

## Results

 ChatGPT-4o demonstrated high specificity across the three conditions (97.1% for OSCC, 100% for OL, and 96.1% for OLP), correctly classifying 90% of OSCC cases (AUC = 0.81). However, this overall accuracy was largely driven by correct negative classifications, while the clinically relevant sensitivity for OSCC was only 65%. In spite of that, sensitivity was highly variable: 60% for OL and just 25% for OLP, which limits its usefulness in a clinical setting for ruling out these conditions. 

## Conclusions

 ChatGPT-4o demonstrates potential as a complementary tool for the screening of OSCC in clinical oral images. Nevertheless, the pilot nature of this study and the reduced sample size highlight that larger, adequately powered studies, are needed to obtain robust and generalizable results.

# Abstract: 42 - METABOLOMICS IN THE ASSESSMENT OF ROOT CARIES RISK

**Authors:** J.C. Ortola Siscar, C. Ortola Piera, T. Almerich Torres, O.A. Musalem Dominguez, J.M. Almerich Silla

**Affiliations:** *Departamento de Estomatología. Facultad de Medicina y Odontología de la Universidat de València. Email: c.ortolasiscar@gmail.com*

## Introduction

 Root caries affects 31.6% of the Spanish population between 65 and 74 years of age. Clinical examination allows us to identify established lesions, so we believe that improving early diagnosis is necessary. This can be achieved by using known risk factors (diet, oral hygiene, medications that affect salivary secretion) and others that may become available with the development of omics techniques. Metabolomics is the branch of omics that studies metabolites (amino acids, lipids, and carbohydrates) produced by bacterial cellular processes or by the interaction between the pathogen and its host. 

## Objectives

 To determine the capacity of a predictive model for caries based on the metabolomic profile of saliva and biofilm in elderly patients, looking for metabolomic differences in bacterial plaque and saliva in patients over 60 and under 80 years of age, with and without caries. 

## Material and Methods

 This is an observational, cross-sectional, and analytical case-control study. As this is an exploratory study, given the lack of previous research, 100 patients will be selected for each study group. A medical history will be taken, including a record of medications taken by the patients. A clinical examination will be performed following the ICDAS-II and CPITN criteria, and saliva and biofilm samples will be collected after obtaining informed consent. An independent samples t-test will be used to compare the mean levels of different metabolites in the two patient groups (cases and controls). 

## Conclusions

 We propose an exploratory study protocol to determine the possible existence of metabolomic profiles that may be associated with the presence of root caries in older adult patients.

# Abstract: 43 - ASSOCIATION BETWEEN PERIODONTAL DISEASE AND ALZHEIMER’S DISEASE: CURRENT EVIDENCE AND INVOLVED MECHANISMS

**Authors:** M. Rodríguez Sanz, L. Álvarez Barrañeda, C.M. Buelga González, C. Jacobo Orea, M.A. Alobera Gracia, M. del Canto Pingarrón

**Affiliations:** *Máster de Cirugía, Implantología y Periodoncia de León. Email: martadentista@hotmail.com*

Periodontal disease (PD) and Alzheimer’s disease (AD) are two highly prevalent conditions that share several risk factor and common pathophysiological mechanisms, including chronic inflammation. In recent years, several studies have suggested a possible relationship between these two diseases, particularly through systemic inflammatory processes and the action of periodontal pathogens. The aim of this study was to analyze the available scientific evidence regarding the potential association between periodontal disease and Alzheimer’s disease, as well as the biological mechanisms involved and the role of periodontal bacteria in this process. A literature review was conducted using databases such as PubMed, Cochrane, and Google Scholar. Systematic reviews and meta-analyses published between 2021 and 2026 in English that examined the relationship between both conditions were included. After applying the inclusion and exclusion criteria and performing a critical appraisal of the articles, a total of 15 studies were selected for analysis. The results show that most studies report a positive association between PD and the development or progression of AD. The most frequently described mechanisms include chronic systemic inflammation, the release of bacterial lipopolysaccharides, and the possible translocation of periodontal pathogens or their products to the central nervous system, promoting Neuroinflammation. Among the most frequently implicated bacteria are Porphyromonas gingivalis, Treponema denticola, and Fusobacterium nucleatum, with P. gingivalis being the pathogen with the strongest evidence regarding its association with AD. In conclusion, current evidence suggests a possible association between PD and AD, mainly mediated by inflammatory and microbiological mechanisms. However, the Methodsological heterogeneity of the studies and the presence of confounding factors limit the causal interpretation of this relationship. Therefore, further standardized research is required to confirm these findings.

# Abstract: 44 - PERIODONTAL REGENERATION IN INTRABONY DEFECTS: TECHNIQUES WITH THE STRONGEST SCIENTIFIC EVIDENCE

**Authors:** N. Dorronsoro Erauntzetamurgil, O. Dorronsoro Eizmendi, D. Urreta Cardador, M. del Canto Díaz, M. del Canto Pingarrón, M.A. Alobera Gracia

**Affiliations:** *Master’s Degree in Oral Surgery, Implantology and Periodontology. León. Email: neredorron1@gmail.com*

## Introduction

 periodontal regeneration in intrabony defects represents one of the main therapeutic challenges in periodontology. over the last decades, multiple biomaterials and surgical approaches have been developed, making it necessary to identify those with the strongest scientific support. 

## Objectives

 to analyze the recent scientific evidence on regenerative techniques used in intrabony defects and to determine which ones present the strongest Methodological support and the most consistent clinical outcomes. 

## Material and Methods

 a structured search was conducted in pubmed (2020–2025) using terms related to “periodontal regeneration”, “intrabony defects”, “systematic review”, and “randomized controlled trial”. high-level Methodological studies in humans (systematic reviews, network meta-analyses, and randomized controlled clinical trials) were included. after applying the inclusion and exclusion criteria, 15 studies were selected for qualitative analysis. 

## Results

 systematic reviews and network meta-analyses show that enamel matrix derivatives (emd) and certain recombinant growth factors, such as rhfgf-2, provide significant improvements in clinical attachment level gain (cal) and probing depth reduction (pd). combinations of biomaterials with bone grafts also show favorable outcomes. Autologous platelet concentrates demonstrate additional clinical benefits, although with greater heterogeneity. Minimally invasive surgical techniques are associated with stable clinical outcomes and adequate tissue preservation in the medium and long term. 

## Discussion

 current evidence suggests that there is no universally superior technique; rather, predictability depends on correct clinical indication and the surgical approach used. The biomaterials with the strongest scientific support are emd and certain recombinant growth factors, whereas platelet concentrates show promising but variable results. minimally invasive surgery appears to enhance the stability of regenerative outcomes. 

## Conclusions

 periodontal regeneration in intrabony defects is supported by solid scientific evidence, especially for emd and recombinant growth factors. individualized selection of the biomaterial and the surgical technique is essential to optimize clinical outcomes.

# Abstract: 45 - TOOTH EXTRACTION PROTOCOL IN PATIENTS TREATED WITH BONE ANTIRRESORPTIVE AGENTS. A CASE REPORT

**Authors:** A. Somodevilla Mangas

**Affiliations:** *Primary Care Dentist at the Torrox Health Center. Email: javiera.espinoza@utalca.cl*

## Introduction

 Currently, it is common to encounter patients in our dental clinics who are medicated with bone antiresorptive agents for bone metabolism problems. When the risk of bone fractures is high, the tendency is to prescribe bone antiresorptive agents parenterally, including prolia 60 mg. this medication provides proven benefits in bone density and fracture reduction, but it also has side effects that must be understood and avoided whenever possible. Among these side effects is osteonecrosis of the jaw. 

## Objectives

 To update, through a clinical case, the protocol for dental extractions in patients taking bone-resorptive medications. 

## Case Report

 An 85-year-old woman. no known allergies or history of substance abuse. she is on multiple medications for systemic diseases: hypertension, renal insufficiency, osteoporosis, multiple arthropathies, dyslipidemia, gout, peripheral artery disease, and anxiety. She has a history of femur fracture surgery. She presents with impaired physical mobility, a risk of falls, and difficulty with self-care. She comes to the dental office reporting instability of lower denture and difficulty chewing. 

## Results

 Antibiotic prophylaxis with amoxicillin 500 mg every 8 hours for 7-10 days, chlorhexidine 0.2% rinse every 8 hours for 7-10 days, no drug holiday, extraction of the remaining root of tooth 3.7 under written informed consent, atraumatically, without bone regularization and closure by secondary intention. subsequent healing monitoring. Mini ball implant after 40 days.

# Abstract: 46 - EFFICACY OF LOW-POWER LASER IN THE TREATMENT OF RECURRENT COLD SORES

**Authors:** I. Craveiro Lima, N. Eljaky, V. Sanmartín Barragáns, A. Blanco Carrión, P. Gándara Vila, I. Prado Pena

**Affiliations:** *University of Santiago de Compostela Email: craveirolima@gmail.com*

## Introduction

 Recurrent herpes labialis (RHL), caused by the herpes simplex virus type 1 (HSV-1), is a highly prevalent condition characterized by repeated episodes of painful lesions in the perioral region. Conventional treatments, such as acyclovir, present imitations in terms of clinical efficacy, patient adherence, and recurrence, which has driven the search for alternative therapies such as low-level laser therapy or photobiomodulation (PBMT). 

## Objectives

 To analyze the available scientific evidence on the effectiveness of low-level laser therapy in reducing pain, promoting healing, shortening recovery time, and decreasing recurrence in patients with recurrent herpes labialis. 

## Material and Methods

 A scoping review was conducted following the PRISMA 2020 guidelines. The search was performed in PubMed and Scopus (February 2026), including studies in English and Spanish that evaluated the use of low-level laser therapy in the treatment of RHL. Clinical trials, comparative studies, and experimental studies reporting relevant clinical outcomes were analyzed. 

## Results

 Nine studies with heterogeneous designs were included. Most clinical studies showed a significant reduction in pain and a decrease in recovery time in the laser-treated groups, in some cases superior to treatment with acyclovir. Additionally, several studies reported accelerated healing and improved lesion progression. Regarding recurrence, although the number of studies is limited, a trend toward a reduction in outbreak frequency was observed. Moreover, in vitro studies suggest a possible antiviral effect of the laser through inhibition of viral replication. 

## Conclusions

 Low-level laser therapy appears to be an effective, safe, and non-invasive alternative for the management of recurrent herpes labialis, particularly in reducing pain and improving clinical outcomes. However, further studies with standardized protocols are required to confirm its impact on recurrence and to establish solid clinical recommendations.

# Abstract: 47 - ORAL HEALTH AND FIBEROPTIC ENDOSCOPIC EVALUATION OF SWALLOWING IN GERIATRIC DYSPHAGIA: AN INTEGRATED MODEL FOR ASPIRATION RISK STRATIFICATION

**Authors:** M.H. Rey Martínez, N. Martínez Rodríguez, M. Andrés Veiga, D. Hurtado Celotti, E. Cañaveral Cavero, L. Sánchez Labrador

**Affiliations:** *San José and Santa Adela Central Red Cross Hospital, Madrid. Email: jelenrei@hotmail.com*

## Introduction

 Oropharyngeal dysphagia is a highly prevalent geriatric condition and a major cause of aspiration pneumonia. While fiberoptic endoscopic evaluation of swallowing allows the identification of aspiration and pharyngeal residue, progression to pulmonary infection depends not only on mechanical factors but also on the bacterial load of the aspirated material. Poor oral health acts as a modifiable determinant of infectious risk. 

## Objectives

 To develop an integrated otolaryngological and dental assessment model for aspiration risk stratification in geriatric patients with dysphagia. 

## Material and Methods

 A structured model is proposed combining fiberoptic endoscopic evaluation of swallowing (assessment of safety, efficiency, pharyngeal residue, and aspiration) with systematic oral health evaluation (edentulism, prosthetic stability, xerostomia, and oral hygiene status). Both dimensions are integrated into a global aspiration risk stratification matrix. 

## Results

 Clinical experience and available evidence suggest that aspiration occurring in the context of good oral health carries a lower infectious risk than apparently safe swallowing in the presence of high bacterial oral load. Combined intervention may reduce aspiration-related complications through intensive oral hygiene, prosthetic optimization, and targeted wallowing rehabilitation. 

## Discussion

 Traditional dysphagia assessment focuses primarily on swallowing safety and mechanical efficiency. However, incorporating systematic oral evaluation enables identification of modifiable biological risk factors and supports preventive strategies, particularly in frail geriatric patients. 

## Conclusions

 Isolated swallowing assessment is insufficient in geriatric dysphagia. Systematic integration of oral health evaluation transforms endoscopic examination into a preventive tool against aspiration pneumonia.

# Abstract: 48 - ARTIFICIAL INTELLIGENCE AS A COMPLEMENTARY TOOL IN THE DETECTION OF ORAL PATHOLOGY THROUGH PANORAMIC RADIOGRAPHY

**Authors:** E. Blanco Novegil

**Affiliations:** *Cooperativa de Ensino Superior Politécnico e Universitário, CESPU. Email: erikablanconovegil26@gmail.com*

## Introduction

 Artificial intelligence is rapidly expanding in the healthcare field, including dentistry. In radiographic analysis, AI can complement the interpretation of panoramic radiographs, contributing to more accurate and earlier detection of potential oral pathologies, directly benefiting both professionals and patients. 

## Objectives

 To analyze the scientific evidence regarding the usefulness of artificial intelligence as a complementary tool in the detection of oral pathology in panoramic radiographs. 

## Material and Methods

 A review of studies published in PubMed over the past year was conducted. These studies evaluated machine learning models applied to the detection of various conditions in panoramic radiographs, including caries, restorations, implants, periapical lesions, bone loss, cysts, and other bone pathologies. 

## Results

 Artificial intelligence models demonstrated high accuracy in detecting various conditions: caries (91.5%), periodontal bone loss (93.09%), and osteoporosis (89.29%). In the detection of periapical lesions, values of up to 99.95% were observed. However, compared to human professionals, AI showed lower performance in certain areas, such as the assessment of periapical status, where false positives were noted. 

## Discussion

: Artificial intelligence is a promising tool in the field of dental radiology, as it can reduce human error and standardize interpretation. However, the evidence presents limitations, such as the lack of well-defined criteria and dependence on image quality. Additionally, there is still considerable skepticism, and not all professionals are willing to assume its costs and learning curve. 

## Conclusions

 Artificial intelligence is a useful and growing tool in the analysis of panoramic radiographs, with the potential for early detection of oral pathology. However, it is essential to integrate it responsibly, without replacing the dentist’s clinical judgment.

# Abstract: 49 - USE OF AUTOLOGOUS DENTIN AS A BIOMATERIAL FOR BONE REGENERATION AND SUBSEQUENT IMPLANT-SUPPORTED REHABILITATION

**Authors:** E. Blanco Novegil

**Affiliations:** *Cooperativa de Ensino Superior Politécnico e Universitário, CESPU. Email: erikablanconovegil26@gmail.com*

## Introduction

 Bone resorption represents a significant clinical challenge in patients requiring oral rehabilitation with dental implants, as it limits the quantity and quality of available bone for proper osseointegration. In recent years, as part of the ongoing scientific effort to develop new solutions to this problem, dentin has emerged as an innovative alternative to other biomaterials. 

## Objectives

 To review the current scientific evidence on the use of autologous dentin as a bone graft and to assess its regenerative efficacy. 

## Material and Methods

 A literature review was conducted based on recently published scientific articles indexed in PubMed regarding the use of dentin as a regenerative material. Methods, clinical, and experimental results were analyzed. 

## Results

 The reviewed studies demonstrate the osteoinductive and osteoconductive properties of dentin, which promote new bone formation. Additionally, the presence of growth factors stimulates bone formation. Dentin exhibits high biocompatibility, reducing the risk of rejection or inflammation and facilitating its integration. 

## Discussion

 Although the results are promising, there are limitations such as: small sample size and a lack of standardization in clinical protocols for dentin processing. Moreover, variability in patient inclusion and exclusion criteria makes comparisons between studies difficult. 

## Conclusions

 Autologous dentin is emerging as a promising material for bone regeneration. Further scientific evidence is needed to support its use and make it more accessible to dentists in clinical practice.

# Abstract: 50 - PRECISION DIAGNOSIS OF ORAL CANCER BASED ON THE ORAL MICROBIOME:A SYSTEMATIC REVIEW

**Authors:** A. Morgade Carneiro, A. Pérez Jardón, A. Sánchez Barco, N. Padrón González, M. Quevedo Bisonni, I. Tomás

**Affiliations:** *Universidad de Santiago de Compostela Email: alvaro.morgade@rai.usc.es*

## Introduction

 Oral cancer (OC) is the sixth most common malignant tumor worldwide. Although histopathological biopsy is the diagnostic gold standard, its limitations have driven the search for alternative Methods that are more accurate, non-invasive, and cost-effective. In this context, recent studies have developed machine learning (ML) models based on the oral microbiome for OC diagnosis. 

## Objectives

 To systematically analyze the diagnostic performance of the oral microbiome in detecting OC using supervised ML models. 

## Material and Methods

 Case–control studies evaluating the oral microbiome through 16S rRNA gene sequencing and reporting diagnostic metrics were included. The search was conducted in July 2025 in PubMed, Scopus, Embase, bioRxiv, and medRxiv. Methodological quality was assessed using QUADAS-2. A qualitative synthesis was performed, distinguishing results between training and validation. 

## Results

 Thirteen studies were included (16–259 participants); 61.5% used saliva, 15.4% mucosal swabs, 15.4% tissue samples, and 7.7% multiple sample types. The most commonly used algorithm was Random Forest (53.8%), with k-fold cross-validation (61.5%) and external validation (53.8%). In training, models (1–94 taxa) showed AUC values ranging from 0.984 to 0.630 and accuracy from 98.17% to 64.86%. Sensitivity reached 100% in some studies, while specificity ranged between 88.27% and 90.00%. In validation, performance remained high (AUC: 0.992–0.364; accuracy: 97.67%–78.57%), with greater variability in sensitivity (100%–65.30%) and specificity (99.40%–61.00%). 

## Discussion and Conclusions

ML models based on the oral microbiome—particularly those using saliva samples and Random Forest—show excellent potential as non-invasive diagnostic tools for OC. However, studies with larger sample sizes and more robust Methodologies are needed to improve generalizability and clinical implementation.

# Abstract: 51 - MANAGEMENT OF TOOTH EXTRACTION IN GERIATRIC PATIENTS ON ANTICOAGULANTS

**Authors:** D. Terán Ruiz, C. García Suárez, J. Alonso Hernández, C. Barrilero Martín, M.A. Alobera, M. del Canto Pingarrón

**Affiliations:** *Master’s Degree in Oral Surgery, Implantology and Periodontology, University of León. Email: david97tr@gmail.com*

## Introduction

 For a long time, vitamin k antagonists (vkas) have been used for systemic anticoagulation in patients with cardiovascular problems, with their use being suspended or not depending on the type of procedure performed on the patient. Depending on the risk of surgical bleeding, dental procedures are divided into those with a high risk of bleeding (surgical or multiple extractions) and those with a low risk of bleeding (rar or simple extractions), which do not usually require the patient’s medication to be discontinued. in oral surgery, the risk of fatal bleeding is low and the risk of thromboembolic events following the interruption of oral anticoagulation is 0.8%; however, such interruption remains a common practice today and is usually accompanied by bridging therapy with low-molecular-weight heparin (lmwh). 

## Objectives

 The objective of this systematic review is to examine the management of treatment and assess the risk of bleeding in patients on oral anticoagulation with direct oral anticoagulants (doacs) and vkas undergoing dental procedures. 

## Results and Discussion

 The risk of bleeding with doacs was estimated to be lower than that with vkas; however, the proportion of patients with atrial fibrillation was significantly higher in the doac group, and the number of patients who received local hemostatic agents in all extraction sockets was significantly higher in the vka group. 

## Conclusions

 A temporary interruption of anticoagulant or antiplatelet therapy for dental surgery does not appear to increase the clinical risk of bleeding or serious procedure-related complications. to reduce the risk of thromboembolic events associated with the interruption of anticoagulants, it is suggested that patients continue taking their doacs without interruption during routine and surgical tooth extractions, provided that local hemostatic measures are applied, although further studies are still needed for dental surgery in patients treated with doacs.

# Abstract: 52 - COMPARATIVE ANALYSIS OF VOLUME, DENSITY, AND HISTOLOGY IN MAXILLARY SINUS EVALUATION USING XENOGRAFT VS. XENOGRAFT + PRGF-ENDORET. A PILOT STUDY

**Authors:** R. Martínez Vilariño, L. Antonio Zancajo, J. De Elio Oliveros, M.A. Alobera García, M. Del Canto Pingarron

**Affiliations:** *Master’s Degree in Surgery, Implantology, and Periodontics. University of León. Email: clinicavilarino@gmail.com*

## Objectives

 To evaluate the potential beneficial effect of PRGF (Plasma Rich in Growth Factors) on bone regeneration regarding graft volume maintenance, density, and new bone formation in maxillary sinus lift procedures. 

## Study design

 The sample consists of 10 patients divided into two groups: Group 1 (NP: 5): Patients treated with xenograft (OsteoBiol Apatos); Group 2 (NP: 5): Patients treated with xenograft (APATOS) + PRGF ENDORET. 

## Material and Methods

 CBCT scans were performed immediately post-surgery and at 18 weeks (T18) to assess changes in bone volume and density. For histological analysis, samples were collected via trephine biopsy 18 weeks after the surgical procedure. A pilot study aimed at comparing and analysing variations in volume, density, and histology using two different filling materials in maxillary sinus floor elevation.

# Abstract: 53 - APPLICATION OF ARTIFICIAL INTELLIGENCE MODELS IN THE DIAGNOSIS OF TEMPO-ROMANDIBULAR DISORDERS

**Authors:** S. Ghawadreh Escudero, A. Luaces Gómez, M.E. Bermejo Blanco, R. Mateos Rodríguez, M.A. Alobera Gracia, M. del Canto Pingarrón

**Affiliations:** *Master’s Degree in Oral Surgery, Implantology and Periodontology. University of León. Email: salmaghawadreh@gmail.com*

## Introduction

 Temporomandibular disorders are a common and complex musculoskeletal condition, whose diagnosis shows variability due to their multifactorial etiology. Artificial intelligence is emerging as a tool to improve diagnostic accuracy through the automated analysis of clinical data and imaging. 

## Aim

 This study aimed to analyze recent scientific evidence on the application of artificial intelligence models in the diagnosis of temporomandibular disorders. 

## Material and Methods

 A systematic review was conducted in PubMed (2021–2026) using the terms “Artificial Intelligence” and Temporomandibular Disorders.” High-quality studies in humans evaluating the diagnostic performance of artificial intelligence using metrics such as accuracy, sensitivity, specificity, and area under the curve were included. After applying selection criteria, 10 studies were included. 

## Results

 Artificial intelligence models showed variable diagnostic performance, with wide ranges of accuracy, sensitivity, specificity, and area under the curve. Deep learning and image-based models achieved the best results, occasionally comparable to human diagnosis. Models combining clinical data demonstrated improved overall performance. High heterogeneity among studies was observed. 

## Discussion

 Evidence suggests that artificial intelligence may improve the diagnosis of temporomandibular disorders, particularly through deep learning and image analysis. However, Methodological heterogeneity, variability, and lack of external validation limit current clinical applicability. More standardized and Methodologically robust studies are required for safe clinical implementation. 

## Conclusions

 Artificial intelligence is a promising tool, although its clinical use requires further evidence, standardization, and external validation.

# Abstract: 54 - EVALUATION OF ARTIFICIAL INTELLIGENCE (AI) IN THE DETECTION OF POTENTIALLY MALIGNANT ORAL LESIONS: COMPARISON WITH CLINICIANS AND ANALYSIS OF THE PROMPT EFFECT

**Authors:** M.A. Sharif Ardestanian, D.M. Amirbagloy, H. Habibollahi, C.M. Cobo Vázquez

**Affiliations:** *Universidad Complutense de Madrid. Email: alishari@ucm.es*

## Introduction

 Early detection of potentially malignant oral lesions is essential to improve the prognosis of oral cancer. In this context, artificial intelligence (AI) has emerged as a promising tool for the analysis of clinical images. 

## Objectives

 To evaluate the diagnostic capability of AI models in detecting potentially malignant oral lesions compared to dental students, as well as to analyze the influence of prompt type on the results. 

## Material and Methods

 A comparative observational study was conducted using six clinical images (three malignant and three benign). Three AI models (ChatGPT, Gemini, and Microsoft Copilot) evaluated each image using three types of prompts (open, structured, and directed), resulting in a total of 54 assessments. In parallel, final-year dental students analyzed the same images. Sensitivity, specificity, and diagnostic accuracy were calculated. 

## Results

 AI achieved an overall sensitivity of 85.2%, specificity of 66.7%, and diagnostic accuracy of 75.9%. In contrast, the students showed a sensitivity of 66.7%, specificity of 30.8%, and accuracy of 48.7%, indicating a tendency toward overdiagnosis. Variability was observed depending on the model and the type of prompt used. 

## Discussion

 AI outperformed students in performance; however, the literature reports mixed results. Some studies indicate that AI surpasses non-specialist clinicians, while performance compared with specialists is comparable. The heterogeneity of models and the influence of prompt type highlight the need to establish standardized protocols. 

## Conclusions

 AI demonstrates a high capability for detecting potentially malignant oral lesions, outperforming the evaluated clinicians. However, its lower specificity and dependence on prompt type highlight the need to standardize its clinical use. AI should be considered a decision-support tool under professional supervision.

# Abstract: 55 - RADIOMORPHOMETRIC PREDICTORS OF DENTAL IMPLANT OUTCOMES IN PATIENTS WITH CHRONIC KIDNEY DISEASE: A RETROSPECTIVE EXPLORATORY COHORT STUDY

**Authors:** J.B. Medina, A.L. Ferreira da Costa, P.H. Braz Silva, A. Blanco Carrión, K. Ortega

**Affiliations:** *Oral Medicine, Oral Surgery and Implantology Unit (MedOralRes), Faculty of Medicine and Dentistry, Universidad de Santiago de Compostela, Santiago de Compostela, Spain. Email: jbm@usp.br*

## Introduction

 Chronic kidney disease is associated with mineral and bone disorders that may compromise bone quality and affect the predictability of dental implant outcomes. Conventional laboratory markers reflect systemic metabolism but do not provide direct information on the structural characteristics of alveolar bone. 

## Objectives

 To investigate the association between clinical, laboratory, and radiomorphometric variables and the utcomes of peri-implant bone loss and implant loss in patients with chronic kidney disease. 

## Material and Methods

 A retrospective exploratory cohort study was conducted including dental implants placed in patients with chronic kidney disease undergoing hemodialysis. Clinical and laboratory data were collected from patient records. Standardized periapical radiographs were analyzed using texture and fractal Methods. Texture features were extracted from gray-level co-occurrence matrices, and principal component analysis was applied to reduce dimensionality. Associations between variables and outcomes were evaluated using non-parametric tests and logistic regression models. 

## Results

 Twenty implants in 15 patients were analyzed, with 79 radiographs. Peri-implant bone loss occurred in 75% of implants, and implant loss in 25%. Implants with bone loss showed higher values of texture parameters associated with structural heterogeneity and lower uniformity. Principal component analysis summarized these variables into a principal component representing global heterogeneity, which remained significantly associated with peri-implant bone loss (OR = 1.63; 95% CI: 1.07–3.09; p = 0.049). No clinical or laboratory variables were associated with the outcomes. Texture measures remained stable over time. 

## Discussion

 The findings suggest that radiographic texture reflects structural characteristics of the local bone phenotype that are not captured by systemic markers and may influence implant outcomes in chronic kidney disease. 

## Conclusions

 Radiographic texture analysis was associated with peri-implant bone loss in chronic kidney disease patients and may serve as an adjunctive tool for risk assessment, although further prospective studies are required.

# Abstract: 56 - ADJUNCTIVE EFFECT OF PROBIOTICS IN PERIODONTAL AND PERI-IMPLANT THERAPY: A REVIEWFrankou Ahmidouch S, Gavarrete Bonilla I, Sanchez Lázaro I, Vega Braga B

**Authors:** S. Frankou Ahmidouch, I. Gavarrete Bonilla, I. Sanchez Lázaro, B. Vega Braga

**Affiliations:** *University of León. Email: sarafrankou@hotmail.com*

## Introduction

 Periodontal and peri-implant therapy is based on mechanical biofilm control, but in many patients periodontal pockets or mucositis persist despite adequate scaling and good oral hygiene. In this context, the use of probiotics has been proposed to regulate the microbiota and the inflammatory response. 

## Objectives

 The aim of this work is to update current knowledge on the eÝect of probiotics in periodontal therapy and peri-implantitis, assessing their impact on periodontal clinical parameters, inflammation and the oral microbiota. 

## Material and Methods

 Articles were searched in the Pubmed and Cochrane databases, limiting the publication date to the last six years. Only human clinical trials were selected, and total of ten articles were included. 

## Results

 In periodontitis, the microorganism used is Limonsilactobacillus reuteri, and no significant improvements in probing depth or clinical attachment gain are shown compared with mechanical therapy alone. Better plaque control, improvement of local inflammation, and a decrease in inflammatory mediators were observed. Microbiological analyses show a slight reduction of certain pathogens and more balanced microbiota. In mucositis, a reduction in bleeding on probing is observed, and peri-implant health parameters improve more significantly in the test group. 

## Conclusions

 Probiotics appear to be useful as adjuvants to modulate inflammation and the microbiota in periodontal and peri-implant therapy, but their eÝect on probing depth and attachment levels remain variable, which limits their usefulness as a routine adjunct to periodontal therapy.

# Abstract: 57 - LONG-TERM SURVIVAL OF FULL-ARCH IMPLANT-SUPPORTED RESTORATIONS USING MONOLITHIC ZIRCONIA: A NARRATIVE REVIEW

**Authors:** S. Prin, X. Liu, M. Albanchez, J. Peláez Rico, M.J. Suárez García

**Affiliations:** *Universidad Complutense de Madrid Email: sofiprin@ucm.es*

## Introduction

 Tooth loss is a significant global problem due to its functional, aesthetic, and systemic impacts, including its potential link to dementia resulting from impaired masticatory function. Rehabilitation using monolithic zirconia implants offers excellent physical, mechanical, and biological properties. Their CAD/CAM fabrication has helped reduce issues such as chipping and fractures. 

## Objectives

 To evaluate the survival rate and clinical success of monolithic zirconia in full-arch implant-supported restorations. 

## Material and Methods

 A literature search was conducted in the PubMed, Cochrane, and Scopus databases using keywords such as “monolithic zirconia,” “full arch,” “complete arch,” “implant- supported,” and “survival.” Of the initial 54 articles, 8 were selected after applying inclusion and exclusion criteria. 

## Results

 Survival rates ranged from 88% to 100% at follow-up periods of 1 to 7 years, with implant survival approaching 100%. A low incidence of fractures was observed, particularly on occlusal surfaces. However, complications such as fractures of the veneering material and in screw access areas were reported. Some studies reported a higher complication rate compared to metal-ceramic restorations, although these were less frequent in older patients. 

## Discussion

 The main complications included fractures of the veneer and the abutment structure. These were more common in younger patients, possibly due to greater masticatory forces. 

## Conclusions

 Monolithic zirconia yields favorable results in implant-supported restorations in the short and medium term. However, longitudinal studies with larger sample sizes are needed to confirm its long-term performance.

# Abstract: 58 - COMPARISON OF MECHANICAL STRENGTH BETWEEN 3D-PRINTED RESINS AND THERMOPOLYMERIZED POLYMETHYL METHACRYLATE IN COMPLETE DENTURES: A NARRATIVE REVIEW

**Authors:** J.P. Ruano Guevara, A. Almojahed, P. Sevilla, J. Peláez Rico, M.J. Suárez García

**Affiliations:** *Master’s Degree in Oral and Maxillofacial Prosthetics and Occlusion. Complutense University of Madrid Email: juanpablorg4@gmail.com*

## Introduction

 The rehabilitation of patients with complete edentulism involves a series of procedures during treatment, with the result being a key factor in treatment success and patient satisfaction. Mechanical strength remains a determining factor in the performance of these materials, especially in geriatric patients. 

## Objectives

 Compare the mechanical strength of 3D-printed resins with thermopolymerized polymethyl methacrylate (PMMA) in complete dentures. 

## Material and Methods

 An electronic literature search was conducted in the PubMed, Scopus, and Cochrane databases for in vitro and experimental studies published in the last 5 years that evaluated flexural strength and mechanical properties of PMMA and 3D-printed materials. The following keywords were used: “3D printed,” “PMMA,” “flexural strength,” “denture base,” and “mechanical properties.” Out of 39 articles, 13 were selected after applying inclusion and exclusion criteria. 

## Results

 The studies included shows that PMMA has better mechanical properties compared to 3D-printed materials. Furthermore, it was observed that flexural strength is higher in the conventional technique compared to 3D-printed materials, and this relates to the manufacturing process, which requires post-clinical evaluations. 

## Discussion

 3D-printed materials are an alternative due to their manufacturing and production Methodss; however, they do not possess the same mechanical properties as thermopolymerized PMMA, which is a very important factor when subjected to functional loads, making it a key consideration in the appropriate selection of materials. 

## Conclusions

 Thermopolymerized PMMA remains the material with the best mechanical properties for complete dentures. Although 3D-printed materials are a suitable alternative in various clinical situations, further studies are needed to support their long term use.

# Abstract: 59 - ELDERLY PATIENTS WITH TRIGEMINAL NEURALGIA: A RETROSPECTIVE STUDY IN AN ORAL MEDICINE CLINIC

**Authors:** I. Santareno, M. Machete, R. Cacodcar, J. Barranco, P. Trancoso, A. Mano Azul

**Affiliations:** *Egas Monis Hospital Email: inesantareno@gmail.com*

## Introduction

 The rehabilitation of patients with complete edentulism involves a series of procedures during treatment, with the result being a key factor in treatment success and patient satisfaction. Mechanical strength remains a determining factor in the performance of these materials, especially in geriatric patients. 

## Objectives

 Compare the mechanical strength of 3D-printed resins with thermopolymerized polymethyl methacrylate (PMMA) in complete dentures. 

## Material and Methods

 An electronic literature search was conducted in the PubMed, Scopus, and Cochrane databases for in vitro and experimental studies published in the last 5 years that evaluated flexural strength and mechanical properties of PMMA and 3D-printed materials. The following keywords were used: “3D printed,” “PMMA,” “flexural strength,” “denture base,” and “mechanical properties.” Out of 39 articles, 13 were selected after applying inclusion and exclusion criteria. 

## Results

 The studies included shows that PMMA has better mechanical properties compared to 3D-printed materials. Furthermore, it was observed that flexural strength is higher in the conventional technique compared to 3D-printed materials, and this relates to the manufacturing process, which requires post-clinical evaluations. 

## Discussion

 3D-printed materials are an alternative due to their manufacturing and production Methods; however, they do not possess the same mechanical properties as thermopolymerized PMMA, which is a very important factor when subjected to functional loads, making it a key consideration in the appropriate selection of materials. 

## Conclusions

 Thermopolymerized PMMA remains the material with the best mechanical properties for complete dentures. Although 3D-printed materials are a suitable alternative in various clinical situations, further studies are needed to support their long term use.

# Abstract: 60 - IMPACT OF HYALURONIC ACID TREATMENT ON QUALITY OF LIFE AND PSYCHOSOCIAL PERCEPTION: A CLINICAL CASE

**Authors:** A. Pérez Barrionuevo, E. Gonzalo

**Affiliations:** *Master de Prótesis Bucofacial y Oclusión. Universidad Complutense de Madrid Email: antonio.perez.barrionuevo@gmail.com*

## Introduction

 Aging involves facial and perioral changes that can significantly affect self-esteem, social interaction, and quality of life in older patients. In recent years, the use of hyaluronic acid in the perioral area has increased; however, there is limited evidence regarding its psychosocial impact in this population group. 

## Objectives

 To evaluate the impact of hyaluronic acid treatment on quality of life and psychosocial perception in older patients. 

## Case Report

 We present the case of a 74-year-old patient who came to the clinic reporting dissatisfaction with her perioral aesthetics, associated with decreased self-esteem and avoidance of social situations. After evaluation, clinical treatment with hyaluronic acid in the perioral region was planned with the aim of improving lip support and facial armony in preparation for dental treatment. Quality of life was assessed using a questionnaire and subjective scales of aesthetic perception and self-esteem four weeks after treatment. 

## Results

 After treatment, an improvement was observed in quality of life indices, as well as in aesthetic perception and self-esteem. The patient reported greater satisfaction with her image and improved social interaction. No relevant adverse effects were recorded. 

## Discussion

 This case illustrates how hyaluronic acid treatments in older patients can transcend the aesthetic component, contributing significantly to emotional and social well-being. 

## Conclusions

 These results support its use as a therapeutic tool within a comprehensive approach to geriatric patients. The incorporation of minimally invasive aesthetic treatments can contribute to healthy aging, improving not only appearance but also the emotional and social well-being of older patients.

# Abstract: 61 - INTRAOPERATIVE DISPLACEMENT OF A DENTAL IMPLANT INTO THE MAXILLARY SINUS: NARRATIVE REVIEW AND PRESENTATION OF A CLINICAL CASE TREATED WITH A CALDWELL-LUC APPROACH

**Authors:** P. Maguregui Ortiz, A. García Marqués, P. Benito López, S. De Córdoba, S. De La Vega, I. Leco Berrocal

**Affiliations:** *Posgrado de Especialización en Cirugía Bucal e Implantología. Facultad de Odontología. Universidad Complutense de Madrid, Email: pabmagur@ucm.es*

## Introduction and objectives

 Displacement of dental implants into the maxillary sinus is an uncommon but potentially serious complication that may lead to sinus pathology. The aim of this study is to review the main causes, diagnostic approaches, and treatment options for this condition, as well as to present a clinical case. 

## Material and Methods

 A narrative review of the relevant scientific literature on implant displacement into the maxillary sinus was performed. We report the case of a 65-year-old patient with a history of maxillary sinus lift, in whom intraoperative displacement of a dental implant occurred during placement. Diagnosis was established by computed tomography, and surgical removal was carried out using a Caldwell-Luc approach. 

## Results and Discussion

 The literature identifies residual bone deficiency, previous sinus lift procedures, and lack of primary stability as the main risk factors. The Caldwell-Luc approach allows direct visualization and controlled removal of the implant. In the present case, no signs of sinusitis were observed, and the postoperative course was uneventful, with no complications. 

## Conclusions

 Intraoperative displacement of dental implants into the maxillary sinus requires prompt management. The Caldwell-Luc approach remains a safe and effective option, particularly in patients with a history of sinus surgery. Proper preoperative planning and achieving adequate primary stability are essential to prevent this complication.

# Abstract: 62 - USE OF THE DOUBLE-FACTOR TECHNIQUE FOR SINGLE IMPLANT PLACEMENT

**Authors:** A. Bowen Varela, S. García Rodrñiguez, J. iménez Aracil, A. Martínez Romero, C. Belarra Arenas, M. Sanmartín Oliver

**Affiliations:** *Complutense University of Madrid. Email: albowen@ucm.es*

## Introduction

 The use of computed tomography and digital planning has transformed implant surgery. Two main Methods allow the transfer of digital planning to the surgical procedure: static computer-assisted implant surgery (sCAIS) and dynamic navigation systems (d-CAIS). The so-called double-factor technique integrates both systems, combining the mechanical stability of static surgical guides with real-time dynamic control. This integration improves the three-dimensional positioning of the implant and may reduce cumulative surgical errors, particularly in complex anatomical situations. 

## Objectives

 To present a clinical case of single implant placement using the double-factor technique, highlighting its clinical advantages and surgical precision. 

## Case Report

 An 80-year-old patient with no relevant medical history attended the clinic for implant placement. Treatment planning was performed using CBCT imaging and digital planning software. The surgery was carried out using dynamic navigation (Navident Evo 4.0) combined with a surgical guide. 

## Results

 The technique allowed precise implant placement according to the digital planning, with correct three dimensional orientation and minimal surgical invasiveness. Implant integration was favorable and a stable provisional restoration was achieved. 

## Discussion

 The literature shows increasing interest in the double-factor technique. Dynamic navigation improves surgical accuracy and may reduce operative trauma while promoting better postoperative recovery. However, its implementation still presents limitations related to cost and the learning curve. 

## Conclusions

 The combination of static guides and dynamic navigation can reduce the chain of errors in digital implant workflows. Initial clinical results are promising, although further controlled studies and cost-effectiveness analyses are required. The double-factor technique should be considered a high-precision strategy for selected clinical cases.

# Abstract: 63 - PROTOCOL FOR THE MANAGEMENT OF GERIATRIC PATIENTS ON ORAL BISPHOSPHONATES UNDERGOING ORAL SURGERY: CLINICAL CASE AND LITERATURE REVIEW

**Authors:** A. Martínez Romero, P. Revuelta Cortés, P. Maguregui Ortiz, K. Ivaylova Serkedzhieva, F. González-Fernández Tresguerres, J. Cortés-Bretón Brinkmann

**Affiliations:** *Postgraduate Program in Oral Surgery and Implantology. Complutense University of Madrid Email: amarti37@ucm.es*

## Introduction

 Bisphosphonates are used in the treatment of osteoporosis and other bone diseases and are associated with the development of medication-related osteonecrosis of the jaw (MRONJ) following surgical procedures. This risk is increased in geriatric patients due to comorbidities and polypharmacy.

## Objectives

 To establish a management protocol for geriatric patients receiving oral bisphosphonates who undergo oral surgery, in order to minimize complications and optimize healing. 

## Case Report

 A 77-year-old patient undergoing treatment with bisphosphonates for osteoporosis (weekly alendronic acid for 8 months), with a history of polypharmacy. A preventive protocol was implemented based on antibiotic therapy, atraumatic surgical technique, collection and application of platelet-rich fibrin, and primary closure. Extraction of an impacted lower third molar was performed. Postoperative treatment included antibiotics, analgesics, and oral hygiene control. 

## Results

 Clinical evolution was favorable, with no signs of MRONJ or infection, showing adequate soft tissue healing and no complications during follow-up.

## Discussion

 The literature highlights the importance of preventing MRONJ in patients treated with bisphosphonates. Factors such as the concomitant use of antiangiogenic agents, Immunemo-dulators, or corticosteroids, as well as the type and duration of treatment, mandibular location, poor oral hygiene, and habits such as smoking or alcohol consumption, influence the risk. Minimally invasive surgical protocols improve prognosis. 

## Conclusions

 Protocolized management in geriatric patients treated with bisphosphonates minimizes the risk of MRONJ and promotes a favorable clinical outcome.

# Abstract: 64 - SURGICAL MANAGEMENT OF PERI-IMPLANTITIS; A CASE REPORT

**Authors:** D. Sánchez Cousiño, R. Ezzedine Doughan, A. Roselló Camps, M. Allegue Allegue, B. González Navarro, J. López López

**Affiliations:** *Master's Degree in Oral Medicine, Surgery and Implantology. Faculty of Medicine, Health Sciences, Dentistry, University of Barcelona Email: dsanchez11052000@gmail.com*

## Introduction

 Peri-implantitis is a frequent complication in full-arch implant-supported restorations, characterized by peri-implant inflammation and progressive bone loss. Its management remains a clinical challenge, especially in patients with systemic risk factors or a history of smoking. 

## Objectives

 To describe the surgical approach to peri-implantitis in a full mandibular restoration using implantoplasty and to evaluate its clinical efficacy. 

## Case Report

 A 65-year-old woman, with a history of hypothyroidism and a former smoker (15 cigarettes/day, quit 3 years prior), had a full-arch upper and lower prosthesis. The lower prosthesis, placed years earlier, presented clinical and radiographic signs consistent with peri-implantitis. Resective surgical treatment was performed, including mechanical and chemical debridement, implantoplasty to modify the exposed surface, irrigation with hypochlorous acid, and placement of PRF membranes. 

## Results

 Following treatment, a reduction in bleeding on probing and an improvement in inflammatory clinical parameters were observed. The literature indicates that mechanical Methods have limited efficacy against invasive bacteria, with air polishing using glycine or erythritol being a less abrasive and effective alternative for submucosal biofilm. Chemical agents such as chlorhexidine do not show significant improvements in probing depth compared to scaling alone, although local antibiotics may improve clinical parameters in the short term. The use of lasers or adjuvant therapies has not demonstrated stable long-term benefits. 

## Discussion

 In cases with established bone loss, non-surgical treatment has limitations, justifying a surgical approach. Implantoplasty allows for a reduction in surface roughness and hinders bacterial recolonization. The combined use of chemical agents and PRF could promote decontamination and healing, although the evidence remains limited. 

## Conclusions

 Surgical treatment with implantoplasty is an effective option in the management of advanced peri-implantitis, allowing for improvement of clinical parameters, although further studies are required to establish standardized protocols.

# Abstract: 65 - INJURY TO THE LATERAL BORDER OF THE TONGUE: A CASE REPORT

**Authors:** S. Clos i Soler, M. Apoita Sanz, M. Duch Balust, A. Mari Roig, B. Gonzaìlez Navarro, J. Lopez Lopez

**Affiliations:** *Master's Degree in Oral Medicine, Surgery and Implantology. Faculty of Medicine, Health Sciences, Dentistry, University of Barcelona Email: silviaclos7@hotmail.com*

## Introduction

 Oral squamous cell carcinoma is the most common malignant tumor of the oral cavity, representing a public health concern due to its high morbidity and mortality. It is frequently located on the lateral border of the tongue, an area considered high risk because of its greater predisposition to malignant transformation. 

## Objectives

 To emphasize the importance of early diagnosis in order to detect lesions of the oral cavity at an early stage. Clinical Case: A 51-year-old male patient presented with an incidentally discovered lesion on the lateral border of the tongue, with no associated trauma. Examination revealed a single plaque-like lesion on the left lateral border of the tongue, with an erythroleukoplastic appearance, smooth surface, indurated consistency, and well-defined borders, accompanied by pain and with an evolution of more than 2 weeks. A differential diagnosis was established including white lesions such as leukoplakia and frictional keratosis, as well as red lesions such as erythroplakia. After complementary tests, the patient was referred to the Maxillofacial Surgery Department at Bellvitge Hospital for a biopsy.

## Results

 Following the biopsy, the patient was diagnosed with infiltration by well-differentiated squamous cell carcinoma, which was subsequently treated with partial glossectomy and sentinel lymph node excision. 

## Discussion

 Despite presenting a cancerous lesion at a relatively young age, as in this case, it is important to carefully examine the oral mucosa in order to perform an adequate clinical history. Squamous cell carcinoma is more prevalent in males and is associated with alcohol and tobacco use, with the tongue being its most frequent location. 

## Conclusions

 The role of the dentist, as well as a multidisciplinary approach, is of vital importance in the early diagnosis of oral squamous cell carcinoma, given its high potential for malignancy.

# Abstract: 66 - MANAGEMENT OF OROANTRAL COMMUNICATION AFTER MAXILLARY MOLAR EXTRACTION: A CLINICAL CASE REPORT

**Authors:** A. Toribio Méndez, A. Borda Carrasco, R. Verónica Schiavo Ezzedine Doughan, B. González Navarro, J. López López

**Affiliations:** *Master's Program in Oral Medicine, Surgery and Implantology - University of Barcelona - Faculty of Medicine and Health Sciences [Dentistry]. Email: adri7toribio@gmail.com*

## Introduction

 An orosinus communication is a relatively common complication in oral surgery, particularly following the extraction of upper molars due to their anatomical proximity to the maxillary sinus. Early diagnosis and treatment are essential to prevent complications such as maxillary sinusitis or fistula formation. 

## Objectives

 To describe the clinical management of an orosinus communication following tooth extraction and to evaluate the efficacy of primary closure using a vestibular advancement flap. 

## Clinical Report

 A 67-year-old female patient with a medical history of hypertension and ischemic heart disease presented for extraction of tooth 2.6 due to pain. During the procedure, an orosinus communication measuring approximately 6 x 3 millimeters occurred. Immediate closure was performed using a vestibular advancement flap with drainage incisions and periosteal release to achieve tension-free closure. Postoperative measures and pharmacological treatment were prescribed. 

## Results

 The closure was clinically satisfactory. During follow-up, the patient showed favorable progress, with no signs of infection or persistent communication. At three weeks, she was asymptomatic and the tissues had healed properly.

## Discussion

 The size of the defect and the speed of treatment are determining factors in the prognosis. Small defects may resolve spontaneously, while larger ones require surgical treatment. The vestibular advancement flap is a simple and effective technique for small and moderate defects, although other surgical options may be indicated in more complex or larger cases. 

## Conclusions

 Immediate closure using a vestibular advancement flap is an effective option for the treatment of moderate-sized orosinus fistulas. Early diagnosis and appropriate management help prevent complications and ensure a favorable clinical outcome.

# Abstract: 67 - BULLOUS LESIONS: A CASE REPORT

**Authors:** F. Arellano, H. Mendiola, A. Pellicer, E. Jane, B. Gonzales, J. López

**Affiliations:** *Master’s degree in Medicine, Surgery and Oral Implantology - University of Barcelona Email: fernando.10.80@hotmail.com*

## Introduction

 Bullous lesions of the oral mucosa are uncommon but clinically relevant entities, generally associated with autoimmune diseases, which present as vesicles or painful blisters with a tendency to ulcerate. Among the main conditions are pemphigus and pemphigoid, and they may be related to systemic immunological factors such as viral infections and thyroid disorders. 

## Case Report

 A 79-year-old patient was referred from primary care due to oral lesions, with a medical history of hypothyroidism and arterial hypertension, and allergy to sulfonamides. In the intraoral examination, multiple lesions compatible with ulcerated blisters were observed, located on the attached gingiva and floor of the mouth, erythematous, variable in size, homogeneous in appearance, irregular surface, well-defined margins, and sessile base. The patient reports periocular involvement following COVID-19 for approximately 2 years, without a definitive diagnosis, prior to the onset of the oral lesions. To confirm the nature of the oral lesions, an incisional biopsy was performed, establishing the diagnosis of pemphigoid. Multidisciplinary management was initiated with topical triamcinolone 0.2% in a tapering regimen over 4 weeks, aimed at symptomatic control and adjustment to the minimum effective dose, along with referral to specialists for prevention of systemic sequelae. 

## Discussion

 The literature was reviewed to assess the possible association between bullous lesions and the patient’s systemic history, suggesting that COVID-19 infection and thyroid disorders could act as associated immunological factors, although without evidence of a direct causal relationship. 

## Conclusions

 This case highlights the importance of early dental diagnosis of autoimmune bullous lesions and the need to assess possible associated systemic factors within a comprehensive clinical approach.

# Abstract: 68 - BEYOND THE USUAL: ORAL LICHEN PLANUS IN A MALE PATIENT

**Authors:** A. Barceló, B. Tejedor, R. Cabezas, S. Egido, B. González, M. Blazquez, J. López

**Affiliations:** *Faculty of Medicine and Health Sciences [Dentistry] – Master’s Degree in Oral Medicine, Surgery and Implantology – University of Barcelona. Email: alexiabarceloo@gmail.com*

## Introduction

 The diagnosis of white lesions of the oral mucosa requires careful clinical evaluation due to their variability and potential for malignant transformation. Oral lichen planus (OLP) is a chronic inflammatory disease with different clinical presentations. 

## Case Report

 A 74-year-old male patient with a history of moderate bilateral carotid stenosis, lacunar stroke, dyslipidemia, hypertension, colonic polyposis, and benign prostatic hyperplasia. The patient was under treatment with acetylsalicylic acid, atorvastatin, omeprazole, tamsulosin, lercanidipine, and cholecalciferol. Former smoker for 10 years, with a history of prolonged tobacco use for 47 years and regular alcohol consumption (≈18 standard drinks/week). The patient reported minimal symptoms, limited to mild discomfort when consuming acidic foods. Intraoral examinations revealed multiple bilateral lesions. At the retromolar trigone, atrophic areas with diffuse erythema surrounded by Wickham striae were observed. In the edentulous mandibular alveolar mucosa, diffuse erythema with atrophic areas of smooth and shiny appearance was noted, related to the implant-supported area, without signs of suppuration. In the buccal mucosa and maxillary alveolar mucosa, white lesions with Wickham striae were observed. The lesions were non-scrapable and symmetrically distributed. 

## Discussion

 The differential diagnosis includes leukoplakia, candidiasis, and lichenoid lesions. Bilaterality, symmetry, and the presence of Wickham striae strongly support OLP. The absence of lesion detachment upon scraping rules out candidiasis. Although proliferative verrucous leukoplakia could be considered, the absence of verrucous or exophytic lesions makes it unlikely. However, a drug-induced lichenoid reaction cannot be completely excluded. 

## Conclusions

 OLP is more common in women; therefore, its presentation in men is less frequent. Atrophic/erosive forms, together with tobacco and alcohol consumption, require periodic clinical follow-up due to their potential for malignant transformation.

# Abstract: 69 - REHABILITATION WITH SHORT IMPLANTS: A CASE REPORT

**Authors:** G. Viñas Usán, M. Mesquida Alcover, B. González Navarro, E. Jané Salas, J. López López

**Affiliations:** *Faculty of Medicine and Health Sciences [Dentistry] – Master’s Degree in Oral Medicine, Surgery and Implantology – University of Barcelona. Email: gerardvinas@hotmail.com*

## Introduction

 Implant-prosthetic rehabilitation in posterior mandibular regions with limited bone height represents a common clinical challenge. Traditionally, vertical bone regeneration has been the technique of choice to allow placement of conventional implants. However, the use of short implants has emerged as a less invasive alternative, with reduced morbidity and shorter treatment times. 

## Objectives

 To compare, in the context of a clinical case, the feasibility and predictability of short implants versus bone regeneration techniques in the fourth mandibular quadrant. 

## Case Report

 An 85-year-old female patient (ASA III) presented with the desire for fixed rehabilitation. She showed partial posterior edentulism in the fourth quadrant, with residual roots and vertical bone loss. Following the hygienic phase and extractions, rehabilitation was planned using implants in positions 4.5, 4.6, and 4.7. Bone regeneration was considered versus the placement of short implants, opting for reduced-length implants using an early delayed protocol to improve primary stability and tissue management. 

## Results

 The placement of short implants avoided complex regenerative procedures, reducing surgical risk. Adequate primary stability and proper osseointegration were achieved, with favorable load distribution and no significant postoperative complications. 

## Discussion

 Short implants represent a predictable alternative in atrophic posterior mandibular regions, especially in elderly patients or those with comorbidities. Compared to bone regeneration, they present lower morbidity and simplify treatment, although proper prosthetic planning is required to optimize biomechanics. 

## Conclusions

 In the fourth mandibular quadrant with limited bone availability, short implants represent an effective and safe alternative to bone regeneration, allowing for functional and predictable outcomes with reduced surgical complexity.

# Abstract: 70 - DIAGNOSIS AND MANAGEMENT OF ATROPHIC–ERYTHEMATOUS ORAL LICHEN PLANUS ON THE VESTIBULAR GINGIVA: A CLINICAL CASE PRESENTATION

**Authors:** Z. Halawa Nehad, D. Sánchez Gimeno, F. Ribas March, E. Jané Salas, B. González Navarro, J. López López

**Affiliations:** *Faculty of Medicine and Health Sciences [Dentistry] – Master’s Degree in Oral Medicine, Surgery and Implantology – University of Barcelona. Email: zeyadhalawa@hotmail.com*

## Introduction

 Oral lichen planus (OLP) is a chronic, inflammatory, autoimmune disease mediated by CD8+ T lymphocytes. It has an estimated prevalence of 1–2% in the population. Several clinical forms exist: reticular, atrophic-erythematous, and erosive. The atrophic-erosive form is characterized by well-defined erythematous areas, often associated with epithelial atrophy and symptoms such as a burning sensation or pain. Diagnosis is mainly clinical; however, histopathological confirmation through biopsy is essential to rule out dysplasia or malignant transformation. 

## Objectives

 The aim of this case is to present the diagnosis, treatment, and follow-up of a red lesion on the vestibular gingiva in a patient with hypertension. 

## Case Report

 A 67-year-old female patient with a history of arterial hypertension and no known allergies presented with a symptomatic lesion on the mandibular gingiva. The patient reported a burning sensation and mild pain during tooth brushing. Intraoral examination revealed a well-defined erythematous area located between teeth 1.3 and 2.3, on the vestibular gingiva of the anterior mandibular region, with an atrophic appearance and absence of typical white reticular striae. Given the clinical suspicion of atrophic-erythematous oral lichen planus, an incisional biopsy was performed under local anesthesia to confirm the diagnosis. Postoperative management included the prescription of a compounded formulation containing 0.2% triamcinolone acetonide, along with clinical follow-up.

## Results

 The biopsy confirmed oral lichen planus, with no signs of malignancy. The patient experienced improvement in the burning sensation, and the erythematous lesion resolved after 5 weeks of treatment.

## Conclusions

 Oral lichen planus affecting the gingiva can cause discomfort. Biopsy is necessary to confirm the diagnosis. Treatment with corticosteroids was effective in resolving the symptoms.
